# Analogous Convergence of Sustained and Transient Inputs in Parallel On and Off Pathways for Retinal Motion Computation

**DOI:** 10.1016/j.celrep.2016.02.001

**Published:** 2016-02-18

**Authors:** Matthew J. Greene, Jinseop S. Kim, H. Sebastian Seung, EyeWirers

**Affiliations:** 1 Brain and Cognitive Sciences Department, Massachusetts Institute of Technology, Cambridge, MA 02139, USA; 2 Princeton Neuroscience Institute, Princeton University, Princeton, NJ 08544, USA; 3 Computer Science Department, Princeton University, Princeton, NJ 08544, USA; 4 http://eyewire.org; 5 Present address: Department of Structure and Function of Neural Networks, Korea Brain Research Institute, Daegu 700-300, Republic of Korea; 6 Co-first author

## Abstract

Visual motion information is computed by parallel On and Off pathways in the retina, which lead to On and Off types of starburst amacrine cells (SACs). The approximate mirror symmetry between this pair of cell types suggests that On and Off pathways might compute motion using analogous mechanisms. To test this idea, we reconstructed On SACs and On bipolar cells (BCs) from serial electron microscopic images of a mouse retina. We defined a new On BC type in the course of classifying On BCs. Through quantitative contact analysis, we found evidence that sustained and transient On BC types are wired to On SAC dendrites at different distances from the SAC soma, mirroring our previous wiring diagram for the Off BC-SAC circuit. Our finding is consistent with the hypothesis that On and Off pathways contain parallel correlation-type motion detectors.

## In Brief

Greene et al. find that in the mouse retina, sustained and transient On bipolar cell (BC) types are wired to dendrites of On starburst amacrine cells (SACs) at different distances from the SAC soma. This wiring specificity may support retinal computation of the direction of a moving stimulus.

## Graphical Abstract

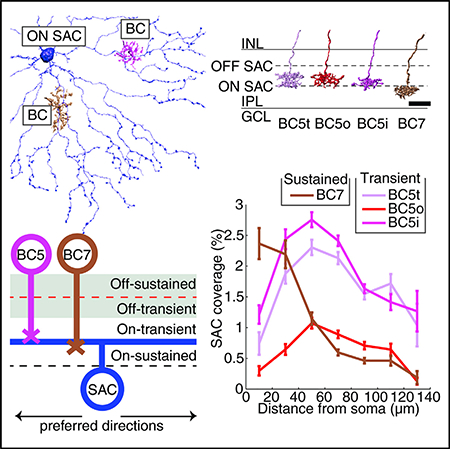

## INTRODUCTION

The starburst amacrine cell (SAC) is a key player in retinal computation of the direction of a moving stimulus. Ablation of SACs impairs the optokinetic reflex, a behavior that depends on computation of visual motion (Yoshida et al., 2001; Amthor et al., 2002). Both ablation (Yoshida et al., 2001; Amthor et al., 2002) and reversible inactivation (Vlasits et al., 2014) of SACs reduce direction selective (DS) responses in ganglion cells, which receive synaptic input from SACs. SAC dendrites are preferentially activated by visual stimuli that move outward from the soma to the dendritic tips (Euler et al., 2002; Lee and Zhou, 2006; Hausselt et al., 2007).

The proposed mechanisms for DS of SAC dendrites fall into several categories. According to inhibitory cellular hypotheses, dendritic biophysics causes inhibitory input to SACs to have effects that depend on dendritic location(Borg-Grahamand Grzywacz, 1992; Gavrikov et al., 2003). In inhibitory circuit hypotheses, GABAergic synaptic connectivity between SAC dendrites depends on the difference between their preferred directions (Lee and Zhou, 2006; Münch and Werblin, 2006). In excitatory cellular hypotheses, SAC biophysics causes excitatory input to SACs to have effects that depend on dendritic location (Tukker et al., 2004; Hausselt et al., 2007; Oesch and Taylor, 2010).

Recently, we proposed an excitatory circuit hypothesis based on specificity of wiring between bipolar cells (BCs) and SACs. The proposal was based on anatomical evidence that sustained and transient BC types are connected to SACs at locations that are near and far from the SAC soma, respectively (Kim et al., 2014). Such “space-time wiring specificity” could make the BC-SAC circuit function as a correlation-type motion detector (Borst and Euler, 2011) and is consistent with the observed outward preferred direction of SAC dendrites.

**Figure 1.**
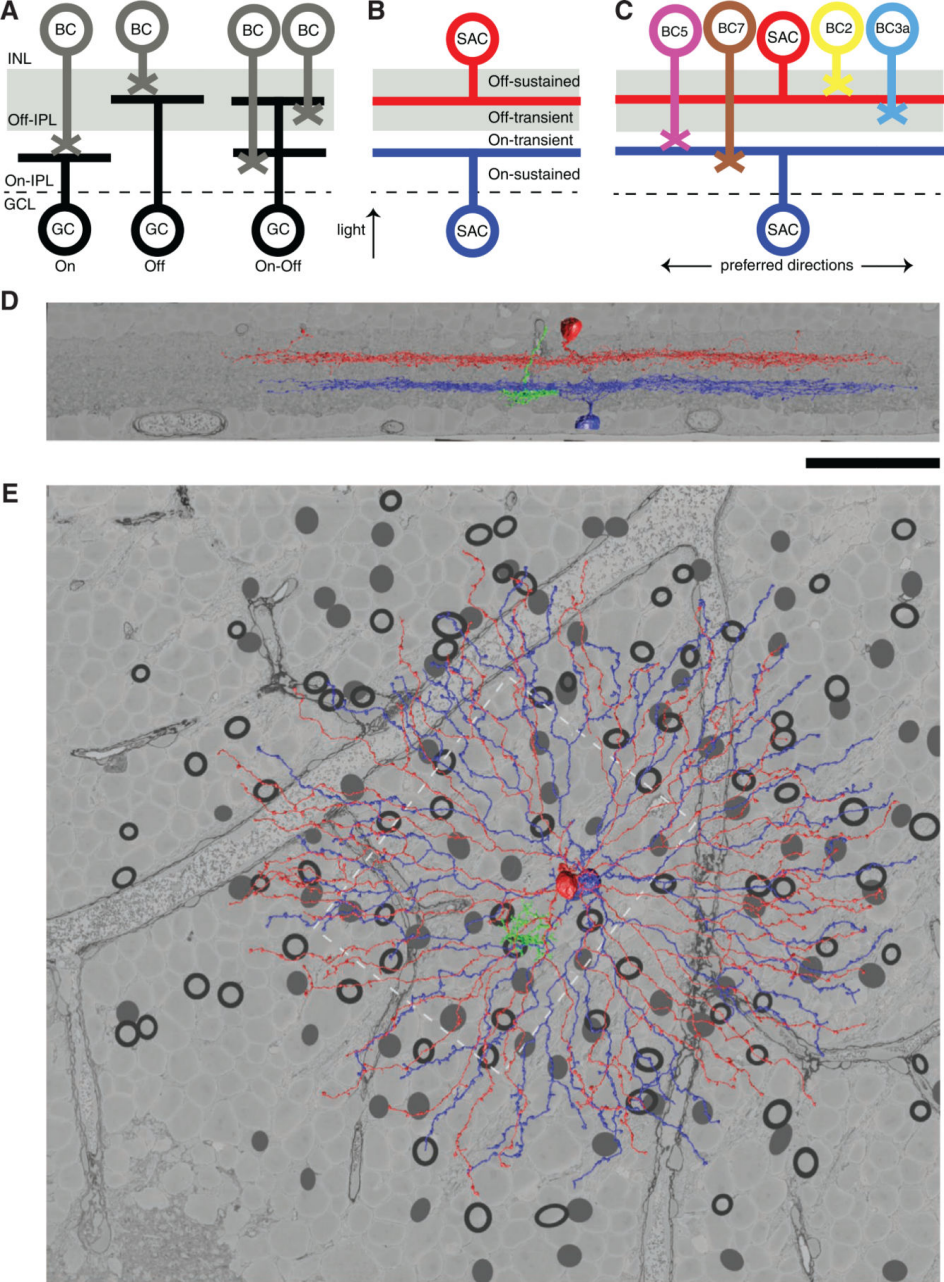
On-Off and Sustained-Transient Organization of the IPL (A) The IPL is divided into On and Off (gray shading) sublayers, which support On BC-GC wiring and Off BC-GC wiring specificity. (B) Observations of BC visual responses (Baden et al., 2013; Borghuis et al., 2013) suggest a tentative further division of the IPL into On-sustained, On-transient, Off-transient, and On-sustained sublayers. The two sustained-transient divisions are located at the depths of On and Off SACs. (C) Analogous wiring specificity for On and Off BC-SAC circuits. Sustained BCs prefer the proximal zone of SACs and transient BCs prefer the intermediate or distal zones of SACs. (D) When viewed perpendicular to the light axis, On (blue) and Off (red) SACs appear mirror symmetric across the plane separating the On and Off sublayers of the IPL. (E) The same SACs appear similar when viewed along the light axis. A BC axon (green) is much smaller. The black circles and gray dots respectively indicate reconstructed On and Off SAC cell body locations. The On BCs were reconstructed in a patch (dashed rectangle). The scale bar represents 50 μm.

Like many other retinal neurons, the SAC comes in both On and Off types. The On SAC resembles a reflection of the Off SAC across a plane through the middle of the inner plexiform layer (IPL) (Figures 1B and 1D). Probably due to this striking symmetry, DS and its mechanisms are often assumed to be similar between On and Off SACs. However, published studies of SACs were typically restricted to a single type. Physiological studies of DS were carried out for On SACs (Euler et al., 2002; Lee and Zhou, 2006; Hausselt et al., 2007), while our anatomical study of BC-SAC wiring specificity was carried out for Off SACs (Kim et al., 2014).

Here, we find evidence that the On BC-SAC circuit possesses a space-time wiring specificity analogous to that shown previously for the Off BC-SAC circuit. We reconstructed a large set of On BCs and On SACs from e2198, a dataset of mouse retinal images from serial block-face scanning electron microscopy (Briggman et al., 2011). Based on the resulting high-resolution information about the anatomy of single cells, we have succeeded in subdividing BC5 into three types that we call BC5t, BC5i, and BC5o. This finding confirms Helmstaedter et al. (2013), who were previously able to distinguish just two BC5 types, but predicted the existence of more. Our definition of a third BC5 type increases the total count of cone BC types to 13.

Contact analysis is consistent with a wiring diagram in which BC7 prefers to synapse closer to the On SAC soma and BC5 prefers to synapse farther from the soma (Figure 1C). Among the BC5 types, synaptic input from BC5o is likely to be less than from BC5t and BC5i.

Most of the available evidence suggests that transient BC types generally arborize near the IPL center and sustained BC types near the IPL edges (Baden et al., 2013; Borghuis et al., 2013) (but see Ichinose et al., 2014 for a divergent view). Combined with the standard division of the IPL into On and Off sublamina, this yields four sublayers: On-sustained, On-transient, Off-transient, and Off-sustained (Figure 1B). Based on this IPL organization, it is likely that BC7 is sustained and BC5 is transient. If this is the case, then On BC-SAC wiring is analogous to Off BC-SAC wiring.

## RESULTS

### Aligning Cells to a Common Coordinate System

The cell bodies of On and Off SACs are in the ganglion cell layer (GCL) and inner nuclear layer (INL) respectively, on opposite sides of the IPL (Figures 1B and 1D). The reconstructions include 156 SACs (77 On and 79 Off; Figure 1E), which we estimate is more than half of the SACs in e2198 (Experimental Procedures). The diameter of the SAC arbor is much larger than the spacing between cell bodies (Figure 1E), so the arbors of adjacent SACs are highly overlapping. The reconstructions also include 271 On BC axons, coming close to complete coverage of all BCs in a subregion of e2198 roughly (0.1 mm)^2^ in area (Figure 1E). BC axons are much smaller than SAC arbors (Figure 1E). The reconstructions of On SACs and BCs are presented here, while the Off SACs were reconstructed for a previous publication (Kim et al., 2014).

**Figure 2.**
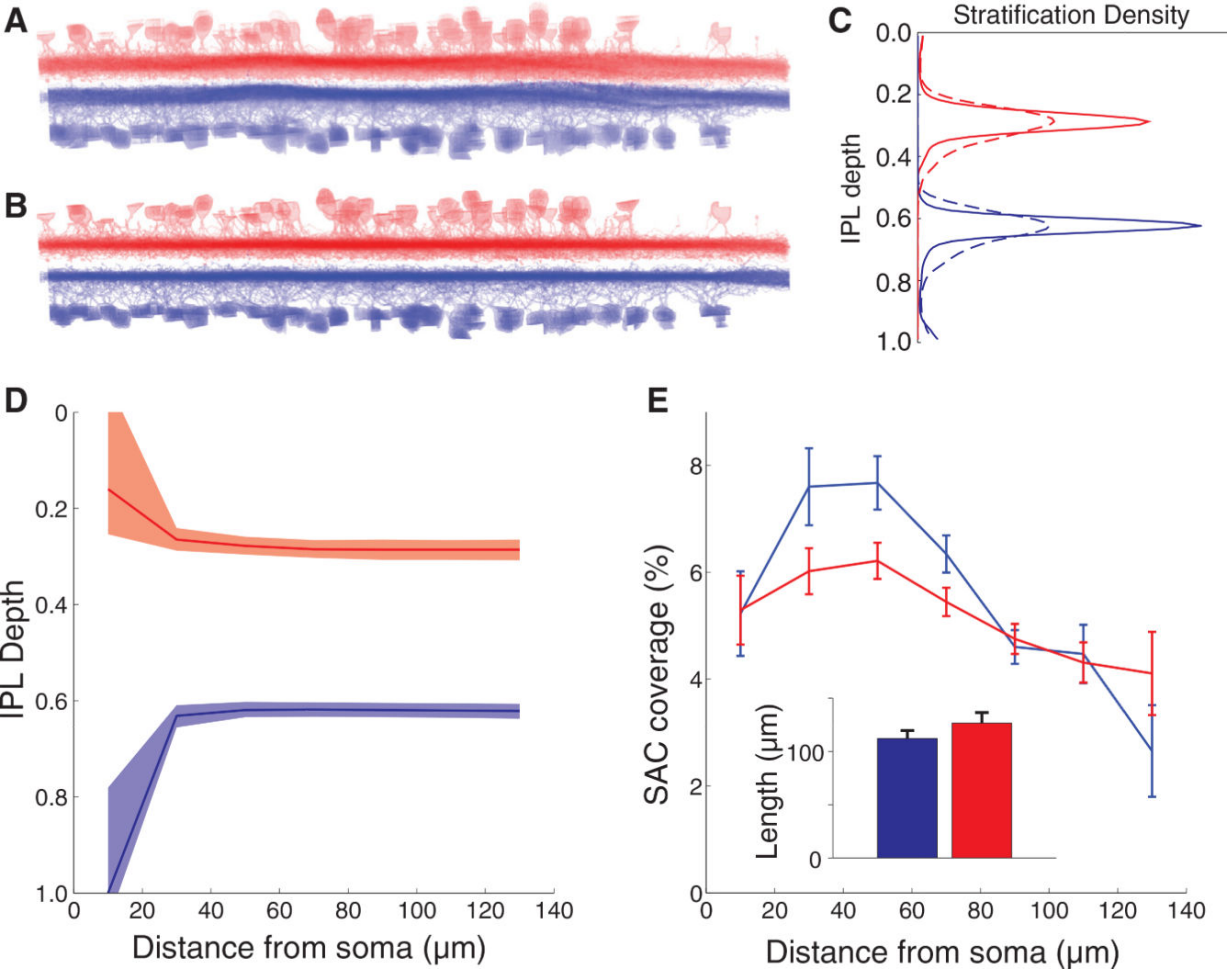
Properties of On and Off SACs, Blue and Red Respectively, and Usage as Landmarks for Quantifying IPL Depth (A) SACs projected along a tangential axis, after rigid alignment to the tangential plane. The curvature and thickness variations of the IPL are evident. (B) SACs projected along a tangential axis, after piecewise bilinear alignment to the tangential plane. (C) SAC stratification profiles before (dashed) and after (solid) piecewise bilinear alignment. (D) SAC stratification depth versus distance from the soma. The lines indicate median and shading the 25^th^ and 75^th^ percentiles. The distance bins are 20 μm wide, beginning with 0 to 20 μm. (E) Fraction of SAC surface in contact with BCs versus distance from the SAC soma. SE is based on the number of BC-SAC pairs at each distance (median length and SD of SAC dendrites, inset).

For classification of retinal cell types, we relied heavily on the stratification profile, defined as the distribution of a cell’s surface area over the depth of the IPL. It is standard to use On and Off SACs as landmarks to define IPL depth. Since the IPL has curvature and variations in thickness (Figure 2A), we computationally flattened the retina by transforming the On and Off SACs into parallel planes (Figure 2B). This type of coordinate transformation improves the accuracy of stratification profiles and has previously been applied in light microscopic anatomy (Manookin et al., 2008; Siegert et al., 2009; Sümbül et al., 2014). Average On and Off SAC stratification profiles became narrower after the transformation (Figure 2C).

Famiglietti (1983) divided SAC dendrites into proximal, intermediate, and distal zones. The median stratification depth of SAC dendrites varies strongly in the proximal zone, weakly in the intermediate zone, and is roughly constant in the distal zone (Figure 2D).

In studies of rabbit retina, Famiglietti (1991) reported that presynaptic boutons are confined almost exclusively to the distal zone, while synaptic inputs from bipolar and amacrine cells are found in all zones. We found that the fraction of On SAC surface in contact with BCs is smaller in the distal zone (Figure 2E), suggesting that BC synapses have some preference for the proximal and intermediate zones of SAC dendrites. We previously reported a similar dependence for Off SACs (Kim et al., 2014); the effect is stronger for On SACs (Figure 2E).

### Subdivision of BC5 into Three Cell Types

Mouse BCs were originally classified into nine cone types (BC1 through BC9) and one rod type based on light microscopic anatomy (Ghosh et al., 2004). Later on, BC3 was subdivided into BC3a and BC3b based on molecular differences (Wässle et al., 2009). Then electron microscopic anatomy was used to distinguish BC3a and BC3b, divide BC5 into two types, and discover an XBC type (Helmstaedter et al., 2013).

**Figure 3.**
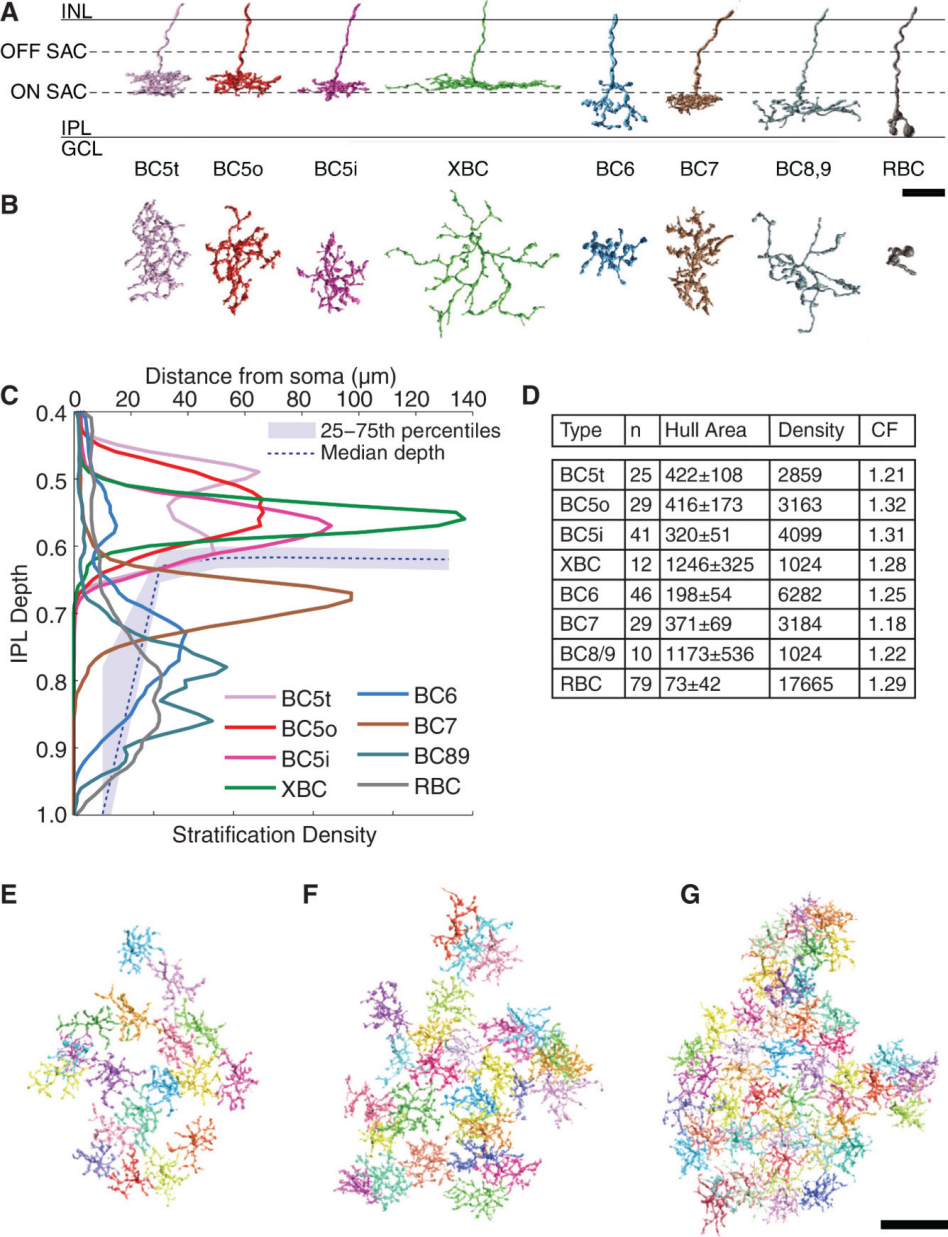
Classification of On BC Types (A and B) Examples of each type, perpendicular (A) and parallel (B) to light axis. (C) Average stratification profiles of types, along with median (dashed line) and quartiles (shaded) of stratification depth of On SAC dendrites versus distance from SAC cell body. (D–G) Table of statistics: number *n* of reconstructed cells; average convex hull area of the projection onto the plane perpendicular to the light axis; estimate of number of cells per *mm*^2^; and coverage factor, sum of convex hull areas divided by area of hull union. The BC5t (E), BC5o (F), and BC5i (G) axonal arbors show few violations of the tiling principle, suggesting that the classification is fairly accurate. The scale bars represent 10 μm for (A) and (B) and 30 μm for (E)–(G).

We revisited the classification of On BCs using the e2198 reconstructions (Experimental Procedures; Figure S1). As shown in the gallery of example cells (Figures 3A and 3B), On BC axons are found in half the depth of the IPL, closer to the GCL than the INL. Our On BC types correspond to those defined previously, with good agreement regarding densities (compare Figure 3D with Figure 1E inset of Helmstaedter et al., 2013). We were able to subdivide BC5 into three types called BC5t, BC5i, and BC5o. The three types stratify at similar depths (Figures 3A and 3C), which is why they were originally grouped into a single Type 5 (Ghosh et al., 2004). Nevertheless, it is possible to differentiate between the types based on subtle differences between their stratification profiles. BC5-inner (“BC5i”) stratifies slightly more toward the inner retina than the other types (Figures 3A, 3C, and S1B). BC5-thick (“BC5t”) is more thickly stratified than BC5-outer (“BC5o”), as shown in Figures 3A, 3C, and S1D. The stratification profile of BC5t is weakly bimodal (Figure 3C), but this property was not used for the classification.

**Figure 4.**
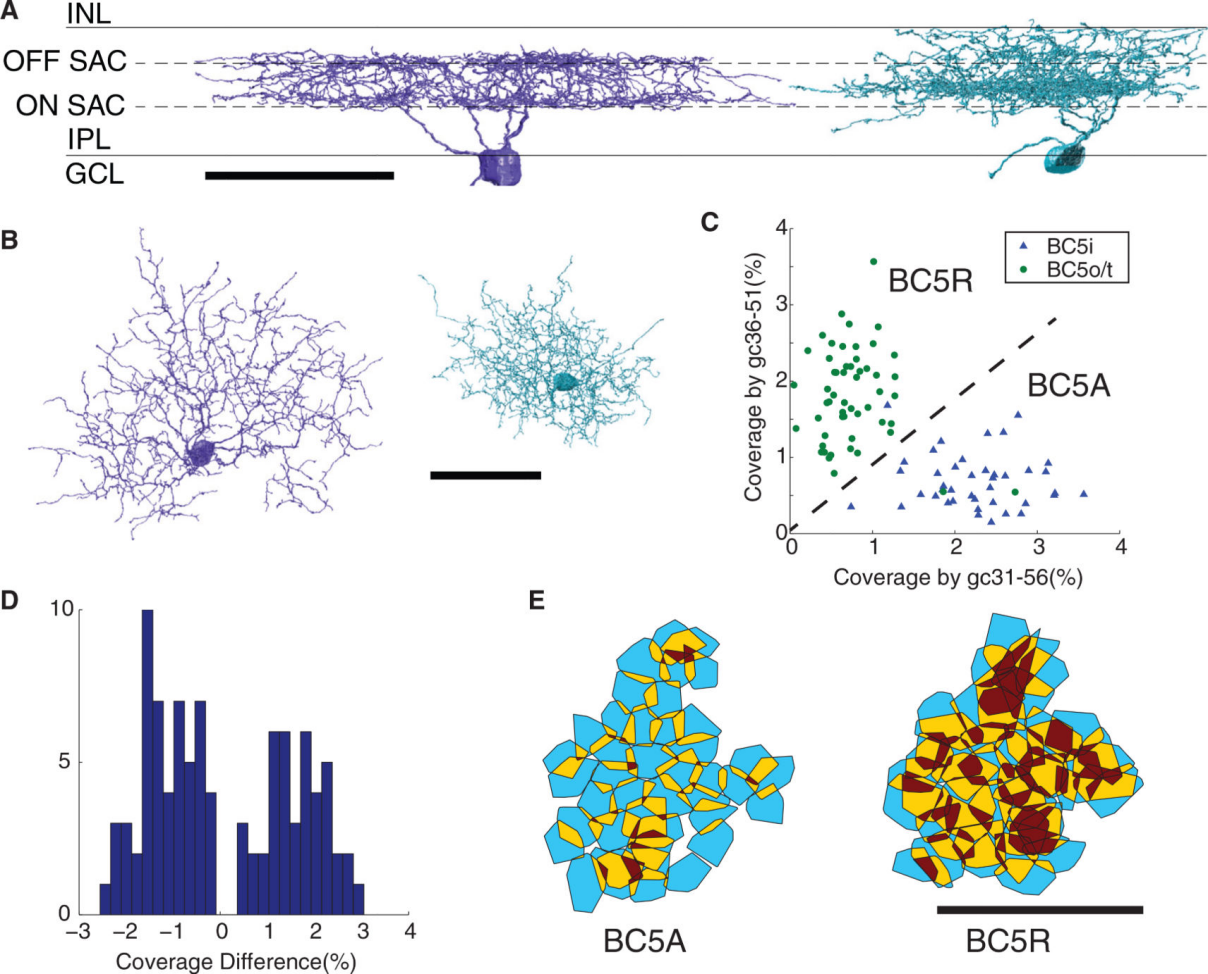
Two-Way Division of BC5 into BC5A and BC5R, Defined by Contact with Ganglion Cell Types (A and B) Representative examples of gc31–56 (blue) and gc36–51 (green), shown perpendicular (A) and parallel (B) to light axis. (C) Each data point represents one BC5 cell, and the coordinates for each cell are the fraction of the BC5 axon in contact with gc36–51 and gc31–56. The dashed diagonal line separates the points into two clusters, named BC5A and BC5R. The color/shape of each symbol indicates whether the cell was classified as BC5i versus BC5t or BC5o based on single cell anatomy and the procedure of Figure S1. BC5A corresponds almost perfectly with BC5i, and BC5R with BC5t/o. (D) Histogram of the same data points as in the previous *xy* plot, but binned by the difference between the *x* and *y* coordinates, i.e., coverage by gc31–56 minus coverage by gc36–51. The histogram separates nicely into BC5R and BC5A clusters. (E) Convex hulls of BC5 cells with two-cell overlap in yellow and three or greater cell overlap in red. The BC5A tiles well, whereas BC5R contains many tiling violations. The scale bar represents 100 μm.

We are confident of our three-way division of BC5 based on stratification (Experimental Procedures; Figure S1), because the axonal arbors of each cluster end up roughly tiling the retina with little overlap (Figures 3E–3G). This “tiling principle” is thought to be a defining characteristic of a true BC type (Wässle et al., 2009). On the other hand, when BC5 cells are divided into just two clusters, it is impossible to avoid many collisions between highly overlapping axonal arbors (Figure 4E).

Only a few violations of the tiling principle are evident in Figures 3E–3G. One possibility is that the tiling principle holds only approximately and that the violations are a form of biological “noise”. Another possibility is that the violations result from errors in our classification procedure. Therefore, we generated a “corrected” classification by swapping a few cells between types (Experimental Procedures). The number of swaps is relatively small (Table S1). The “type gallery” of Figure S3 exhibits our final classification after swapping. The corrected classification was the basis of subsequent analysis of BC-SAC wiring, but our results are qualitatively unchanged even if the uncorrected classification is used.

### Defining BC Types Based on Contact

Only if the stratification profiles of two cells overlap is there potential for contact between the cells and hence potential for synaptic connections. In other words, stratification constrains retinal connectivity (Masland, 2004). It follows that cell types defined using stratification are likely to end up having functional significance, assuming that the connectivity of a cell is closely related to its function (Seung and Sümbül, 2014).

It would be more direct to define a cell type as a set of cells with similar contact or connectivity patterns (Seung, 2009, 2012), rather than use stratification as a proxy for these properties. For example, the 302 neurons of *C. elegans* were divided into 118 classes, each containing neurons with similar connectivity patterns (White et al., 1986). Likewise, Helmstaedter et al. (2013) divided BC5 into classes based on patterns of contact with two ganglion cell types named “gc31–56” and “gc36–51”. We decided to replicate their analysis, mainly in order to determine the correspondence between contact-based and stratification-based classifications. A secondary motivation was to examine how contact-based classification depends on the completeness of reconstruction. Helmstaedter et al. (2013) reconstructed all neurons with cell bodies contained in a (0.1 mm)^2^ patch. This method would not count ganglion cells with arbors inside the patch, but cell bodies outside the patch, evident as gaps in coverage in the gallery of cell types in the Supplemental Data of Helmstaedter et al. (2013). Contact with missing arbors obviously cannot be quantified, hampering contact-based classification.

We were able to replicate and improve the contact-based classification by making use of a large set of ganglion cells that were reconstructed from the e2198 dataset in a parallel study to be reported elsewhere. From this set of ganglion cells, we identified 16 examples of gc31–56 and 19 examples of gc36–51 based on their distinctive stratification profiles (Figures 4A and 4B). The arbors of each ganglion cell type completely cover the central region of e2198 where the BCs are located, because the reconstructed ganglion cells include those with cell bodies outside the central region (Figure S3). For each BC5 cell, we quantified the fraction of its axonal surface area in contact with gc31–56 cells and the fraction of its axonal area in contact with gc36–51 cells. Then BC5 indeed splits nicely into two clusters based on the two contact fractions (Figure 4C). One cluster called BC5A (Helmstaedter et al., 2013), has more contact with gc31–56. BC5A tiles with few violations (Figure 4E) and therefore appears to be a pure cell type. BC5A corresponds almost exactly with BC5i (Figure 4C).

The other cluster, named “BC5R” by Helmstaedter et al. (2013), has more contact with gc36–51. Because BC5R contains many tiling violations (Figure 4E), Helmstaedter et al. (2013) speculated that BC5R was a mixture of more than one type. Our stratification-based classification confirms their speculation by effectively dividing BC5R into BC5o and BC5t, both of which tile the retina separately.

The cleanness of the division between BC5A and BC5R is evident in a histogram of the difference between the gc31–56 and gc36–51 contact fractions, in which two well-separated clusters are evident (Figure 4D). Note that Helmstaedter et al. (2013) labeled some of their reconstructed cells as BC5X. This name was not intended to be a type, but rather indicated cells that were unclassifiable because they lacked contact with gc31–56 and gc36–51. We do not have this problem because our coverage of the ganglion cell types is more complete.

### BC-SAC Contact Analysis

**Figure 5.**
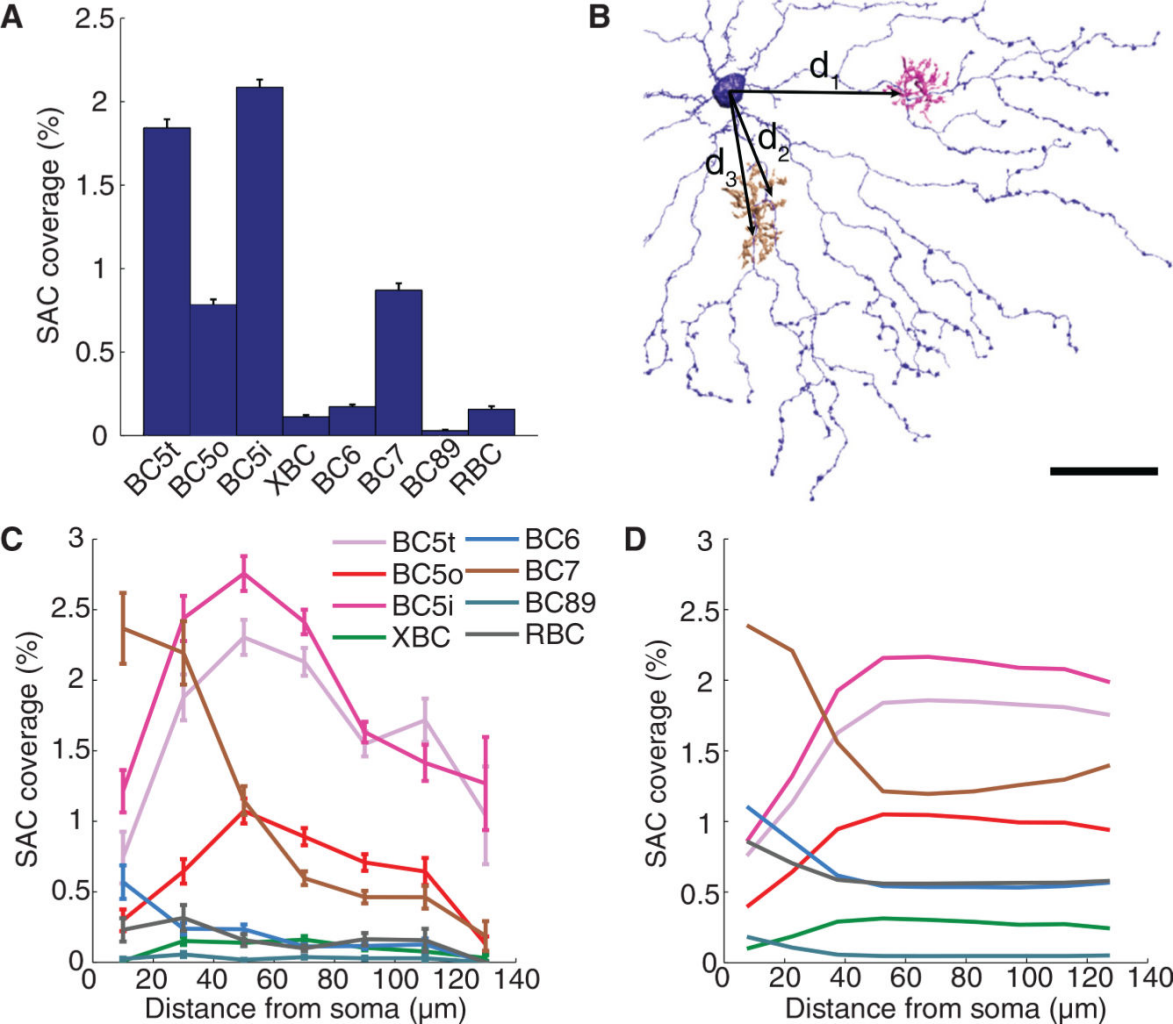
Wiring Specificity of On BC-SAC Circuit (A) Fraction of On SAC surface area contacted by On BC types. The error bars indicate SE. (B) Distance is defined for each BC-SAC pair in the tangential plane from the centroid of BC-SAC contact to the point on the SAC soma from which the dendritic trunk emerges. The centroid of emergence points is used if there are multiple trunks. (C) Fraction of On SAC surface contacted by On BC types versus distance from the SAC soma. SE is based on the number of pairs for each BC type and distance. (D) Contact predicted from co-stratification analysis. The scale bar represents 30 μm.

We computed contact area between SACs and BCs of each type. The absolute areas were normalized to produce an estimate of the percentage of SAC surface area covered by BCs of each type (Experimental Procedures). This contact analysis suggests that BC5t, BC5i, and BC7 are likely the dominant BC inputs to the On SAC (Figures 5A and 5C). Our result is consistent with previous anatomical reconstructions (Helmstaedter et al., 2013) and physiological experiments (Duan et al., 2014; Chen et al., 2014), though these previous studies did not distinguish all three BC5 types. If there is BC5o input, it is likely substantially weaker than BC5t and BC5i input (Figure 5A).

To characterize the spatial relations between contacting cells, we examined the dependence of BC-SAC contact on distance between the BC axon and the SAC soma (Figure 5B). The absolute areas were normalized to estimate the percentage of SAC surface area covered by BCs of a given type at a particular distance from the SAC soma (Experimental Procedures). The resulting graphs show that BC7 prefers to contact On SAC dendrites near the SAC soma, whereas BC5t and BC5i prefer to contact at an intermediate distance from the soma (Figure 5C).

One might worry that our contact analyses are sensitive to incomplete reconstruction of BCs (see Experimental Procedures and “holes” in the tilings of Figures 3E–3G). To avoid this problem, the results of our analyses are expressed not as absolute contact areas, but instead as fractions of SAC surface area. To demonstrate robustness, we repeated our contact analyses after deleting a random subset of BC5t and BC5i cells (Figures S2A–S2C). The estimates of SAC coverage turned out virtually unchanged (Figures S2D and S2E); SAC coverage by BC5o was still much lower than by BC5t and BC5i.

### BC-SAC Co-stratification Analysis

Figure 2D already showed that proximal SAC dendrites span a wide range of IPL depths, which are different from the depths of the intermediate and distal dendrites. Because of this depth difference, the proximal zone co-stratifies with BC7, but only weakly with BC5, which is consistent with our observed preference of BC7 for contact with the proximal zone. Such reasoning was already used by Famiglietti (1991) to infer that proximal dendrites should receive inputs from different BC types compared to distal dendrites.

This suggests that co-stratification could be used to quantitatively predict On BC-SAC contact using the integral over depth of the product of BC and SAC stratification profiles. Figure 5D shows that this prediction works well in some respects, but not in others. On the one hand, predicted contact (Figure 5D) nicely matches actual contact (Figure 5C) for BC5t, BC5i, and BC7, failing only to match the observed decrease in the distal zone. On the other hand, actual contact of BC6 is much lower than expected from predicted contact.

It may seem surprising that BC6 makes little contact with On SACs, given that it stratifies over a broad range of IPL depths that includes all zones of On SAC dendrites (Figure 3A). One reason may be that the BC6 stratification profile dips down exactly at the depth of intermediate and distal dendrites (Figure 3C), as if BC6 were trying to avoid contacting the On SAC.

## DISCUSSION

Our reconstruction of the On BC-SAC circuit suggests that its wiring diagram parallels that of the Off BC-SAC circuit (Figure 1C). We find that sustained BC types prefer to contact SAC dendrites near the SAC soma and transient BC types prefer an intermediate distance from the SAC soma. We interpret these contact preferences as reflecting synaptic connectivity preferences.

The spatial organization of the IPL has previously been interpreted as supporting rules of wiring specificity. For example, the division of the IPL into On and Off sublayers (Figure 1A) supports On to On and Off to Off rules for wiring of BCs to ganglion cells (GCs) (Famiglietti and Kolb, 1976; Pang et al., 2003). Similarly, the division of the IPL into sustained and transient sublayers (Figure 1B) could support sustained to sustained and transient to transient rules of BC-GC wiring (Awatramani and Slaughter, 2000). Here, the BC-SAC wiring diagram provides an explanation of why On and Off SACs are located at the boundaries between sustained and transient IPL sublayers (Figure 1B). Namely, this positioning is appropriate for receiving convergent input from both sustained and transient BC types (Figure 1C).

We also find three differences between the On and Off circuits. First, BC7 and BC5i/t prefer to contact closer to the On SAC soma than their analogs BC2 and BC3a contact to the Off SAC soma (compare Figure 5C with Figure 4d of Kim et al., 2014). Second, the On SAC is contacted strongly by two transient BC types, while the Off SAC receives strong contact from a single transient BC type. Third, BC contact on distal SAC dendrites is relatively scarcer for the On than the Off circuit (Figure 2E).

The current study comes with several caveats. First, while most of the available evidence supports the sustained-transient classification of On BC types adopted in this paper (Baden et al., 2013; Borghuis et al., 2013), the literature contains at least one divergent report about this classification (Ichinose et al., 2014). Second, synaptic connectivity cannot be identified with certainty in e2198, because of an unconventional staining technique that left intracellular organelles invisible. Therefore, we rely on contact between cells as an indirect indicator of connectivity. Third, motion computation by SAC dendrites might involve biophysics of SAC dendrites (Gavrikov et al., 2003; Tukker et al., 2004; Hausselt et al., 2007), which is not incompatible with our hypothetical mechanism involving space-time wiring specificity.

We were able to divide BC5 into three types (BC5t, BC5o, and BC5i), based on differences in stratification. Using patterns of contact with two GC types, we also replicated the prior two-way division by Helmstaedter et al. (2013) of BC5 into BC5A and BC5R and found that BC5A corresponds almost perfectly with BC5i. We were able to assign all BC5 cells to either BC5A or BC5R, because our reconstructions of the GC types are more complete than those of Helmstaedter et al. (2013). In the future, we expect contact or connectivity to replace stratification as the main property used to classify cells into types (Seung, 2009, 2012; Jonas and Kording, 2015).

Wässle et al. (2009) defined BC5 with a 5-HT3R-EGFP transgenic mouse line. They speculated that the line labeled two BC5 types, because of the high density of labeled cells and because two BC5 types had been molecularly distinguished in rat (Fyk-Kolodziej and Pourcho, 2007). However, they were unable to find molecular markers distinguishing BC5 types in mouse. Duan et al. (2014) showed that Kcng4 and Cdh9 Cre lines label the same cells as 5-HT3R-EGFP. Haverkamp et al. (2009) found that the BC5 cells in the 5-HT3R-EGFP line were all CaBP5-positive. BC5t may be CaBP5-negative (Haruhisa Okawa and Rachel Wong, personal communication). If this is the case, it follows that BC5o and BC5i correspond to the two types in the 5-HT3R-EGFP transgenic line.

The catalog of mouse BC types is likely complete. Our claim is based on two assumptions: (1) every BC type tiles the retina with little overlap, and (2) there are no large BCs, which would be rare and therefore could have been missed by our reconstructions and those of Helmstaedter et al. (2013). One anomaly is that BC1 and BC2 tilings exhibit more overlap than those of other types (Extended Data Figure 6 of Kim et al., 2014). The overlap is not enough to allow defining a third type that fully tiles the retina; a hypothetical third type would be sparse in its coverage. Another qualification is that our reconstructions come from a single location in a retina, so we cannot exclude the possibility that cell types vary across the retina.

## EXPERIMENTAL PROCEDURES

The methods of the present study are similar to those used previously (Kim et al., 2014), so the differences are the focus of the following text. As in the previous study, all dimensions are uncorrected for shrinkage, which was previously estimated at 14% by comparison of two-photon and serial electron microscopy images (Helmstaedter et al., 2013).

### Alignment to a Common Coordinate System

In our previous study of the Off BC-SAC circuit (Kim et al., 2014), we defined normalized coordinates that computationally flattened the Off SACs. In this study, we improved the coordinate system by additionally utilizing On SACs as landmarks.

The volume was first rigidly transformed to minimize the averaged squared distance of Off SACs to the *xy* plane. A rectangular 32 × 36 lattice was defined on the *xy* plane, with nodes spaced at approximately 10 μm intervals. For each lattice node, we computed the mean depth of all Off SAC surface voxels and the mean depth of all On SAC surface voxels within a cylindrical neighborhood. Bilinear interpolation of these depths yielded values $\mu_{x,y}^{OFF}$ and $\mu_{x,y}^{ON}$ for every point in the *xy* plane. Then the depth *z* of every point (*x,y,z*) was transformed according to $z\prime =(z-\mu_{x,y}^{OFF})(\mu_{x,y}^{ON}-\mu_{x,y}^{OFF})$, yielding a coordinate system in which Off and On SACs are at depths 0 and 1, respectively. Finally, we linearly transformed to coordinates in which Off and On SACs are at 0.28 and 0.62 IPL depth, respectively, for consistency with the definitions of Helmstaedter et al. (2013).

### SAC Reconstruction

On SACs were reconstructed mostly during July 2013 to September 2014. EyeWirers who helped reconstruct On SACs are listed in the Supplemental Information.

Off SACs were previously reconstructed both by forward tracing from the candidate SAC soma to dendritic tips and backward tracing from varicosities on candidate SAC dendrites to the soma (Kim et al., 2014). The forward method turned out to be less useful for On SACs, because their dendrites can take rather circuitous paths before reaching their final IPL depth and making the distinctive starburst shape. Therefore, it takes a great deal of reconstruction effort before a candidate cell can be accepted or rejected as a SAC. It saves human effort if this decision can be made earlier in the reconstruction process. There were two On SACs that were reconstructed by lab workers using the forward method. The remaining 75 were reconstructed by EyeWirers using the backward method.

In a parallel study to be reported elsewhere, we exhaustively reconstructed all neurons with somata in a (200 μm)^2^ GCL patch of e2198. This revealed six extra On SACs beyond the 35 in the patch that had already been reconstructed for the present study. In other words, the reconstructions of the present study had achieved 85% coverage of all On SACs in this (200 μm)^2^ patch. Assuming that the density of On SACs is the same for all of e2198 as it is in the (200 μm)^2^ patch, the estimated number of On SACs in e2198 is roughly 110 and our overall coverage is roughly 70%. Our estimated coverage of Off SACs is slightly lower, as the Off SAC density is known to be slightly (less than 10%) larger than the On SAC density (Jeon et al., 1998).

### SAC Properties

Length of SAC dendrites (Figure 2E, inset) was calculated as the mean distance from the soma on the *xy* plane of the eight most distant points that are not within 30 μm of each other. These parameters were chosen because the points generated appeared to give an accurate representation of the dendritic length, while avoiding inaccuracy that arises from outliers and from dendrites that extend beyond the bounds of the volume.

### BC Reconstruction

On BCs were reconstructed mostly during February to December 2014. Because e2198 extends only partially into the INL, it was not possible to identify BCs based on the existence of a dendritic arbor in the OPL. Instead, we identified BC axonal arbors by comparison with Helmstaedter et al. (2013), who reconstructed all BCs in a patch of retina that included both IPL and OPL. BC axon candidates were neurites that pass through the interstices of the INL and emerge in the IPL. Many candidates could be immediately rejected as amacrine or GCs because their arbors were too large or rejected as glial cells based on surface concavity and roughness. Little human effort was necessary for these cases, because large parts of these cells were automatically reconstructed. The remaining candidates were put into the reconstruction pipeline and were rejected as narrow field amacrine cells if their stratification profiles deviated markedly from those previously reported by Helmstaedter et al. (2013) for BCs. There were eight that were rejected in the middle of reconstruction, and three that were rejected after full reconstruction.

### BC Classification

We define the stratification profile as the density of surface area versus depth in the IPL. For the purpose of BC classification, we restricted the domain of the stratification profile to the interval between IPL depth 0.4 and 1. The domain omitted depths between 0 and 0.4 to exclude the trunks of the axonal arbors, which increase variability of the stratification profiles. Each stratification profile is normalized like a probability density, so that profile area between IPL depths 0.4 and 1 integrates to unity. Since IPL depth is dimensionless, the stratification profile is also dimensionless. Helmstaedter et al. (2013) defined stratification profile as the density of reconstructed skeleton. This definition is slightly different from ours, but yields similar results (data not shown). Percentiles are defined for a stratification profile in the same way as for a probability density. Namely, the interval from the *n*^th^ percentile depth to 0^th^ percentile depth contains *n* percent of the area of the stratification profile. As mentioned earlier, 0^th^ percentile depth is defined as IPL depth 0.4. The thickness of the stratification profile is defined as the difference between 85^th^ and 25^th^ percentile depths. Helmstaedter et al. (2013) defined thickness as the difference between 75^th^ and 25^th^ percentile depths, which yields similar classifications. In addition to stratification, we characterized single cell anatomy by a further property, the area of the cell’s projection onto the tangential (*xy*) plane.

We hierarchically clustered our On BCs as follows. The axonal arbors of BC5 and XBC lie between the Off and On SACs. Accordingly, a BC5/XBC cluster separates from other types based on 85^th^ percentile depth (Figure S1A). This cluster in turn subdivides by 25^th^ percentile depth into outer (closer to the INL) and inner (closer to the GCL) clusters (Figure S1B). The outer cluster can be divided into BC5t and BC5o; the former is more thickly stratified than the latter (Figure S1D). The inner cluster divides into XBC and BC5i based on projection area (Figure S1E).

Types outside the BC5/XBC cluster lie between the On SACs and GCL. BC7 separates from the others by 85^th^ percentile depth (Figure S1A). Then BC6, BC8/9, and RBC separate from each other based on projection area (Figure S1C). We chose not to separate BC8 and BC9, as the reconstructed cells were too few to yield two complete tilings.

BC8, BC9, and RBC all appear underrepresented relative to Helmstaedter et al. (2013). This discrepancy could be artifactual, caused by failures to identify the relatively thin axons of types BC8 and BC9 in the interstices of the INL. Alternatively, these cell types might be truly underrepresented in our volume.

Histograms showing the various splits in the hierarchical clustering are shown in Figure S1. The splits are highly convincing near the top of the hierarchy, but less convincing near the bottom. Therefore, we sought further validation from the principle that the arbors of a BC type should “tile” the retina with little overlap. If the hierarchical clustering yields cell types that tile the retina, that would be independent validation of the clustering, which relied only on anatomical properties of single cells. For all types, few violations of the tiling principle were observed (Figures 3E–3G). There are some holes in the tilings, but they are likely the result of omissions in cell reconstruction rather than classification errors. Violations of tiling can be rectified by swapping cells between types, to yield the improved classifications given in Figure S3. The fraction of swapped cells is small (Table S1). Our final classification is exhibited in the type gallery of Figure S3.

### BC-SAC Contact Analysis

We only reconstructed BCs in the central area of e2198 (Figure 1E) and even within this area some BCs may have been missed (Figures 3E–3G). In our previous paper, we described methods of analyzing BC-SAC contact that are robust to both kinds of incompleteness (Kim et al., 2014). The same methods were applied here, with only minor changes.

We compute each SAC’s total contact area with all BCs of a given type divided by the SAC’s surface area contained in the convex hull of the same BCs. This yields an estimate of the fraction of the SAC’s surface in contact with BCs of the given type. This fraction is averaged over SACs to yield the estimates shown in Figure 5A, with SE based on the number of SACs that intersect the convex hull of the given BC type.

For each BC type, a coverage factor is computed by dividing the sum of hull areas for cells of the given type by the area of the union of hulls of the cells. The coverage factor represents the extent to which neighboring BCs of the same type overlap one another.

For each BC-SAC pair, we compute the contact area divided by the surface area of the SAC within the convex hull of the BC. Multiplying by the coverage factor for the BC type yields an estimate of the fraction of the SAC’s surface area contacting the BC type at that distance from that SAC’s soma. This computation is done for all BC-SAC pairs, except that we discard pairs for which the BC hull contains fewer than 500 SAC surface voxels in order to dampen fluctuations. We bin the remaining BC-SAC pairs by distance and by BC type. For each bin and for each SAC that contributes pairs to that bin, we compute the mean over BC-SAC pairs. Each data point in Figure 5C represents the mean of the SAC-specific means for that bin and SE is based on the number of SACs that contribute to that bin.

Figure S2 demonstrates the robustness of our analysis by showing that estimated SAC contact fractions change little even after randomly deleting many BCs.

The contact analysis was done with BC types given by hierarchical clustering after a small number of swaps to correct for tiling violations, as explained above and in Table S1. The results of the contact analysis look similar if the tiling swaps are not performed (data not shown).

## Supplementary Material

1

2

## Highlights

- We analyzed serial electron microscopic images of a mouse retina
- On starburst amacrine cells (SACs) and bipolar cells (BCs) were reconstructed
- We defined an additional On BC type in the course of classifying On BCs
- Sustained and transient BC types are wired to SAC dendrites at different distances
