# Supplementary material for: Analogous Convergence of Sustained and Transient Inputs in Parallel On and Off Pathways for Retinal Motion Computation
Source: Cell Rep. Author manuscript; Available in PMC 2019 Mar 7. (PMC6404534; doi:10.1016/j.celrep.2016.02.001)
Supplement: 1 [file NIHMS757638-supplement-1.pdf]

Cell Reports, Volume *14*

Supplemental Information

Analogous Convergence of Sustained and Transient Inputs in Parallel On and Off Pathways for Retinal Motion Computation

Matthew J. Greene, Jinseop S. Kim, H. Sebastian Seung, and the EyeWirers

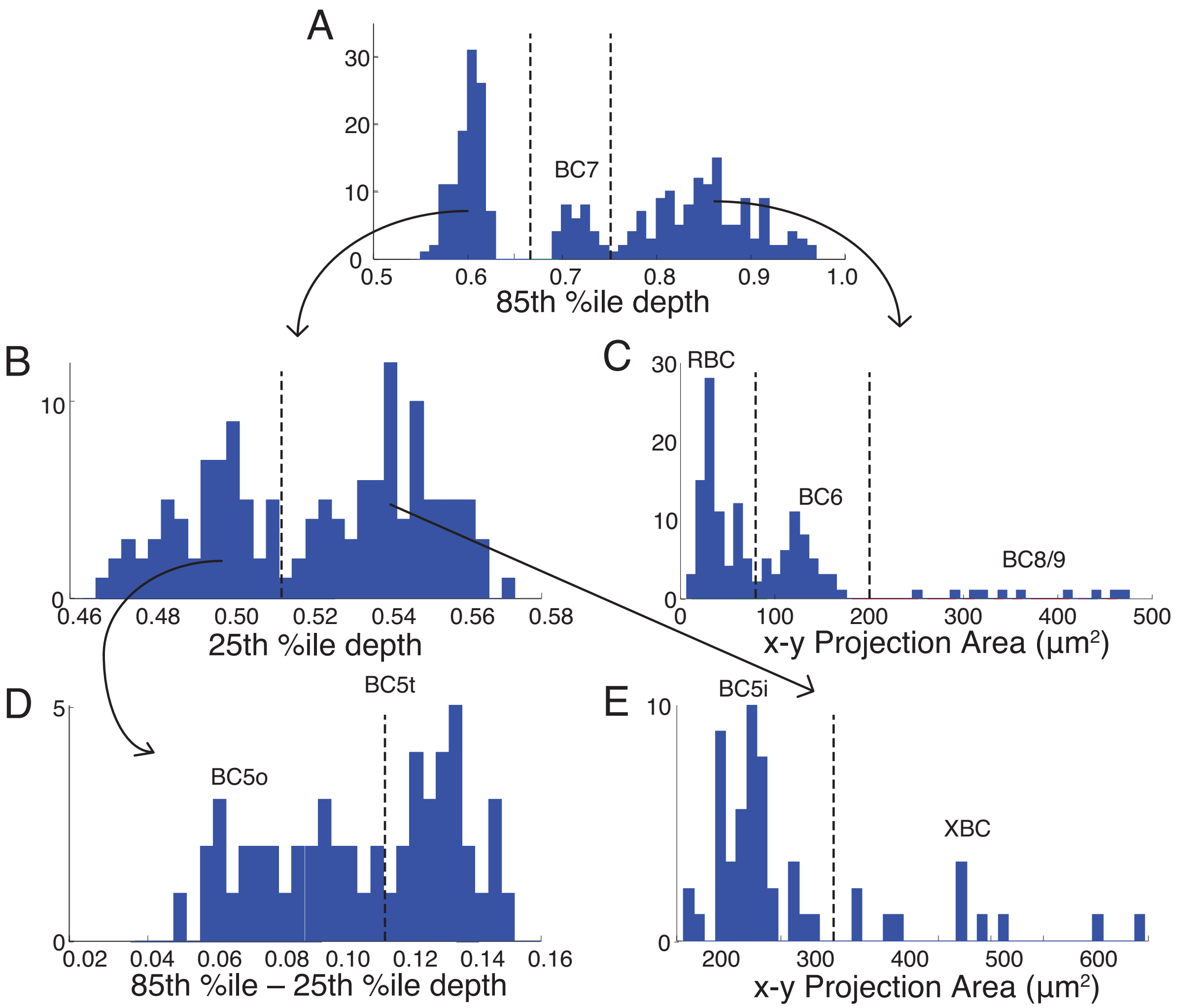
A
B
C
D
E
BC7
RBC
BC6
BC8/9
BC5t
BC5o
BC5i
XBC
85th %ile depth
25th %ile depth
x-y Projection Area (μm2)
85th %ile – 25th %ile depth
x-y Projection Area (μm2)

Figure S1

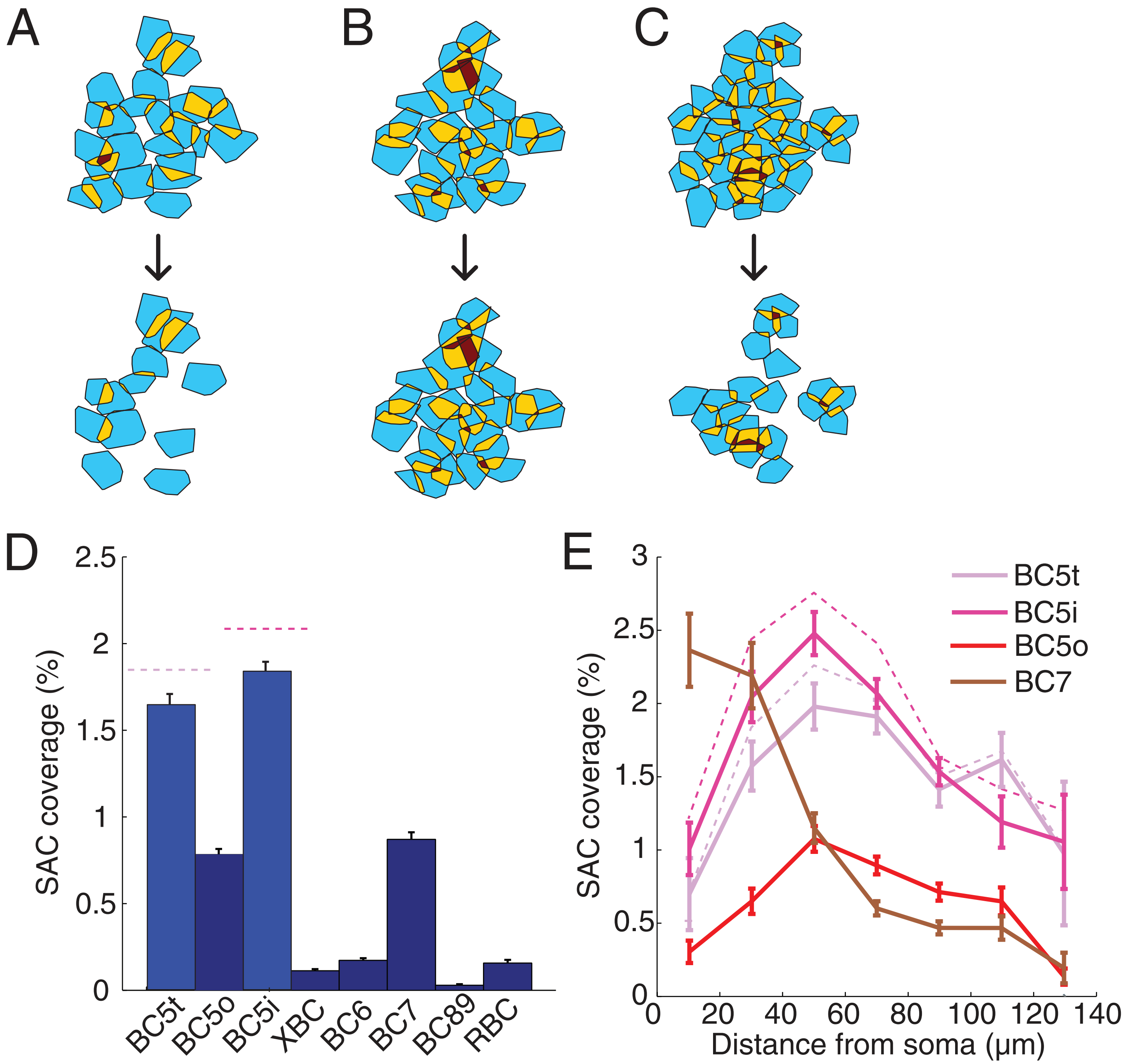
A
B
C
D
2.5
2
1.5
1
0.5
0
SAC coverage (%)
BC5t
BC5o
BC5i
XBC
BC6
BC7
BC89
RBC
E
3
2.5
2
1.5
1
0.5
0
SAC coverage (%)
0
20
40
60
80
100
120
140
Distance from soma (μm)
BC5t
BC5i
BC5o
BC7

Figure S2

Supplemental Figures

1. Figure S1. Refers to Experimental Procedures: BC classification. Hierarchical clustering procedure for On BCs. The procedure is based primarily on percentiles of the stratification profile. Dashed lines show locations of dividing points. (A) BCs are initially subdivided into three groups by 85th percentile IPL depth. (B) BC5 types and XBC are further divided by 25th percentile IPL depth. (C) RBC, BC6 and BC8/9 are distinguished by the total area of their projection onto the *xy* plane. Due to the sparse representation of BC8/9 in our set of reconstructed cells, we were unable to subdivide it. (D) BC5t and BC5o are differentiated by stratification thickness, as defined by the difference between a cell's 85th and 25th percentile IPL depths. (E) BC5i and XBC differ in the total area of their projection onto the *xy* plane.

2. Figure S2. Refers to Experimental Procedures: BC-SAC contact analysis. Robustness of to missing bipolar cells. Approximately half of BC5i (A) and BC5t (C) cells were randomly deleted, while BC5o was left unchanged (B). The BC5i and BC5t tilings are obviously incomplete, and BC5o now has the most complete coverage. (D, E) The results of contact analysis (solid lines) are only slightly shifted relative to the original contact analysis of Figs. 4A and 4C (dashed lines). BC5o still contributes much less than BC5i and BC5t to SAC coverage, even though BC5o cells are now the most numerous.

		Initial Classification							
		BC5t	BC5o	BC5i	XBC	BC6	BC7	BC8/9	RBC
Final Classification	BC5t	24	4						1
	BC5o	1	18						
	BC5i		7	41	3				
	XBC				9				
	BC6					39			3
	BC7					3	29		1
	BC8/9							10	
	RBC					4			74

Table S1

Supplemental Table

1. Table S1. Refers to Experimental Precedures: BC classification. Number of corrections required to resolve tiling violations. Types as defined by single cell anatomy through the procedure described in Fig. S1 yielded an initial classification that contained some violations of the tiling principle (Figs. 3E, F, G). To produce the final classification shown in Figs. S2 and S5, tiling violations were manually corrected by swapping cells to different types with similar stratification profiles or other single cell anatomical properties. The off-diagonal elements of the matrix quantify the number of manual corrections required. The number of corrections is small relative to the total number of cells.

Supplemental Dataset

1. Dataset S1. Refers to Figure 4 and Experimental Procedures. Gallery of cell types referred to in the main text. On each page: (Top-Left) *xz* and *yz* orthogonal projections of skeletonized cells. Dashed lines indicate the IPL borders; dotted lines indicate the location of the Off and On SACs. Individual cells are randomly colored. (Bottom-Left-Left) *xy* projection of skeletonized. (Bottom-Left-Right) Convex hulls of cells, projected onto the *xy* plane. The region covered by exactly one convex hull is colored blue, the overlap of two hulls is colored orange, and the overlap of three or more hulls is colored red. (Top-Right-Left) Stratification profiles of individual cells and their mean. Colors are chosen as those in the panels to the left, while the mean stratification profile is overlaid in black. (Top-Right-Right) Representation of the projected area and stratification thickness of individual cells. On the horizontal axis, each line, colored as in the skeleton views, begins at the point of the cell's 25th percentile depth and ends at its 75th percentile depth. The height of each line on the vertical axis indicates the cell's projected area in μm^2. (Bottom-Right-Top) Contact of the cell type with other cell types, as a percentage of the surface area of the type specified on the horizontal axis. (Bottom-Right-Bottom) Contact of the cell type with other cell types, as a percentage of the cell type's surface area. Scale bars, 50 μm.

Supplemental Notes

The following EyeWirers took on leadership roles during the reconstruction of the On SACs:

- Mentors helped novice players to integrate into the community and improve their tracing skills: michellewooten, mamateresa, galarun, aesanta1, Nseraf, rinda, damocles357, sambob496, jax123, IcyCuber, addieye, susi, LotteryDiscountz, kronnn, Emeraldstar, ronin.
- Moderators helped mobilize the community and intervened in case of conflict between players: lindsay, galarun, jinbean, rinda, a5hm0r, susi, sarabc, tek50, Nseraf, kronnn, marhav, addieye, lilmoo, michellewooten, mamateresa, ronin, b_hailey, jax123, jungmanp88, LD2, LotteryDiscountz, angler.korea, darwinkim, aesanta1, pkeoughan, minsuyang, MaraTara, ttore63, KateLee, northingood.
- Scouts reported possible reconstruction errors: susi, reb1618, ketta, jinbean, iridium, a5hm0r, rinda, azoarcus, tek50, tiikerikani, marhav, galarun, iam1105, grizle, kanaborama, hecticryryry, viksta, designdruid, Spicythunder, bigredjeep007, lilmoo, mamateresa, ais, kshearer, addieye, Nseraf, Flos, kronnn, dataminerstarr, bachmann, BladesRUS, jax123, retepaskab, ArcanzaJenkins, ronin, shannonlevine, b_hailey, EllenRipley, uzenik, MaraTara, cdaywalt66, clarrisani, Pepich, eldendaf, mcfly, mousehouse, aesanta1, damocles357, sdunn61781, lobusparietalis, Emeraldstar, jamiexq, blackblues, frabuleuse, CreeperInfinity, faunhaert, mmcdermott55, amandak, doge, michellewooten, sambob496, LotteryDiscountz, PanDan, LD2, chrisrolik, EinsteintheRapper, jbinsc, IcyCuber, Kekelktus, theo1977, aldof, wlindboe, nopasaran, BlackCat13, Cerebus, Andrearwen, 1sigor, m7md, masterweaver, whitefieldcat.
- Scythes helped correct reconstruction errors: marhav, a5hm0r, jinbean, ketta, reb1618, rinda, tek50, tiikerikani, galarun, grizle, iridium, susi, bigredjeep007, addieye, kshearer, lilmoo, Nseraf, mcfly, BladesRUS, kronnn, dhartiste, b_hailey, ronin, MaraTara, jungmanp00, kradul2, doge, aesanta1, mamateresa, lemongrab, Slide20xl, LotteryDiscountz, michellewooten, blackblues, sambob496, jax123, damocles357.

The 1,438 EyeWirers who contributed to reconstructing On SAC dendrites are (along with number of gameplay cubes): Nseraf (74,340), twister2 (53,307), a5hm0r (40,008), galarun (25,734), aldof (23,748), susi (20,980), iridium (18,790), MaraTara (17,131), aesanta1 (16,709), benficus (16,034), jinbean (14,530), sdunn61781 (14,312), jamiexq (14,060), mamateresa (13,919), jbinsc (13,890), Emeraldstar (13,680), lobusparietalis (11,398), sambob496 (10,378), damocles357 (10,107), sneakybaron (10,079), rinda (9,793), tiikerikani (9,685), faunhaert (9,425), Begonie (8,952), LotteryDiscountz (8,222), m7md (8,126), DannyS (8,019), charleskoch (7,843), ronin (7,812), grizle (7,721), jcostantino (7,667), tek50 (7,574), crazyman4865 (7,217), michellewooten (7,187), pkeoughan (6,793), IcyCuber (6,596), 1sigor (6,011), jax123 (5,804), lilmoo (5,704), marhav (5,491), dereksims (5,330), b_hailey (5,327), lynnschlos (5,208), Cerebus (5,115), mmcdermott55 (4,959), mardim2 (4,921), blackblues (4,579), Chainsaw_NL (4,485), ic167 (3,977), sappy (3,848), dataminerstarr (3,829), carpwoman (3,604), HariSeldon (3,594), pistachio (3,552), Enma (3,542), jesmith_nm (3,274), ericafulness (3,224), wee (3,149), psimka (3,123), martinj (2,856), lopezlv (2,777), ginny (2,773), sjc2153 (2,695), Andrearwen (2,582), retepaskab (2,552), JacquesG (2,483), dougrike (2,446), Kekelktus (2,430), peggyu (2,394), lotus98 (2,387), addieye (2,387), bdesmartis (2,371), toknow (2,351), greglanders (2,294), PhineasB (2,261), uzenik (2,260), tryffyd (2,192), wlindboe (2,120), erinys (2,050), CreeperInfinity (2,046), BlackCat13 (2,014), wolfryder101 (1,937), mollica (1,865), keshlam2 (1,797), inockach (1,734), graigrai (1,705), eldendaf (1,688), mywire (1,677), rutho13 (1,661), gruigah (1,655), jabsco (1,650), dragonturtle (1,612), lcurtisadams (1,556), nspainter77 (1,555), chrisrolik (1,552), nkem (1,527), sherryhamm (1,524), ssaba (1,519), mstanley (1,519), mileslane (1,515), shannonlevine (1,514), EllenRipley (1,506), Passiflora (1,497), eyegap (1,478), weizhe1983 (1,455), panosdalk (1,444), herty (1,432), mbb480 (1,428), stitcher52 (1,393), jsileo3 (1,352), Hannibal87 (1,333), TheStatPow (1,329), kloez (1,326), lringelstetter (1,318), dejavu1031 (1,309), ChelonianRiot (1,294), reb1618 (1,292), Quinlan. (1,292), wojtekp (1,280), aise (1,278), ouiz (1,270), mhelm (1,262), nopasaran (1,252), pycospain (1,244), Pif (1,208), mrmathi (1,198), dhill23 (1,194), bigbiff (1,185), ppotter613 (1,170), Marta_M (1,163), otto (1,157), toocajun (1,113), frabuleuse (1,107), Lauri22 (1,058), lili77 (1,057), kronnn (1,048), FractalCuber (1,043), buco (1,039), sappygoblin (1,020), schmelik (1,017), alswns4097 (1,014), FluffySab (1,011), whitefieldcat (995), lukata (969), walty (964), gartral (964), vague_nomen (963), myrklv (963), judystarbuck (951), Lgchinadragon (950), ultrapeanut (948), ArcanzaJenkins (943), Maejoh (939), adrian_alexis (939), Varppi (936), borrowedbluebox (926), newcity9141 (925), sumo (921), rogis1 (921), lyzzard (918), batchen (917), cadrake (885), kab (883), mainbrain (877), jyri (862), anitram (856), clarrisani (851), Dandy (838), soniac (822), bigredjeep007 (822), laxcav (821), dbhunter (818), gebe (817), kshearer (795), ssef0120 (790), mam711 (779), amyd508 (779), elentari (773), Sujuperstar71 (763), sliet (756), bretfrd

(754), siese (753), jungmanp88 (747), pandasecond (726), garret85 (726), 000 (726), theo1977 (725), dejerpha (721), pishe (720), oc3711 (720), ren53nyc (702), jdoncarlos (694), besttry (691), co1ored (687), Mariahkitten (681), jh1109 (681), syllogic (680), skeeter_mcbee (680), bemaline (678), suburbanexile (669), Iliyana (669), marcus737 (663), catter (659), LaurieMarks (657), Laurcifer (654), klimp (649), mmweiland (645), annmonty (644), mjyothik (641), Rosse (634), marika (634), Cat4248 (622), tunerk7 (611), jonas.j.nordin (604), MissLiv (602), aebarnett1 (601), iconforhire (596), ohleyer (591), zuotian (585), aluorvats (582), svincent (580), asd123 (580), dryczko (574), cooljohnny3 (568), blabbermouth (564), rudska3443 (559), Piepie (559), jslykhouse (559), twistedtime (558), cattrack (553), admiralfury (552), eboyle23 (551), amy (546), ketta (544), auntdeen (544), timoflowers (541), prohri (539), harkiesvf (539), honbioKGo (538), taboen (537), seu1140 (535), fofomazuzu (531), larobusto (530), woaks223 (526), Christian_66 (525), jiberjabber666 (523), klaus13 (515), Dodam (509), PWPeebs (502), Mbrightjones (501), nashira (500), hank78 (500), mdtheater21 (487), randomlogin (477), noctivagus (477), rybaciagula (474), miriamel (470), nj4876 (469), cdaywalt66 (465), brandonberchtold (464), Leopar (462), lisainsandy (461), kukumuzu (459), martyy_plp (458), me (457), generalnotes (456), beatrixbloxam (452), dkstkdgus328 (449), npatnode (447), rightnet (442), knaray1 (438), jjw9706 (438), Unclewilley (433), mowens (432), pfenn (427), morphamagus (427), dont_blink (424), dlwlstjr333 (415), t3hnerevarine (409), kimjones1979 (409), qwenml (408), pduncan (405), gedrod (404), mottiger (403), mnbflute (402), alexmadsen1 (401), bradtaylor (398), Cerialis (397), rkdanswns (391), Sunnyway (390), 0303sb (389), rmissel (387), summer7656 (385), Ceri (384), qorqoreh (383), ksup (383), scoobi (377), kndahmen (377), nosers (376), Ditsch (375), g1smo (374), metamonkey (373), sisterscience (372), 01024984595 (369), pmh5954 (368), LynneC (368), hampton11235 (367), Hazelinka (359), staso (358), Jem (358), mh5052 (356), maxswiegand (356), momiga (354), dummypostfach (354), melodiebenford (353), spoonbow (352), jmonderer20162 (350), jjho9604 (349), redsoxwy (348), jwt1029 (348), jeoyoho (345), yernagates (344), annek (341), Arthemis (333), tennifry77 (331), dianasorg (329), Waffle01 (327), ccrowe (327), bwstudent (325), bunna (323), amyec (323), lilbundlojoy (322), hkc29 (322), xodhks9205 (321), sirpago (312), jmwc (312), amblingpilgrim (311), corvidae (310), jordichanovas (309), upcyclist (306), swoffler (305), youn6501 (304), alessandracva (304), lamebrain (302), MaciekB (294), vezz12 (293), tjwns105 (293), davis3792 (293), kimgtea12 (292), fidel (290), bmaco (287), tytoscope (286), mirandagavrin (286), honbiosra (285), duckgu92 (285), wjddls521 (284), vilo_grey (281), 9_th_grade_ninja (279), kenmierow (277), deniceb87 (277), Terramine (275), Vorlon (273), frenchkl (273), boomod (272), Jlovin757 (270), yoongsl (269), deadlyalgorithm (268), Ania19 (267), Coconuts44 (265), ItsNewToYou (263), mck6 (261), secondlawlife (255), tndusl3000 (254), dellswanson (253), AKhajah (253), rudfbf4752 (252), sbrooks_pilotmr (251), IsaiahC (250), edremy (250), Cliodhna (250), ssmtx6 (249), slated (249), Manago (247), echopapa (247), stihial (246), oroko (246), Jac (246), qk2922qh (244), jn291982 (243), radeknek (242), knzk (242), neuromancing (241), angelas (240), naspam (238), Manni_Mammut (238), Minokiller (237), elliesaysmeow (237), mollyb (235), scopedriver (234), chiflows (234), tobykenz1 (233), catherinePouic (232), mberna00 (231), cls6679 (230), chijunse (230), qkrw1 (229), richardk (228), Alya_N (226), wurk (225), Sekik (224), shamrockveg (223), elise (223), notenbed (222), clbriggs (222), dms1044 (221), mms502 (220), mkwak (220), lyndsey (220), monarchspoison (218), RosDan (217), raduban (217), megaschara (216), honbioGAl (216), minsak (215), bjan (215), vickywu (214), tamasi (214), guilifr (213), janicemilliman (212), 01085611037 (211), ramshorn (209), ross_bales (208), FMRTerrifc (208), LightPulse (207), duanestitt (203), elev (201), welo3 (200), nawaorl (200), millenniumb (199), ishikakushin (199), 8982679 (199), yev (196), neurosie (196), simonkaytamas (195), monet_open (195), lexerific (195), acidcats (195), ablasky (193), steenezel (192), lkauth (192), kastakan (192), pjoyce42 (191), mordodirosa (191), JHGFD (191), falconheart (191), mqrius (190), Freddo92 (190), mmichalka (189), kuzminski (187), pandabear (186), macdoes (186), jinsol6 (185), cestmarrant (185), sarabc (184), newneuron (184), ajemanuel (183), mjh1660 (182), lauli (182), sak3097 (179), jbarstad (179), Xandrex (177), lazymuse (175), sht301 (174), mariemacdonald (174), ldm2020 (174), Sagaraghosa (173), helge (172), MikeGB (171), kwangyong810 (171), ghkdls99 (171), hharder (168), LindseyAB (167), 5liwomir (167), whoheckhe (165), qudgns0014 (164), deliaknight (164), aimorai (164), EAG123 (163), asdf3011 (162), Magnoliahigh (161), chase (161), brianamywa (161), maby (160), dmacdougall (160), asnjhaley (160), rosapf (159), Kewne (159), SimoProvenzano (158), Hotline (157), yeppi2002 (156), shrajke (156), Sheepdog (156), aaiken (156), leehy250 (155), usagidark (152), tryeye (151), mru (151), serge (150), darcpixie (150), flouf (149), tunisiel (148), cyjing (148), acida_2 (148), elye (147), irinayf (145), twarning (144), magnuson (144), jujung27 (144), Crounus (144), wksung (143), ambermichael (143), Altaire13 (143), eroush (141), Caffeine (141), rutho (140), qkqjqkqj12 (140), honbioNFu (140), ffffrank (140), Aaroncoyote (139), lvova (138), keross (138), Kargoneth (138), wallrue (137), pennymonger (136), EricRoberts (135), ehsansabri (134), bouncy70 (133), backupelk (133), towever (132), LCU (132), Rosiebud (131), michelle127 (131), giselachristine (130), el_burnso (130), restcoser (129), jorwat (129), Robadd (127), caphillipson (127), Exotje (126), Neoronenblitz (125), DodamTest (125), copantok (125), jasperd (124), gdawg5130 (124), davidheiserca (124), seleniumsolace (123),

jero (123), ngiaopao (122), wjhsiao (121), djajsl1234 (121), wayner777 (120), bileduct (120), oliver007 (119), arachnae (119), taceywhite (118), kuzvo240 (118), jenh (118), Fhydra (118), ecairns (118), BoredRaichu (118), Danis_Bang (117), teapackage (116), hanami (116), McW (115), mapio (115), dksehgus06 (115), rkweir (114), jeonging2 (114), Cbay (114), Riley_Light (113), medievalman30 (113), ikarus (113), dsprinzen (113), meitaka (112), Flos (112), bkahn (112), laurippt (111), honbioSWo (111), arzi1021 (111), Trumpeter95 (109), webnajma (108), minisiebs (107), MindBlown (107), shackley (106), cyberdork33 (106), alexweasley (106), urs (105), JeyTee (105), stlbluesfan46 (104), sophiagerje (104), Waltika (103), Scot.a.murray (103), kernfel (103), olive (102), sjhy2050 (101), roro78 (101), mcsnee (101), tongo (100), HerDug (100), Geluemse (100), lyndseyannette (98), kjen (98), r3strictedArea (97), couc (97), shy960306 (96), cmr17 (96), chenzw (96), AlWAl (96), miriaml (95), rae.heitkamp (94), kohlman (94), jyjs1031 (94), dibbeke (94), alanisfer (94), sandrad (93), harmonicquirk (93), Cucumbersquirrel (93), blacat123 (92), dreadcat (91), caern (91), sambarko (90), ljk3053 (90), kobuki82 (90), zzahal (89), snail (89), msch.dk (89), Christophk116 (89), vaeltaya (88), Locutus (88), coconutO (88), Yark (86), skeptikitty (86), gadjo95 (86), auraseer (86), rbtrry (85), Martinqua (85), Bragsen (85), serilleous (84), robertb (84), orenico (84), HollyinAshland (84), es3ban (84), argento92 (84), shadow_8472 (82), Elcarim (82), dkutas (82), mrsmirrel (81), edderiofer (81), tupperharley (80), rafikh (80), moonsiri (80), danube (80), tracer911 (78), sylverone (78), czesiu (78), tjd0413 (77), rprentki (77), karenza (77), jw381097 (77), wlsgur7974 (76), s0nia (76), rigelan (76), ais (76), VADER (75), spes004 (75), irene7micro (75), theman2000 (74), Tatyana_Violet (74), slycooper (74), justagirl (74), jsb54 (74), 22nd_Floor (74), xrnibor (73), revel (73), misterjstewart (73), lgrochow (73), poen (72), paps (72), Mastica (72), ghi06064 (72), TomMarcotte (71), Randomfull9 (71), omen (71), kolalelf70 (71), kinryuu (71), GrandmereLouise (71), david.pavlicek (71), cjdeakin (71), Woong (70), sethiroth66 (70), mjeannez (70), arc330 (70), theneurax (69), kwondsu (69), kwon3693 (69), kite86 (69), happyyj1997 (69), blowfish (69), invest09 (68), cody.rice (68), chickleodeon (68), phronimouse (66), angie12321 (66), peters345 (65), pbailey (65), Noeska (65), MikeyG (65), Trefling (64), SusanCotton (64), peter1986 (64), nsi04118 (64), lookanelephant (64), Ivet (64), DigiAspie (64), conrads (64), sthomson06 (63), noosey (63), mcrisch (63), Ma.K (63), lizaperks (63), Emma_B (63), CobaltZeroni (63), rekrab (62), McMasterslayer (62), kiwichick (62), nayoon0117 (61), Konafets (61), educmale (61), colinra (61), ZonaCat (60), ImAPotato (60), evanilssen (60), Ania89 (60), Szelma (59), OrionWolf (59), jaro87 (59), deltapapa (59), Swiper (58), srhjk (58), rhn (58), etgst (58), bebe1817 (58), heskey30 (57), emsabo (57), eatmorepickles (57), care (57), x9q9c (56), tomoyo_ichijouji (56), quetsch86 (56), billzhu (56), _ (56), zyfi (55), tomiks (55), pascallev (55), lindanicolette (55), edoody28 (55), cvanveen (55), ValdingerBella (54), snik (54), Ronnie_Soak (54), Dr.Ron (54), Calycaa (54), rsp (53), msully98 (53), chy1000 (53), anneli1221 (53), waterbug123 (52), TheOnlyOne (52), shelby2190 (52), matollik (52), hleisma (52), gabba (52), Teepfluecker (51), LEHoeger (51), zhfldk452 (50), vogon (50), looker (50), DmitryMalkov (50), dlsdl1210 (50), ccolbert (49), smalljude (48), kes2521 (48), Brasa (48), slhszlls (47), richarrr (47), jojoful (47), dewondolynia (47), Tirla (46), stingypanda (46), sofpav (46), nurtwalder (46), junsung1250 (46), haptic (46), cassandra87 (46), Byuda (46), Albert_von_Herford (45), Simbertto (44), Emirald (44), dracttosh (44), sapphiresun (43), ree (43), Nyabi (43), lemunet (43), eluchinat (43), candychewning (43), adh02us (43), sikan92 (42), Nemtsevp (42), it_ohs1018 (42), Davhornfir (42), apmontagne (42), 01044645208 (42), TomasMihalek (41), nisanick (41), IgorOhrimenko (41), cosmicdolphins (41), cksgh645 (41), cityghost (41), Anastasia1 (41), tajia789 (40), leraoesa (40), kazarenko (40), gamez7 (40), Zitoire (39), Shmease (39), cathy43dti (39), carleye (39), yourion (38), skye2520 (38), saeb101 (38), PersonalGamer (38), maciek0899 (38), arrosa (38), Wincey (37), tets (37), sorgd (37), kingboyd13 (37), fgyo (37), cedric (37), Tomghc (36), tmdgus0577 (36), sfree4all (36), maastolman (36), learnedhappiness (36), Engine (36), ender2336 (36), satoriend (35), mirandakarson (35), joac (35), GregO (35), gparker321 (35), bugmenot (35), yanshan (34), ube2o (34), Richard_Henley (34), renate (34), metalstorm (34), Maye (34), Lochnivar (34), FestinaLente (34), Ererexiue (34), erebus (34), awakaba (34), 4nushaak (34), townshenge (33), Togo (33), mdailey (33), logicality (33), jogarza (33), HillObserver (33), Flocktime (33), bethleegy (33), thinkertank (32), denisehigg (32), charalampos86 (32), awakn (32), skruffylooter (31), SATLas (31), MagneticHammer (31), IRSmartKittyz (31), Gozgo (31), coelacanths (31), audreyreale (31), annie3472 (31), tsandy (30), thehoboclown (30), rksdlqkqh (30), narvaezm (30), ladykaro (30), evin (30), BladesRUS (30), yjyj1023 (29), thegreatgarbanzo (29), shakey_man (29), RM (29), maggielnb (29), laurivan (29), epistygne (29), AnaelG (29), Zarkark (28), titousensei (28), TheOne (28), sparkst3r (28), Plaidomatc (28), miguel.jodra (28), kibouhi (28), kevkingofthesea (28), hklingon (28), Headofibis (28), TPM_23 (27), tomy515 (27), Richtefee (27), kt1004972 (27), Keiken (27), katja (27), hippydogmx (27), gadwicke (27), Boop (27), tishow (26), louiscyphre (26), kubakoniec (26), brinkhm (26), beammeup (26), aco (26), Spit (25), Simulant (25), Nibbler85 (25), jeffyap (25), jasonsmiegel (25), idril.piesdeplata (25), gentrymc (25), gdnskye (25), Falant (25), DannyScythe (25), alisacrisp (25), 4A85E (25), wwwmkstr (24), spagls (24), rillabee (24), LuaCat (24), JessicaS (24), Janner (24), jane.matsesha (24), goodjelly (24), gardenpea (24), equus1556 (24), dischordn8 (24), arlind (24), Ariodante (24), anyep (24), wvdamme (23), whaleback

(23), werecake (23), Sigmus (23), saxywolf (23), sagebrush (23), momadoc (23), minnu (23), Mateutek (23), kseif-naraghi14 (23), kcutshall0710 (23), heatherandjim (23), ggaffield (23), dwarshaw (23), charna (23), big_sasquatch (23), 12261264 (23), psh0944 (22), mrnibbler (22), kenardX (22), fairboxie (22), Caprise (22), brock (22), box213 (22), anmbia (22), 65sprd (22), Terrag (21), Samaelle (21), Ring3r (21), NeuroMS (21), Napis (21), lubaluft (21), jasonpapacostas (21), Hundas (21), hot6138 (21), ... (21), robz90 (20), RetinoidX (20), overk1ll (20), jongmin2 (20), jnpy (20), iam1105 (20), gemmi (20), gasper (20), eyjaring (20), carolmswiz (20), yby6338 (19), vlorbschnat (19), nashbery (19), Mewidy (19), melissa0774 (19), kockas (19), karijnk (19), Incubus_Mirage (19), gahg (19), vitsen (18), ravissante (18), muh1117 (18), lilyshield (18), jeantherapy (18), helmars (18), canca (18), bcnissley (18), yottaflop (17), shpark2153 (17), Oguona (17), llanger (17), grimjar (17), doomed (17), dioneuty (17), christiantaylor (17), with-aar (16), taryntelle (16), Nysha2 (16), kisu (16), hermes (16), hcmcminn (16), TinaS (15), pkly370 (15), peachbed625 (15), merlmoor (15), lunarhyane (15), lunaluna (15), Jpassionforknowledge (15), jonnableel (15), devn (15), dark-champ (15), cirbi (15), blueambit (15), azoarcus (15), Anja66 (15), xasapula (14), Wendybird09 (14), sungwoo1230 (14), sothe (14), ravenshadow (14), mm523 (14), marygriff (14), jurkov10 (14), hana03 (14), venerac (13), thebar-racuda (13), Roboterbastler (13), mufintime (13), msblue (13), m4573r (13), kittysu (13), freesong (13), elgato337 (13), dalcomlua (13), cbear59 (13), 4onen (13), zchan5 (12), saspist (12), Nseraf1 (12), MissMilou (12), jin01056 (12), Griso (12), gahh (12), eydel94 (12), celiad (12), byledojutra (12), boyan (12), bodrick (12), bobbygirl (12), vi-enna717 (11), verity (11), thajazzlady (11), superrogin (11), spunky11 (11), sp4rt4n117 (11), reddu (11), Neonnq (11), Lilliscowleen (11), LegionMammal978 (11), lari_lh (11), igor_chebanenko.72 (11), furball13 (11), efhw (11), eee7016 (11), CLDP21 (11), ciaodejan (11), zkdlxh1532 (10), zelnicki (10), xy479why (10), songbabe7 (10), smithweiss (10), nb02531 (10), kkezir (10), Kayarewhy (10), JacqR (10), guswn5828 (10), Galestro (10), corvus1 (10), CathyK (10), Bibou (10), tjiannone (9), sbgowin (9), romses (9), marka (9), lfouquette (9), jacobscaff (9), EdHolland (9), daggsta (9), Braineses (9), bluemchen (9), beagol (9), awdzsx (9), 136andon (9), 0din (9), youn9023 (8), Stichflamme (8), sorek.m (8), solange (8), PRG3D (8), mpop (8), Moltodor (8), MadBlack (8), icewindhunter (8), GrimReaper (8), elephantrx178 (8), Deety42 (8), danis (8), curiousimbroglio (8), aubrey (8), vnetox (7), svedge (7), supertiger (7), sunjess89 (7), slslgld0 (7), sayaka (7), prowlerath (7), nernio (7), naro (7), mlsowers (7), mind_less (7), Luft1990 (7), ksmason71 (7), kkh20nice (7), kd7937 (7), JWC (7), Jeff343 (7), Janovic (7), hoursanov (7), grendelkhan (7), gitsum (7), dejan_dejan (7), ddil (7), codingTrickster (7), brisance (7), 4df (7), zespy (6), yawnG (6), wolven_moonstone (6), sa_sky (6), rileyfranks (6), riddesh (6), pursuit88 (6), okto (6), nawre (6), masterweaver (6), kipmacsaigoren (6), jig-sawmonster (6), jeremyvicencio (6), ian_danskin (6), hwaaim (6), hexidecimal (6), crewpet (6), caller (6), Arlelka (6), alexgotsis (6), alex791 (6), Voxelus (5), superdopamine (5), seth (5), osamc (5), mlayten (5), mjvonstein (5), Miles349 (5), meechl (5), Lutinka (5), lucas73 (5), lubos.odraska (5), lihwei (5), legrandchef (5), korenm (5), kisuklee (5), kate38 (5), jwhyb (5), jackrabbit (5), eyeguy2001 (5), erhome00 (5), dragon7496 (5), dltldud0 (5), darkshadow44 (5), CrashxD (5), colleencat (5), BlackPhoenix (5), bandg8t (5), asdavis1 (5), alexae6 (5), ajokewinks (5), TheBigZocker (4), Tallen01 (4), slgonzalez (4), schjora (4), owenga (4), ninanemila (4), MoreInput (4), Mccara (4), marcot617 (4), l711006 (4), knw257 (4), kkim9932 (4), KJG (4), kfailor (4), kat646322 (4), jungsicy (4), jumpkeh (4), jmaster (4), Jhe (4), Jamoni (4), Gully (4), girliegirl1991 (4), Frestil (4), EnriquedellaValle (4), dyordan1 (4), drbyte (4), dlwjddns5 (4), blue_minded (4), ayoung27 (4), Atani (4), XebeX (3), whomp (3), topspot (3), toby1p (3), tmeekins (3), thingy (3), sridharsm (3), ruthal31 (3), ronno (3), rankinc (3), pierresolide (3), philrod94 (3), Pat0alex (3), ozabluda (3), nemerle (3), NekoYasaka (3), narmi29 (3), minime123233 (3), mihaib (3), mcfly (3), marius31 (3), Malakiash (3), Liuu (3), lh2iwire (3), lemmiwinks (3), KaveyKaveMan (3), jerryam (3), herbertg (3), frankhold (3), engadin (3), dmg04158 (3), crusaderv83 (3), casevillan (3), calvis (3), brittanygs (3), britishclimate (3), blackbox (3), a_pache (3), ancarius (3), aljen (3), AlexFranco (3), alexetciboulette (3), a11fred (3), yuval1 (2), xtrapwr (2), xpfjs1028 (2), wolfspirit (2), wesselinator (2), wappentake (2), tutorialtest (2), tsien (2), TheGrinch (2), stalwartspy (2), spriteling (2), seldara (2), rlnhernandez (2), RiftingFlotsam (2), retribute (2), readingite (2), rahabg (2), Psychlone (2), ossomas-ticato (2), nkem_test (2), ninavelka (2), nerfherder (2), nephyst (2), na1690 (2), mon7468 (2), mogueta (2), MickyC13 (2), mevlana (2), lulugoesboom (2), limefox (2), larwloszka (2), keyeri (2), jtneu77 (2), jinxie2300 (2), ibisenc (2), horakely (2), golfluvr (2), Goldsternchen (2), gjtnwls0817 (2), gigiz (2), flamingomarty (2), FieryCuber (2), euan466 (2), EngineerGamer (2), davidmadsen (2), danserig (2), coryg308 (2), constipatedbunny (2), clarkphd (2), brianmk (2), ashrai (2), arwenm (2), ariverr (2), zeljko (1), whitetiger90 (1), whathecode (1), weatherdude (1), vedrance (1), urielxvi (1), Uendel (1), trinick (1), tickytocky (1), testtesttesttesttest (1), tatscub (1), songsmith (1), smos2022 (1), skl6284 (1), sith (1), ringring (1), Rae_Glover (1), qwaszxopklnm (1), peterahn (1), petels (1), parhaml (1), Olman (1), Olgierd_Swiecaj (1), Nseraf2 (1), Nocturn (1), mushufeeney (1), mugzyrae (1), mrjrgregory (1), MooCakes (1), mitja (1), mine5959 (1), mimiheart (1), migueldc (1), miffed (1), mhutson (1), meanae (1), mayzor (1), matkne14 (1), ltdrum (1), lpierre (1), littleblitz (1), lewisishere (1), lemmert (1), LD2 (1), Landselur (1), KR_VaJil (1), kintrbr (1), Kilrathi

(1), kevinwitak (1), KayFires (1), jzezel (1), jsm1310 (1), jrj626 (1), jfpickard (1), isagolden (1), huhe (1), Hokepoke (1), hillary625 (1), harm1995 (1), hanes2002 (1), hanaa (1), gusgraham (1), galarun2 (1), fowler.a (1), foomp (1), filipeyro (1), ferkal (1), enavarrocu (1), devilwaydown (1), datacute (1), darajoe (1), dalgaier (1), cszlotarybka (1), crazyjohn (1), cmbarnes (1), cmahung (1), christie (1), cherip (1), CharlieLeeLee (1), bruceware (1), brinlong (1), beckeemo (1), BaronvonZucker (1), ashtest (1), anginehb3 (1), alicia_11_11 (1), alexbenjm (1), aelscha (1), adelka (1), acrabb3 (1)
