# Supplementary figures and images for: Analogous Convergence of Sustained and Transient Inputs in Parallel On and Off Pathways for Retinal Motion Computation
Source: Cell Rep. Author manuscript; Available in PMC 2019 Mar 7. (PMC6404534; doi:10.1016/j.celrep.2016.02.001)

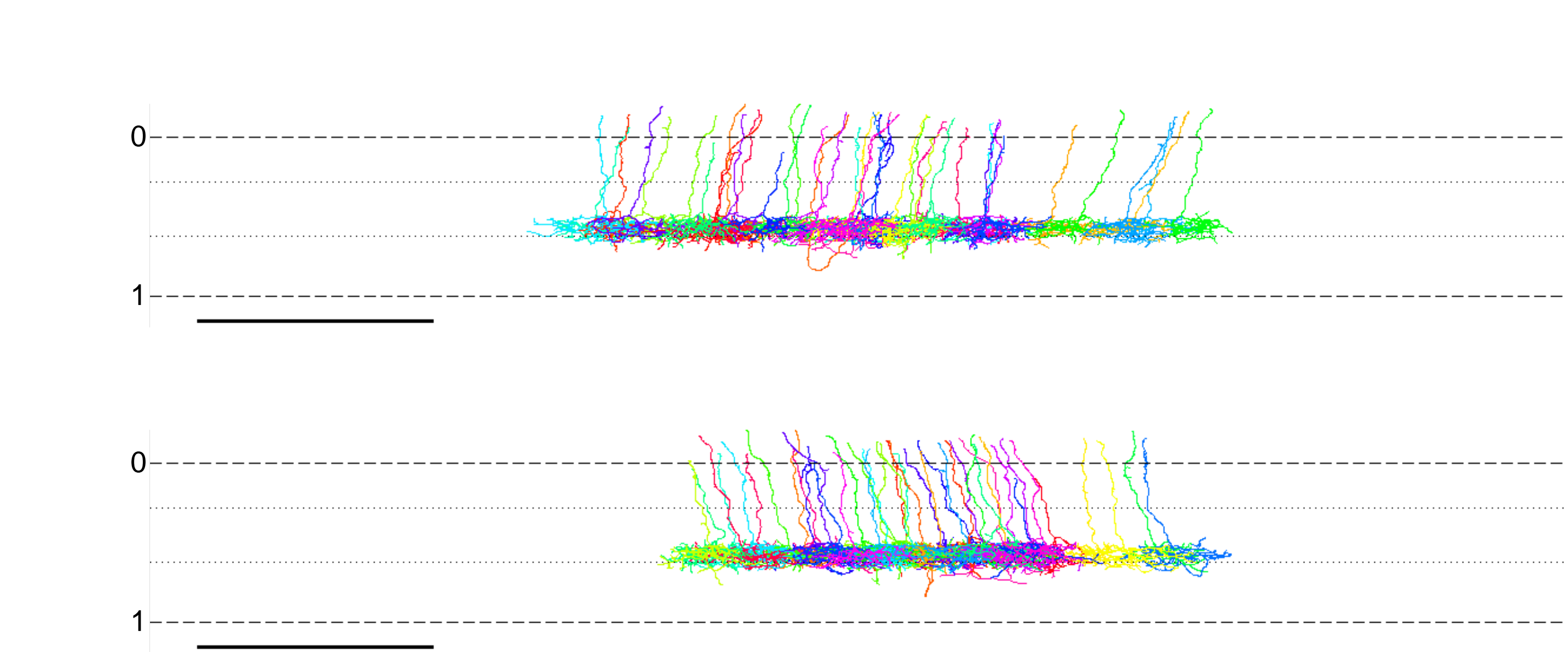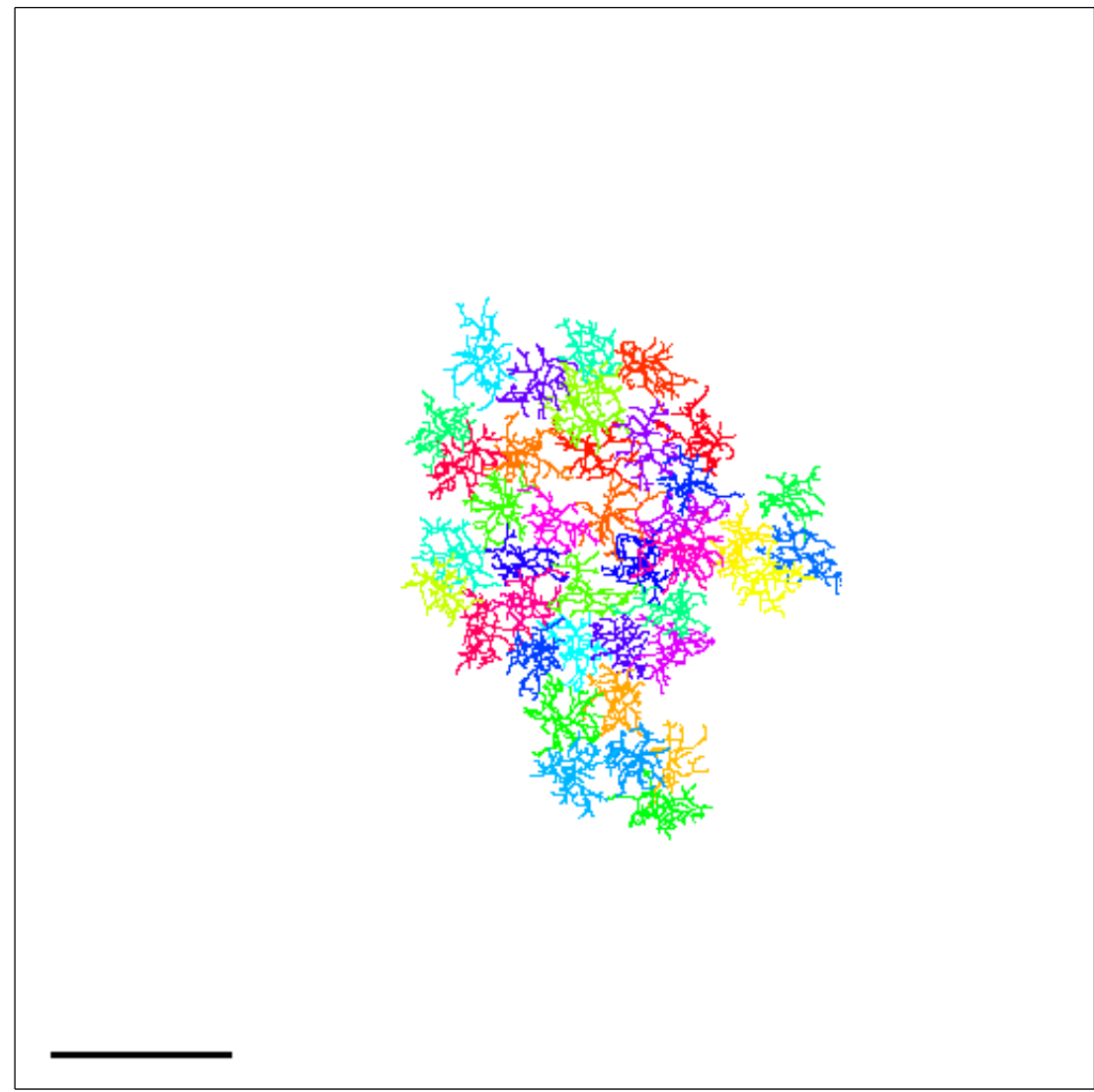

BC5i

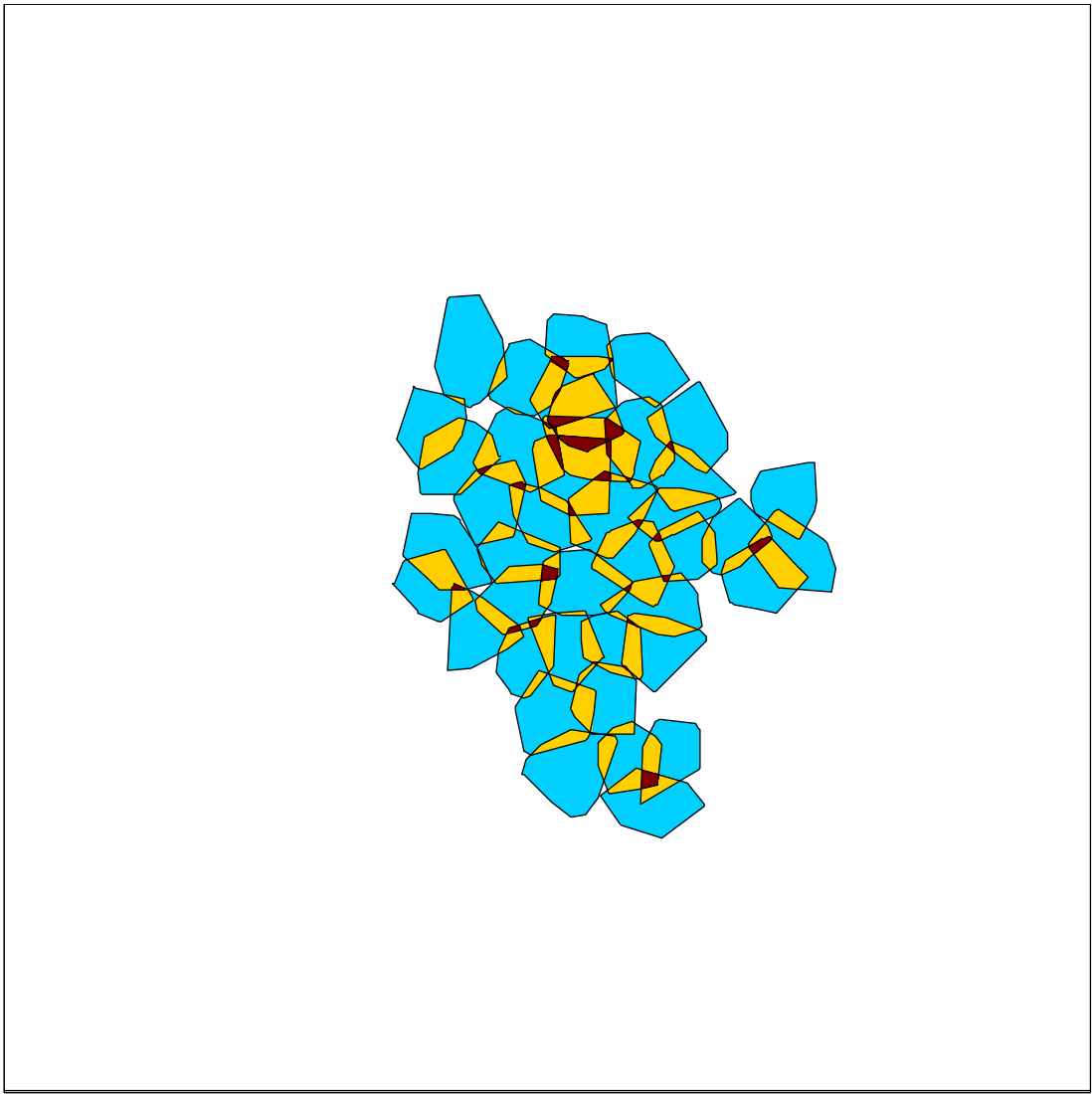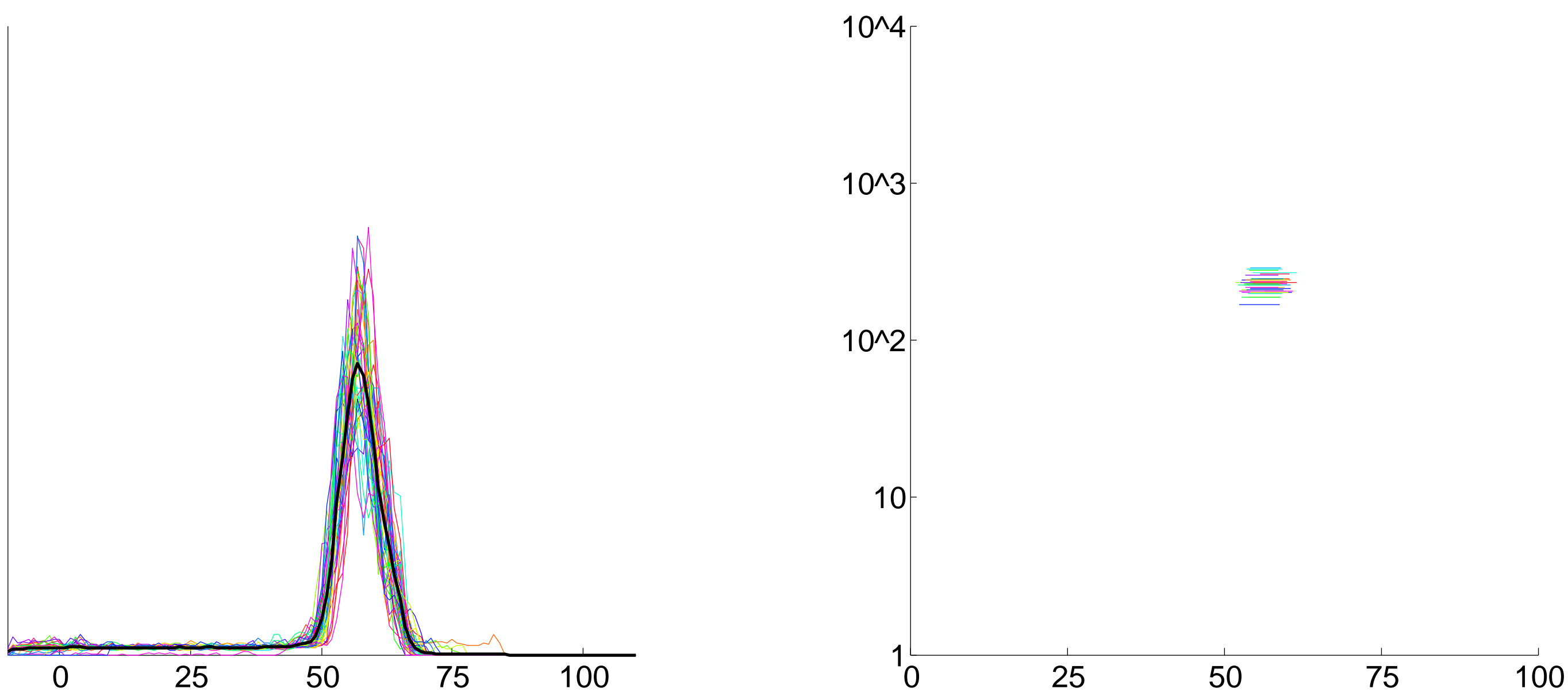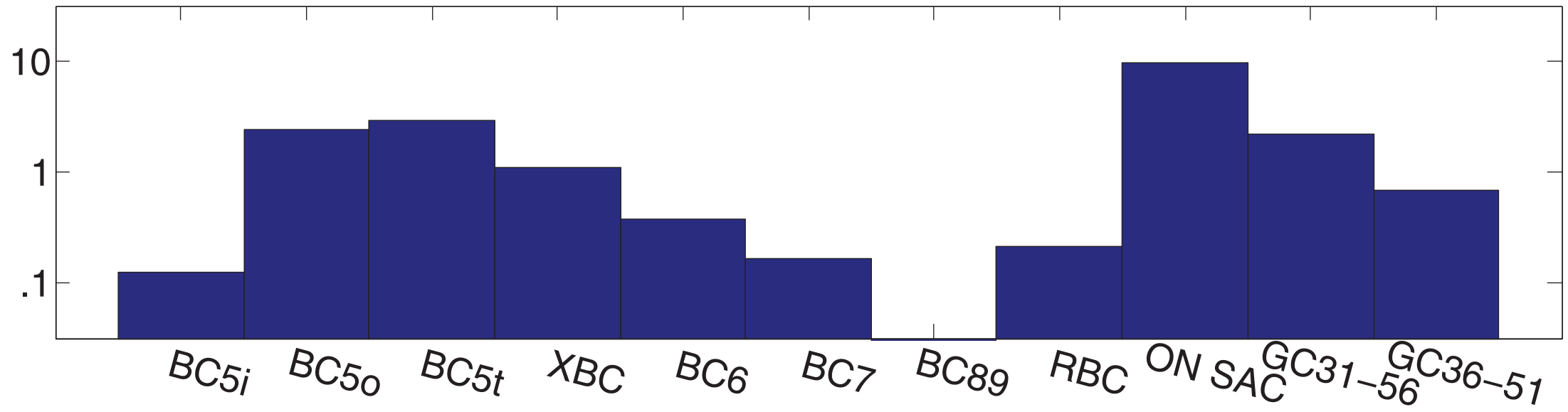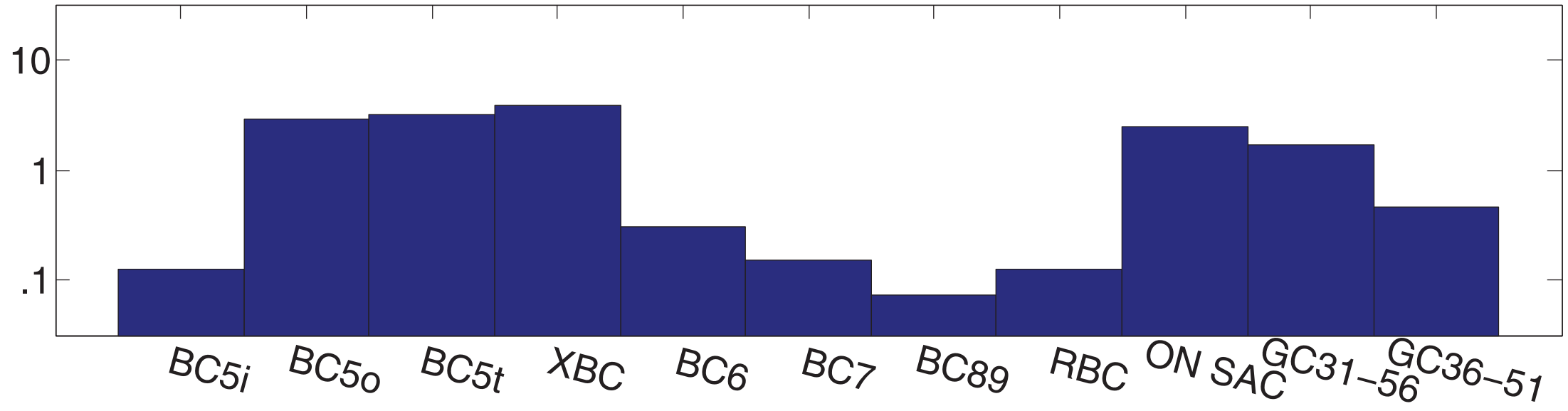

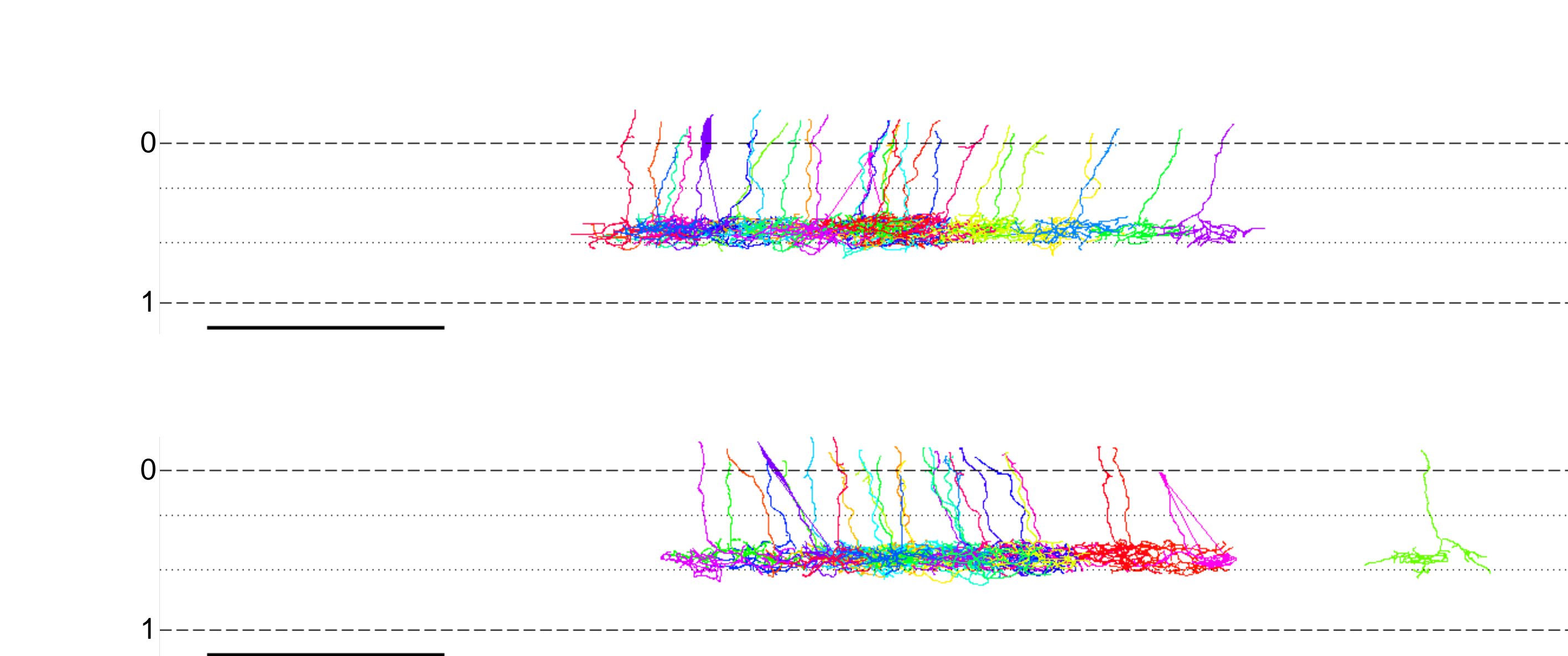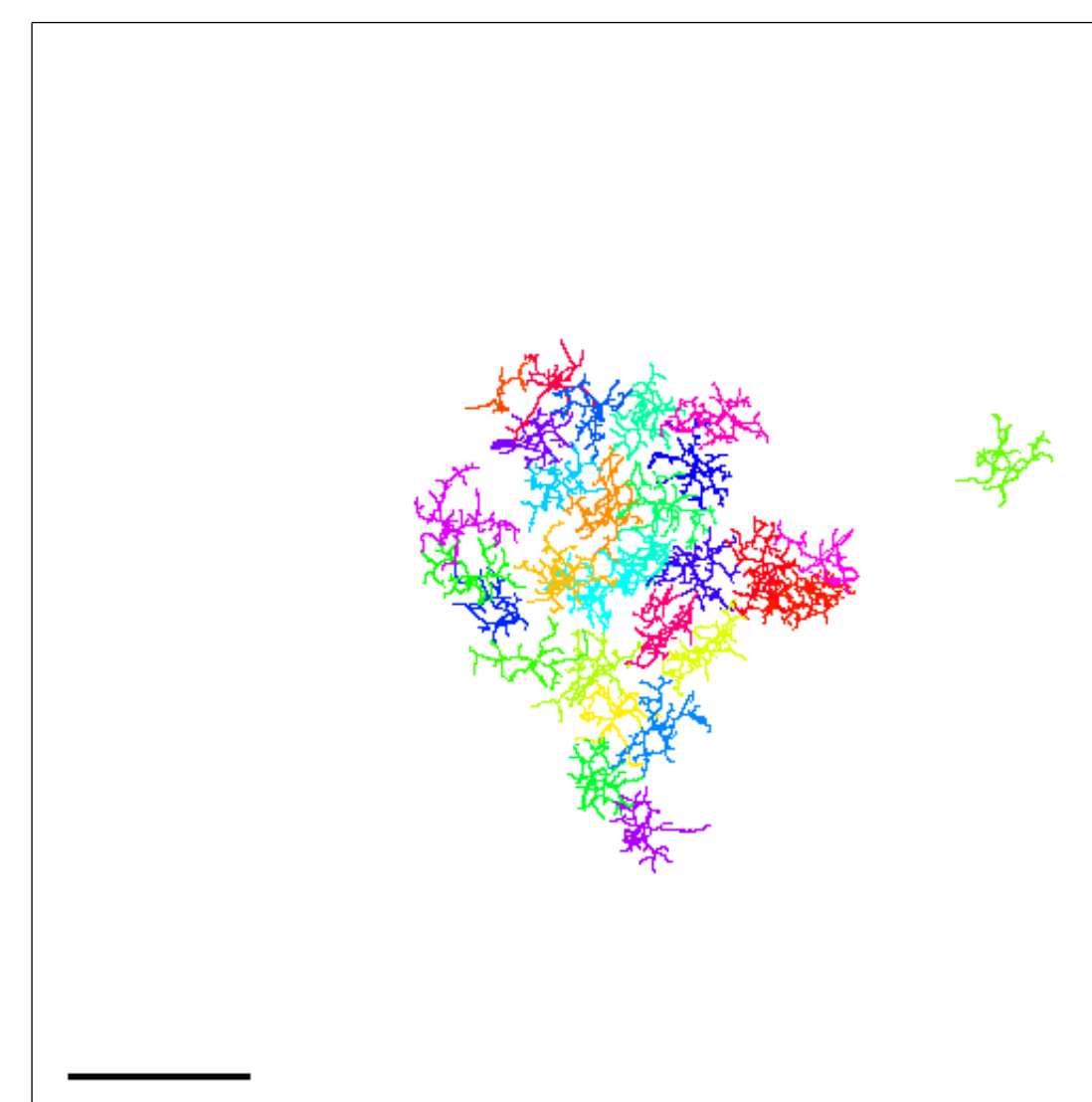

BC5o

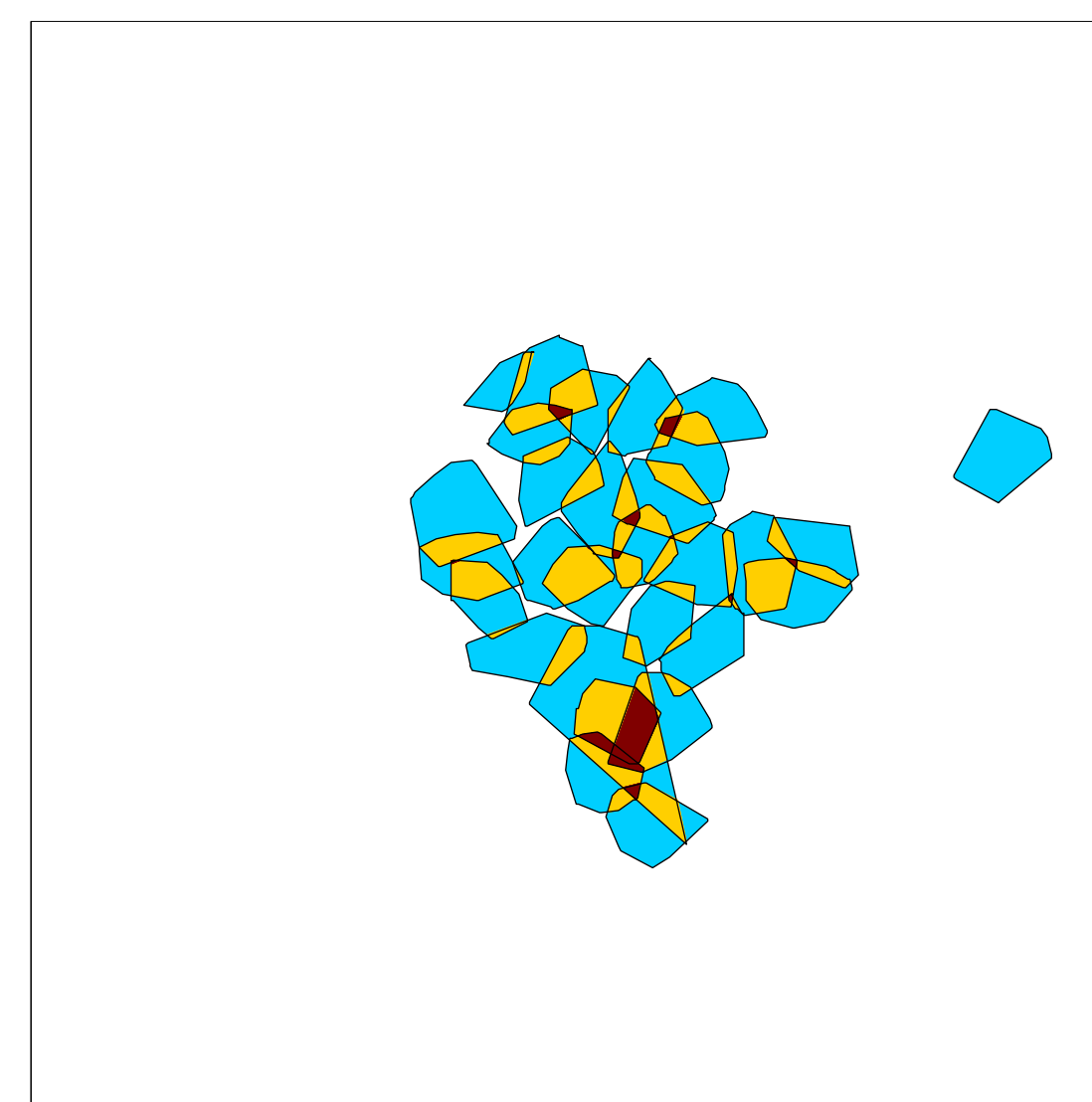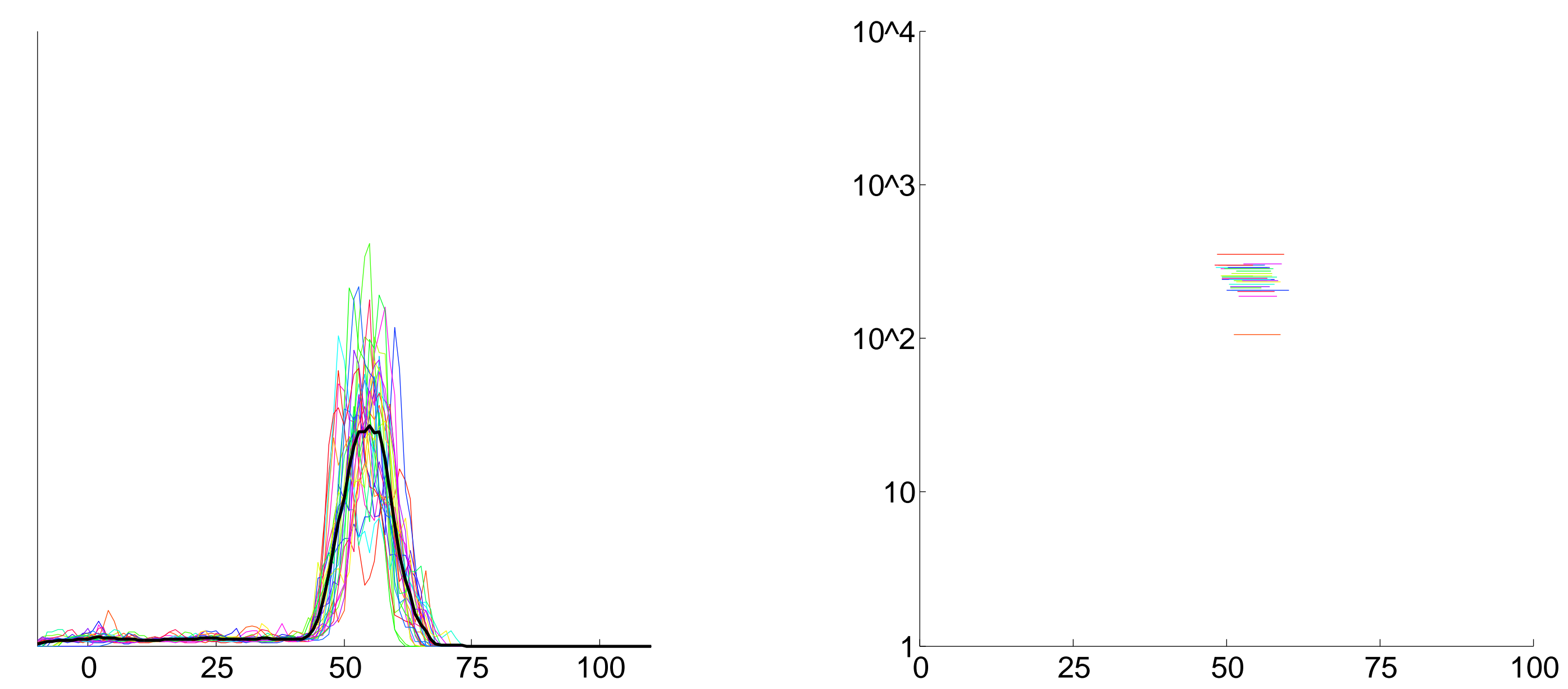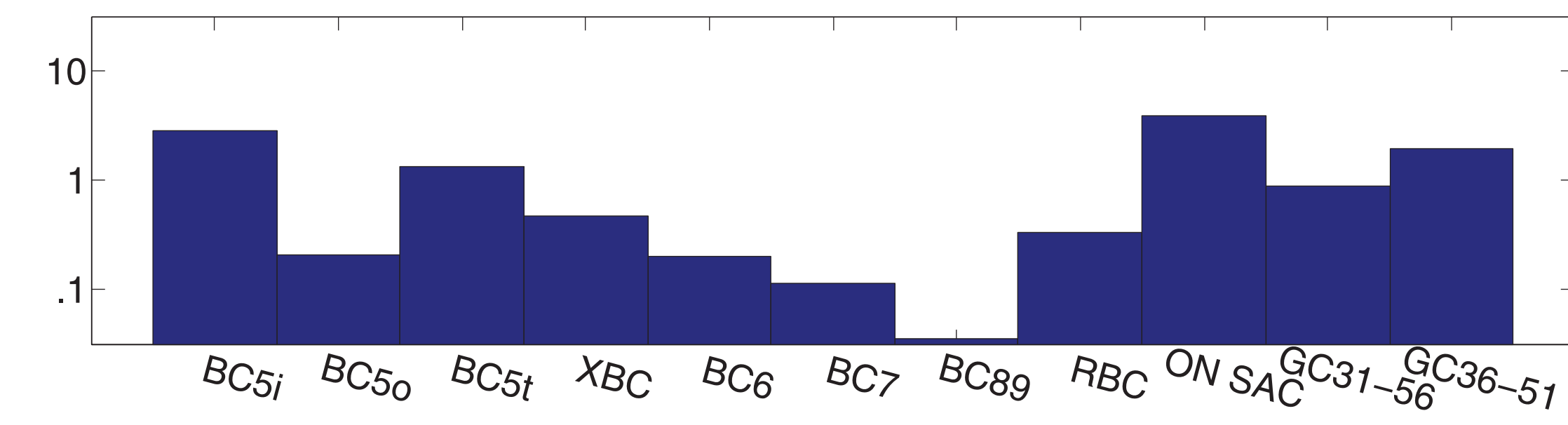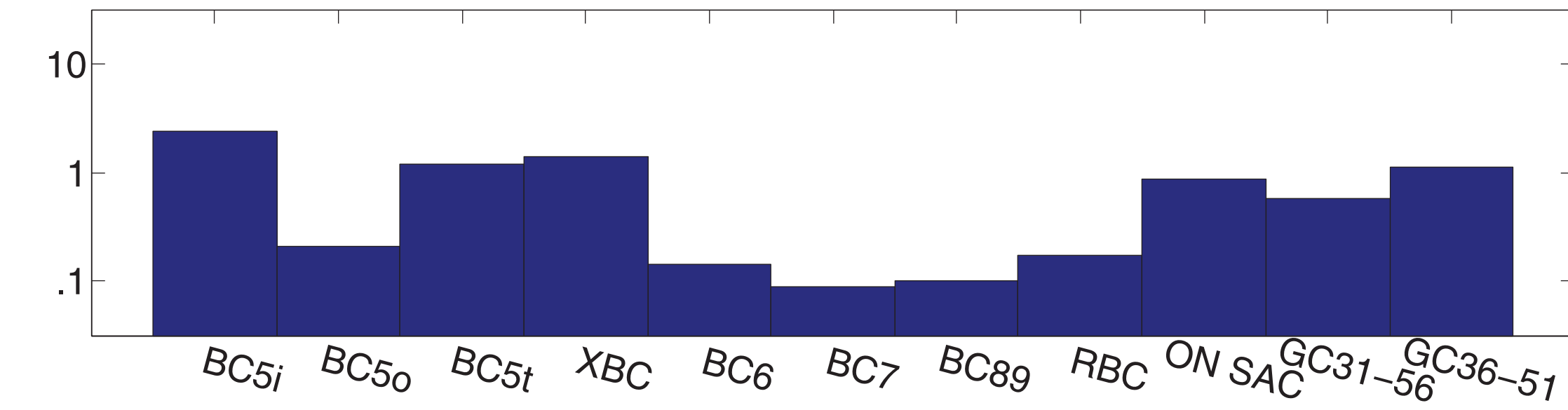

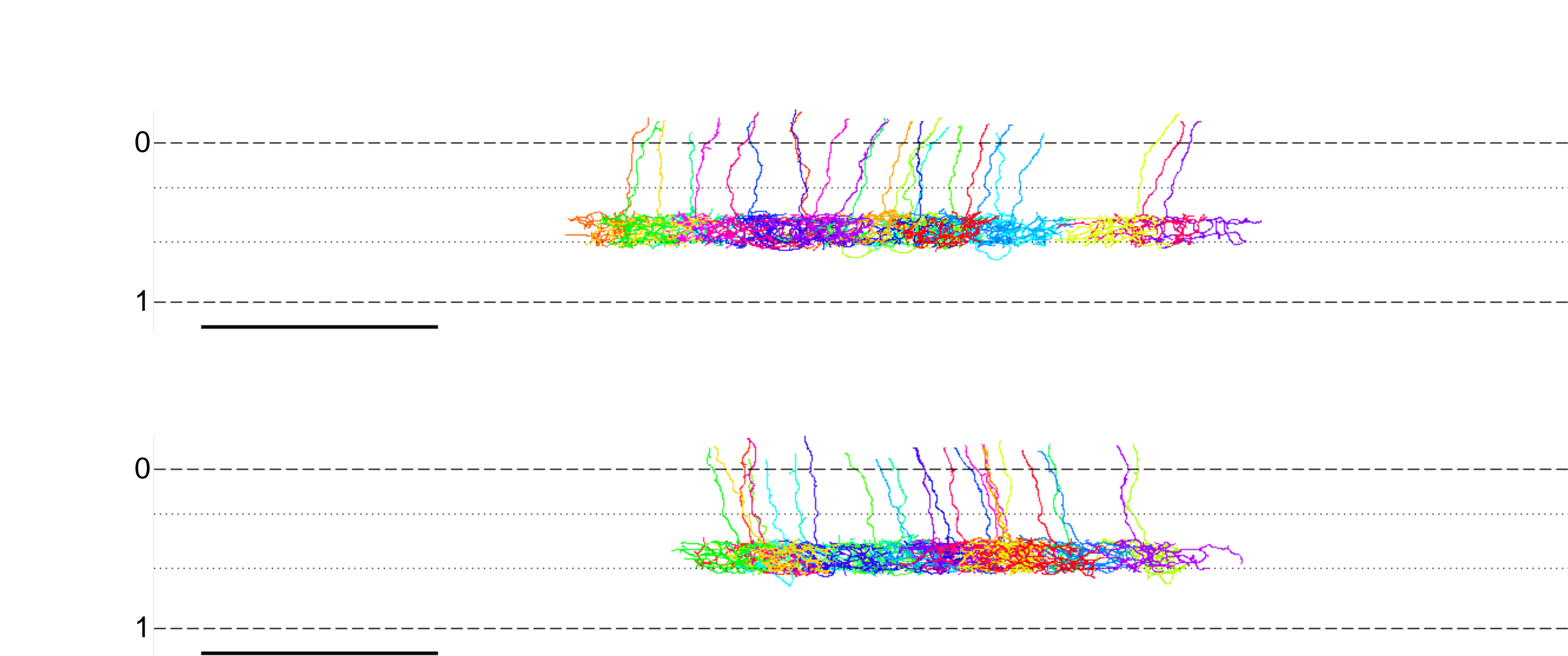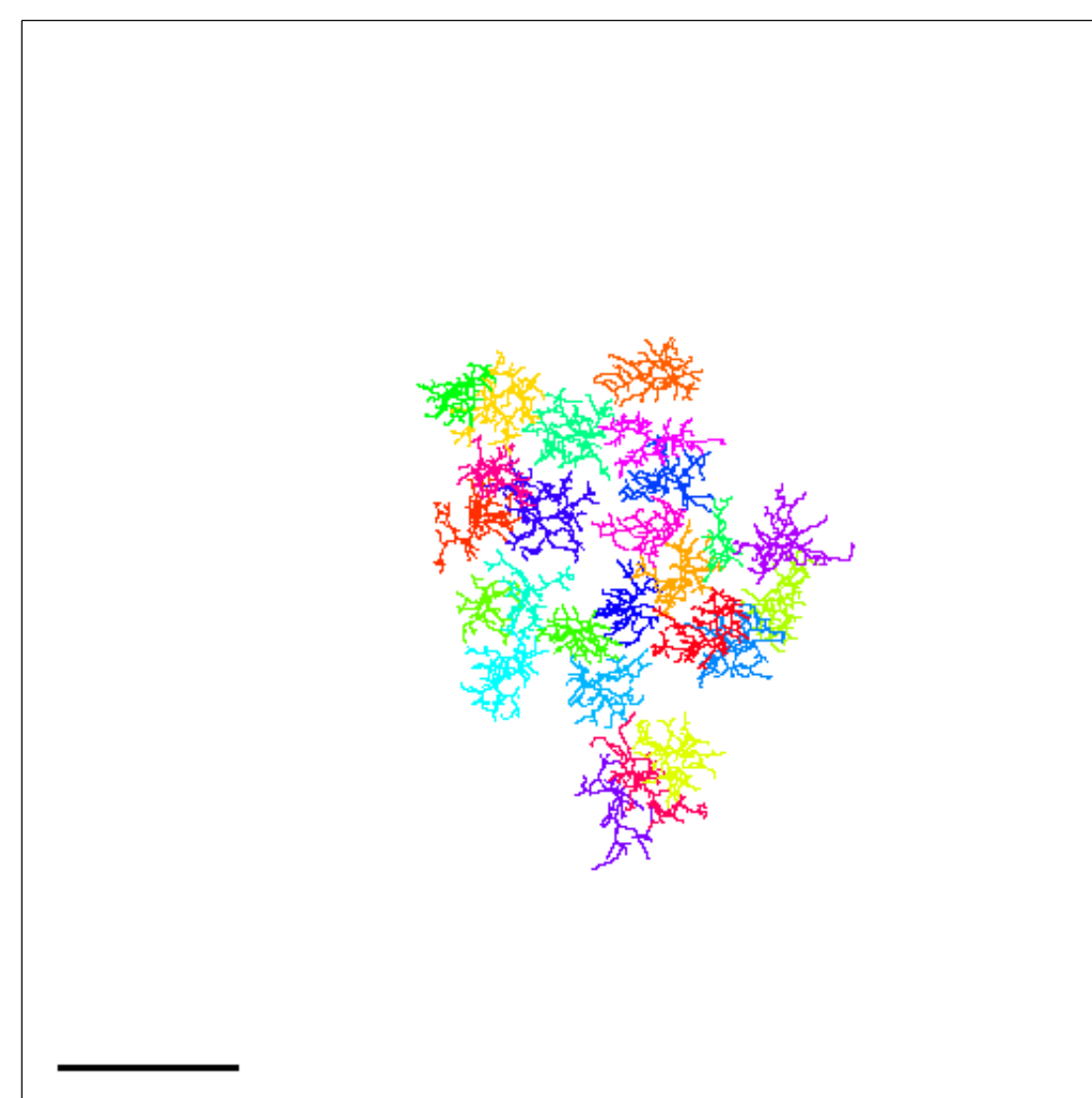

BC5t

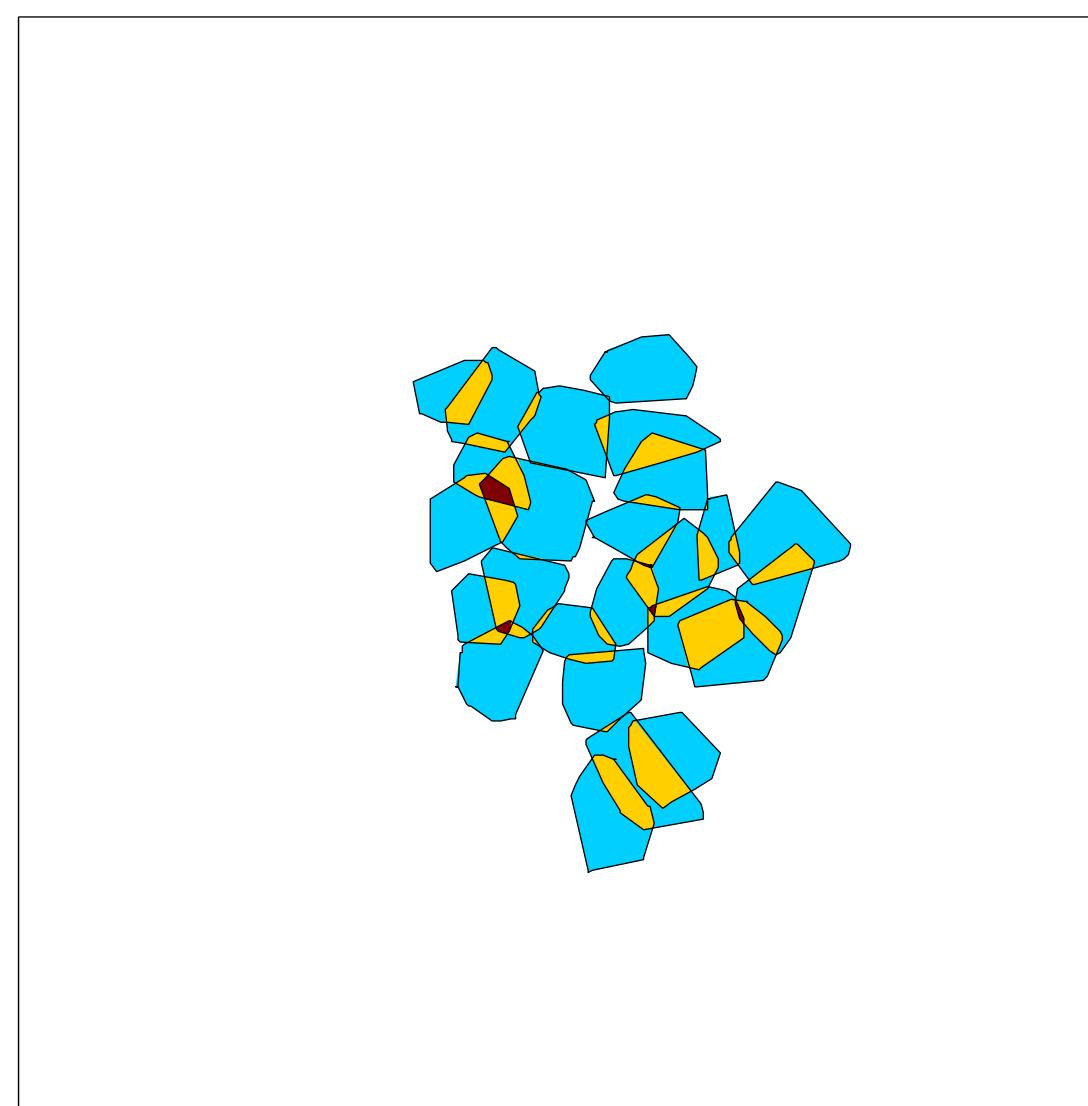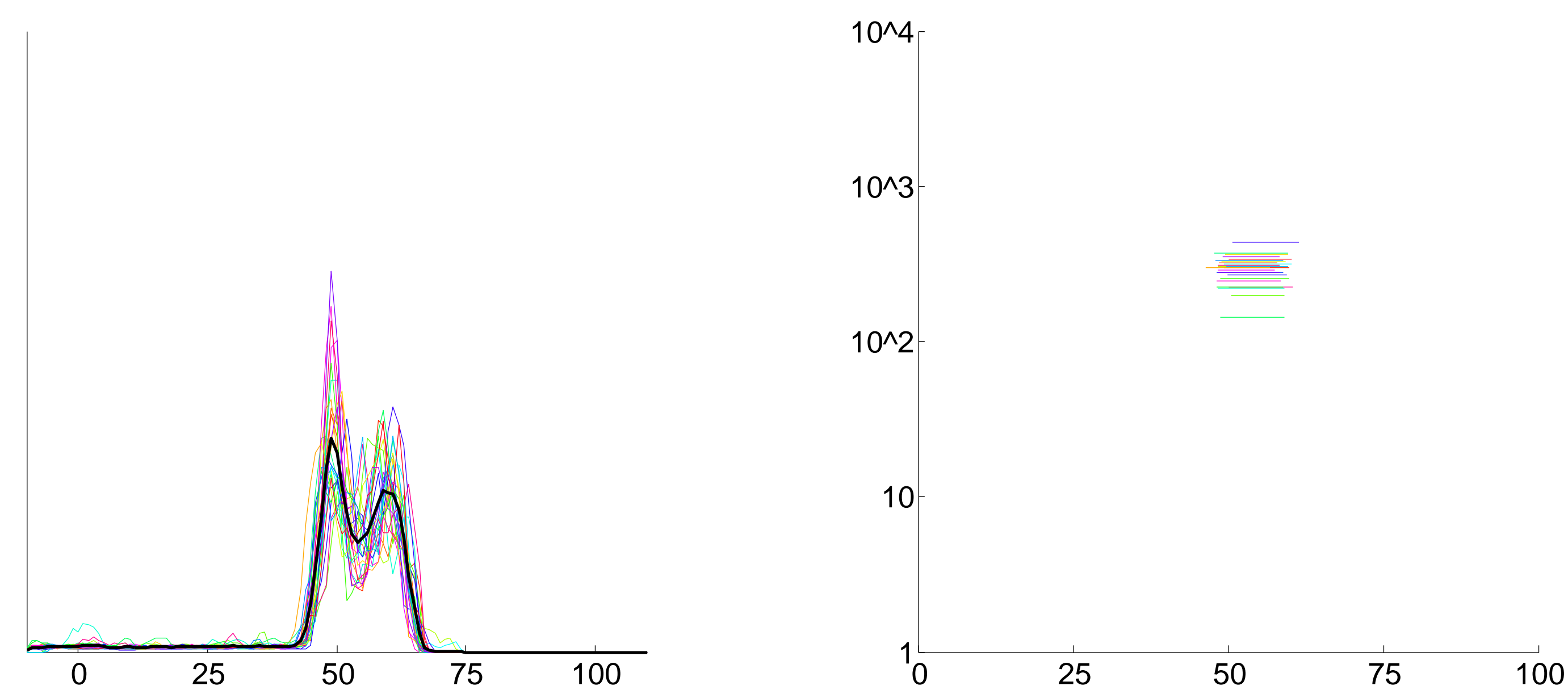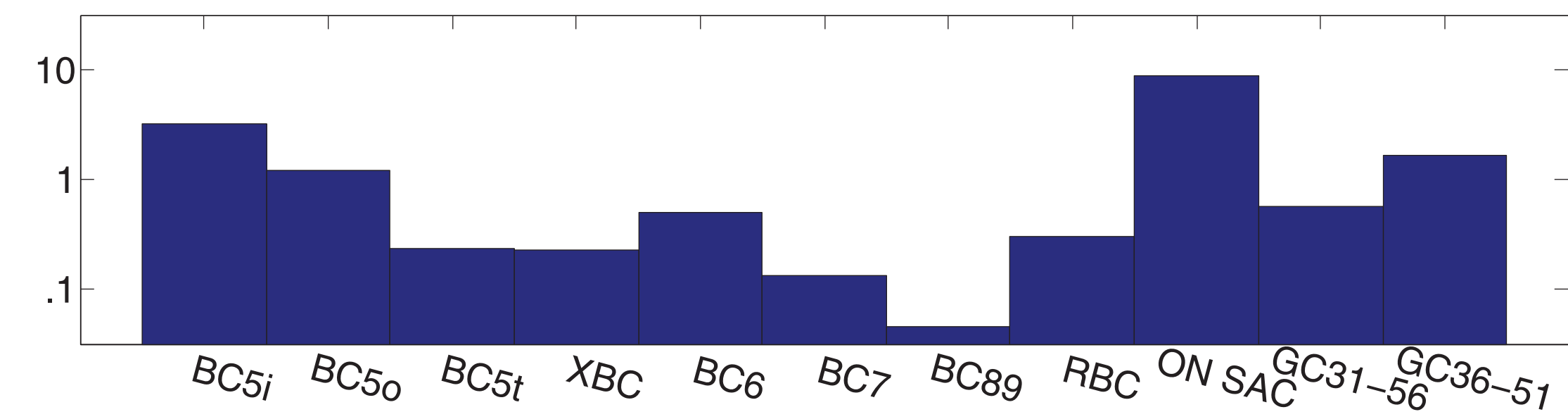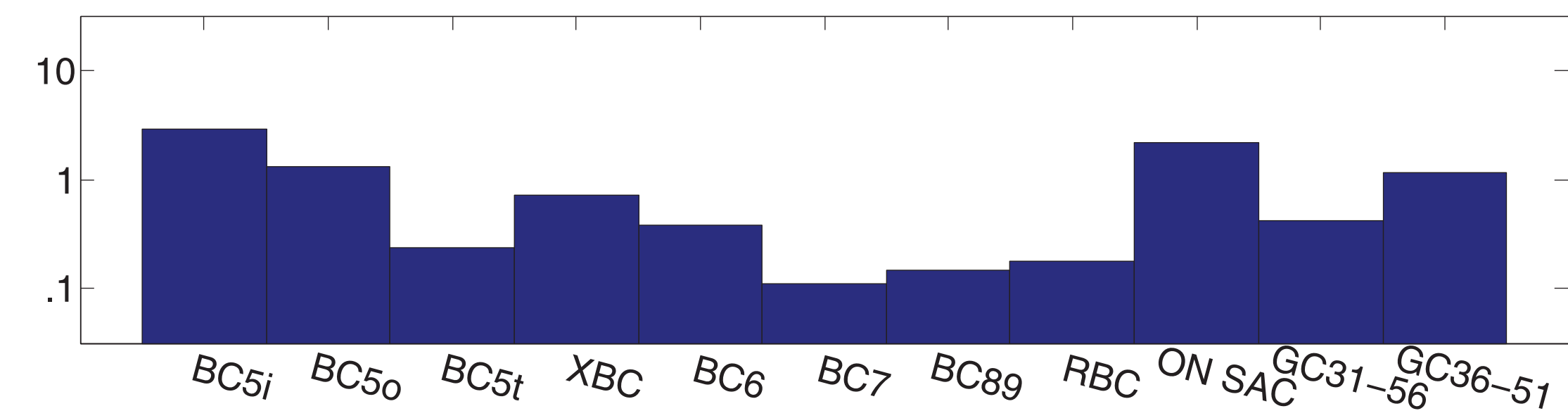

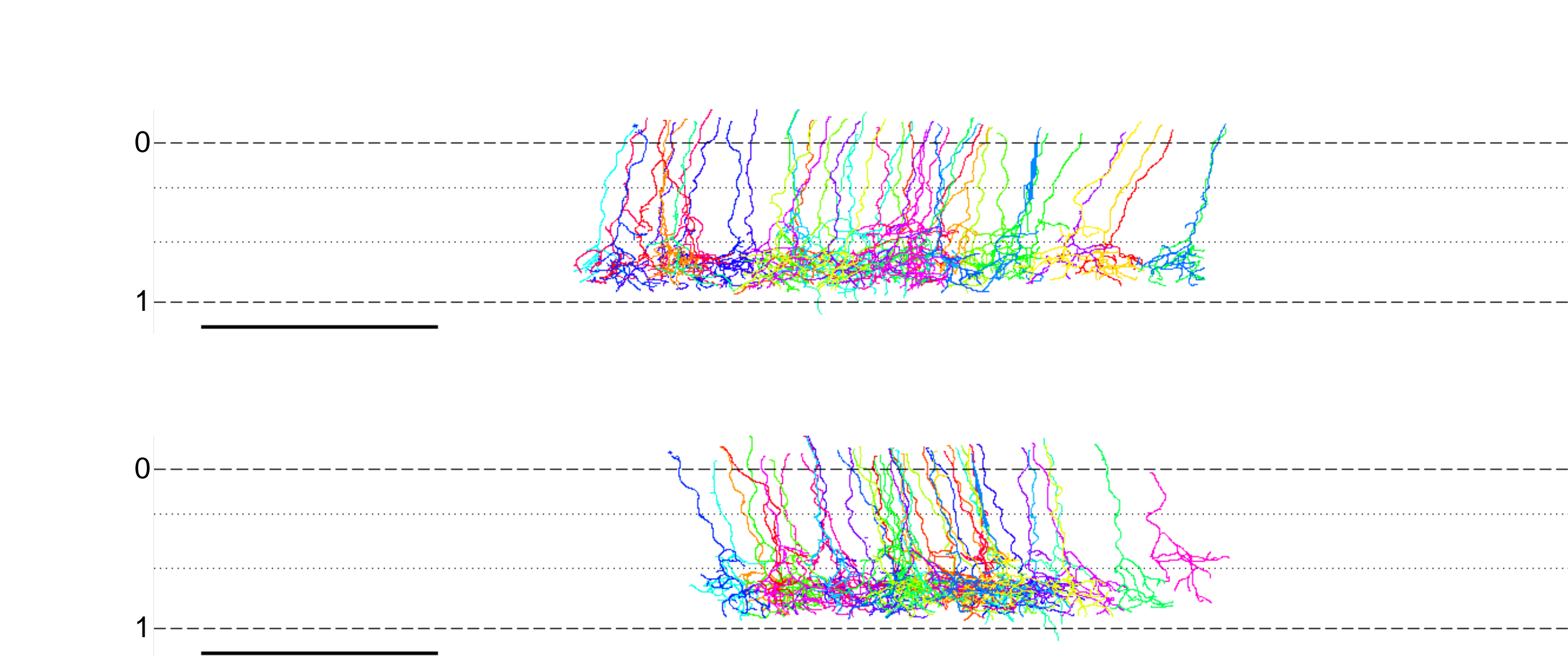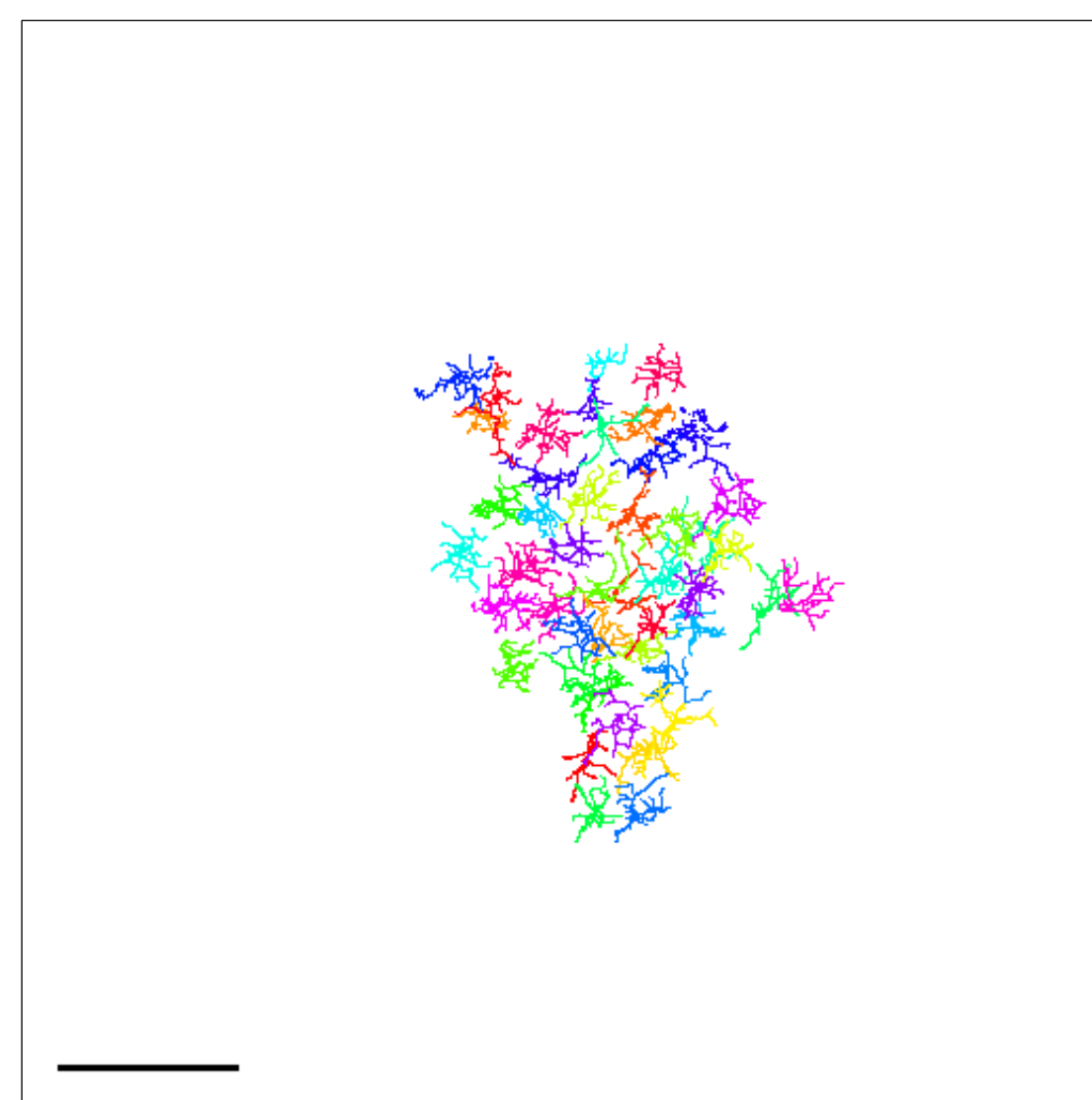

BC6

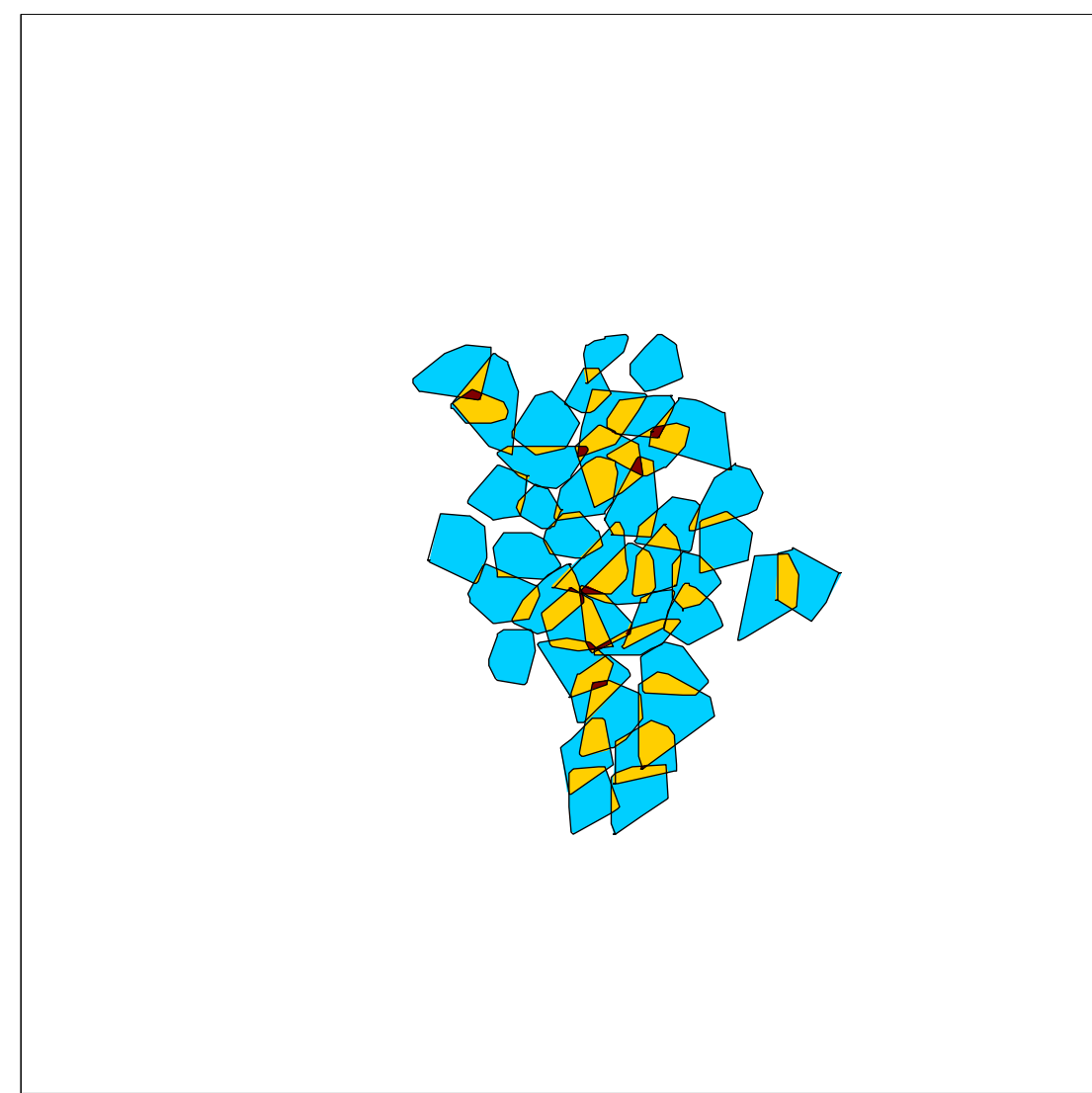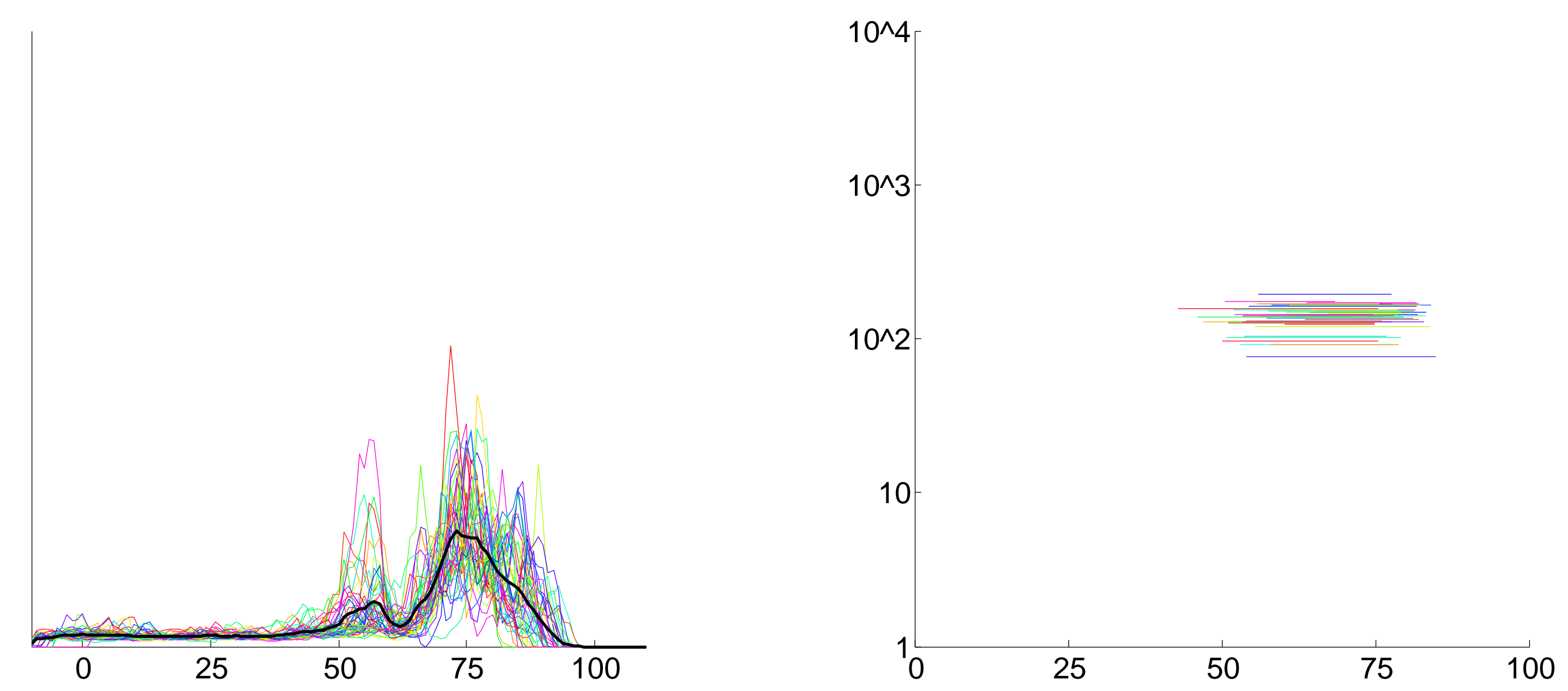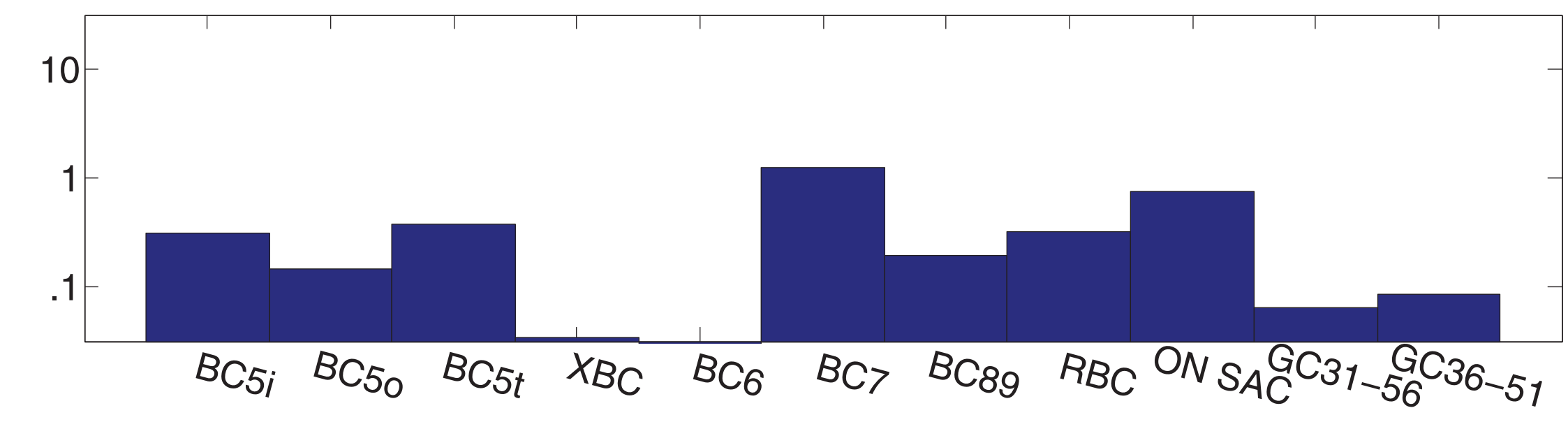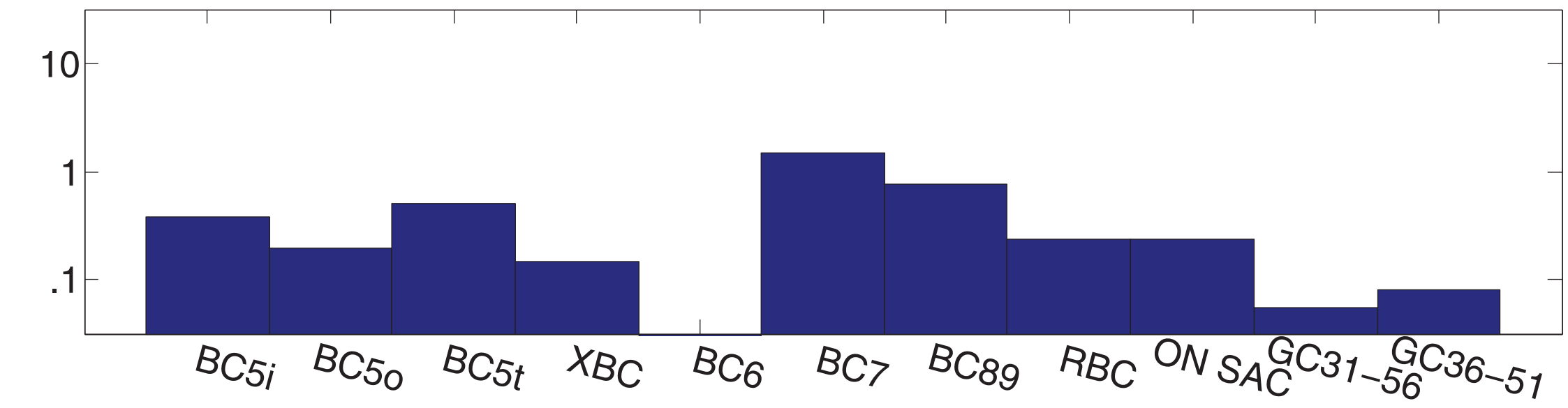

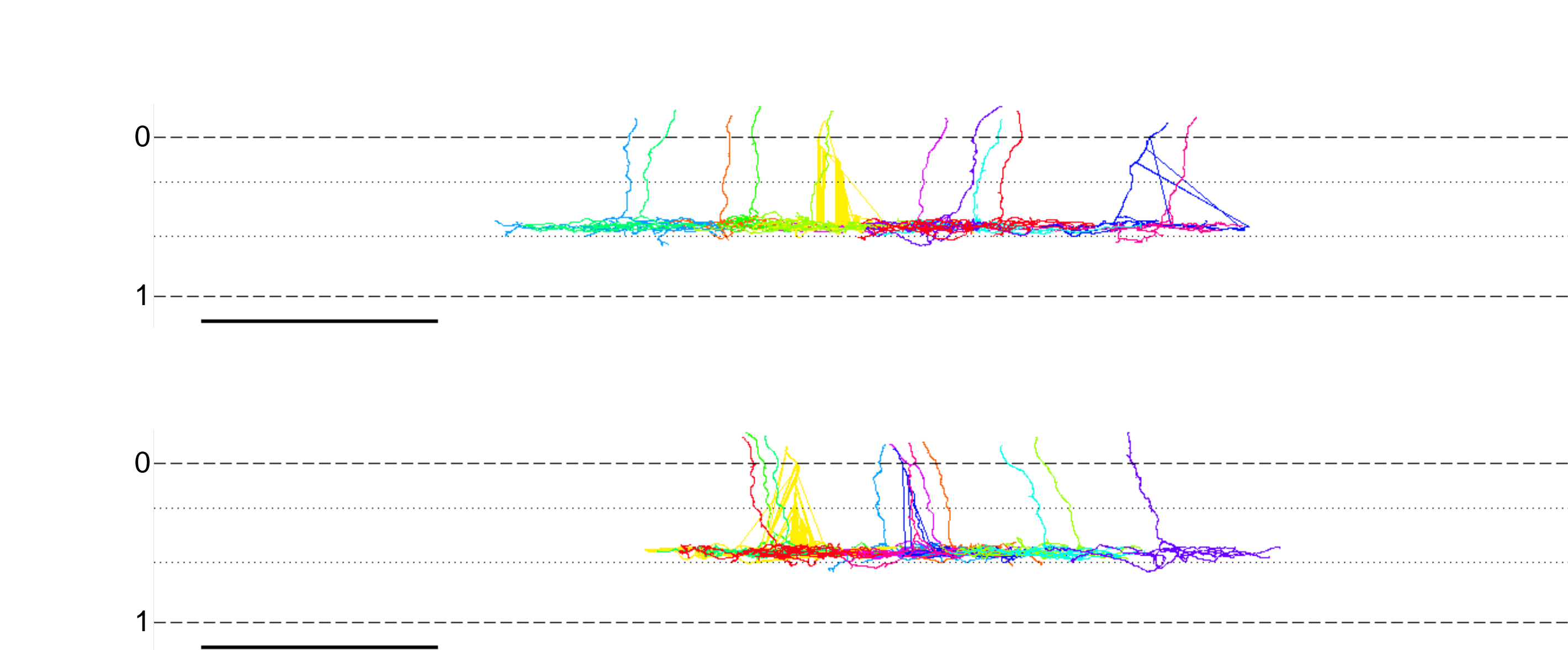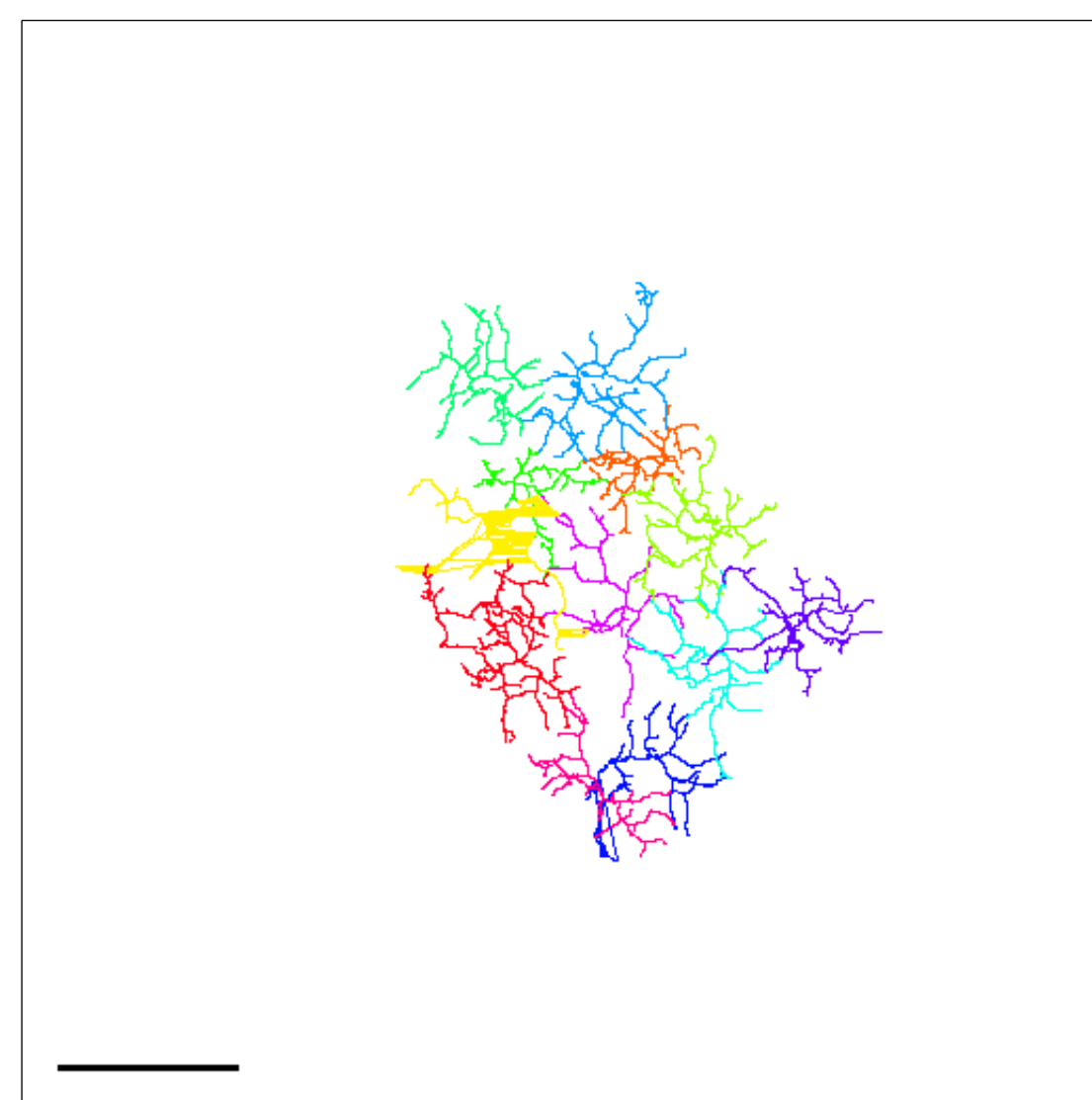

XBC

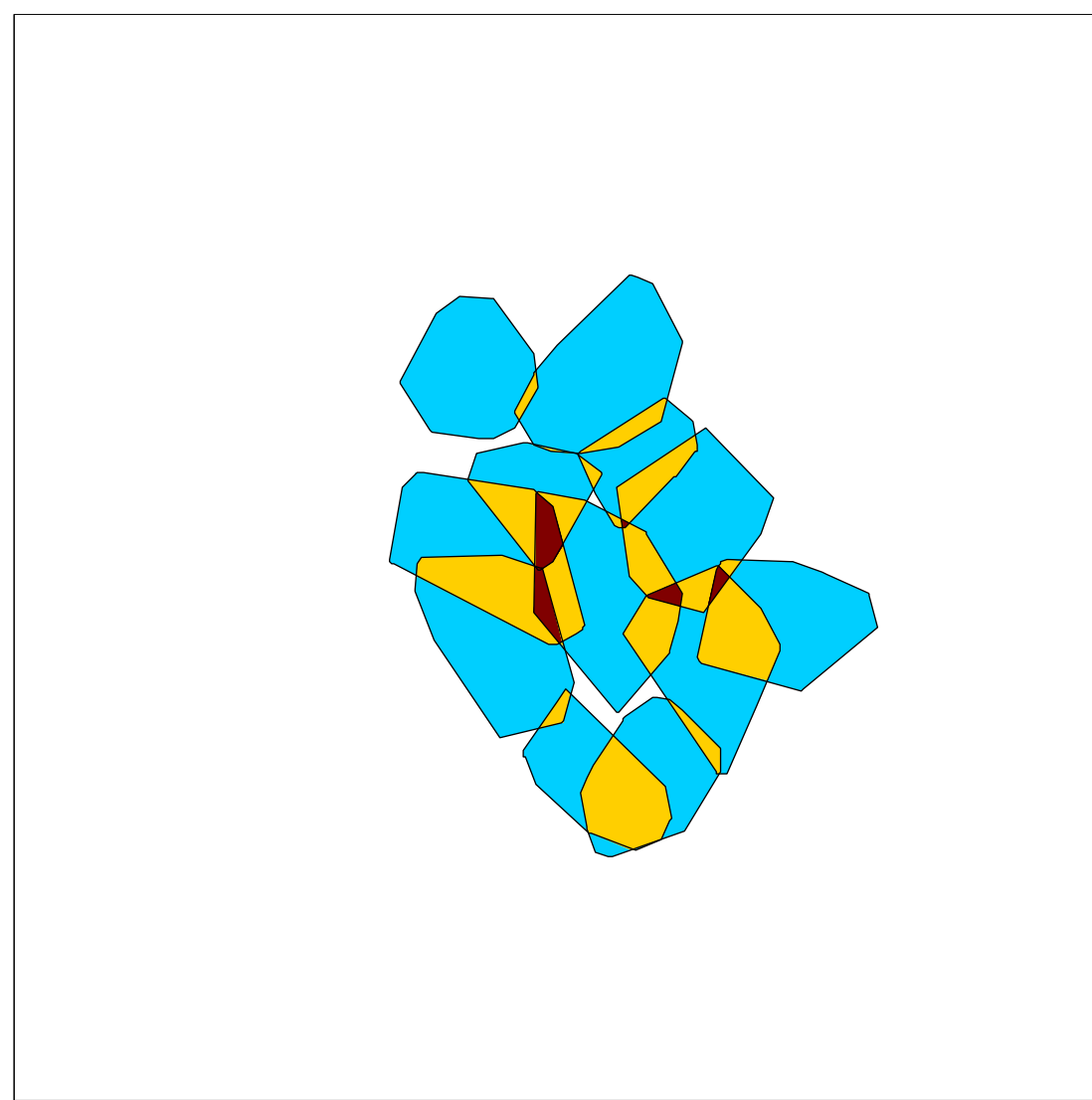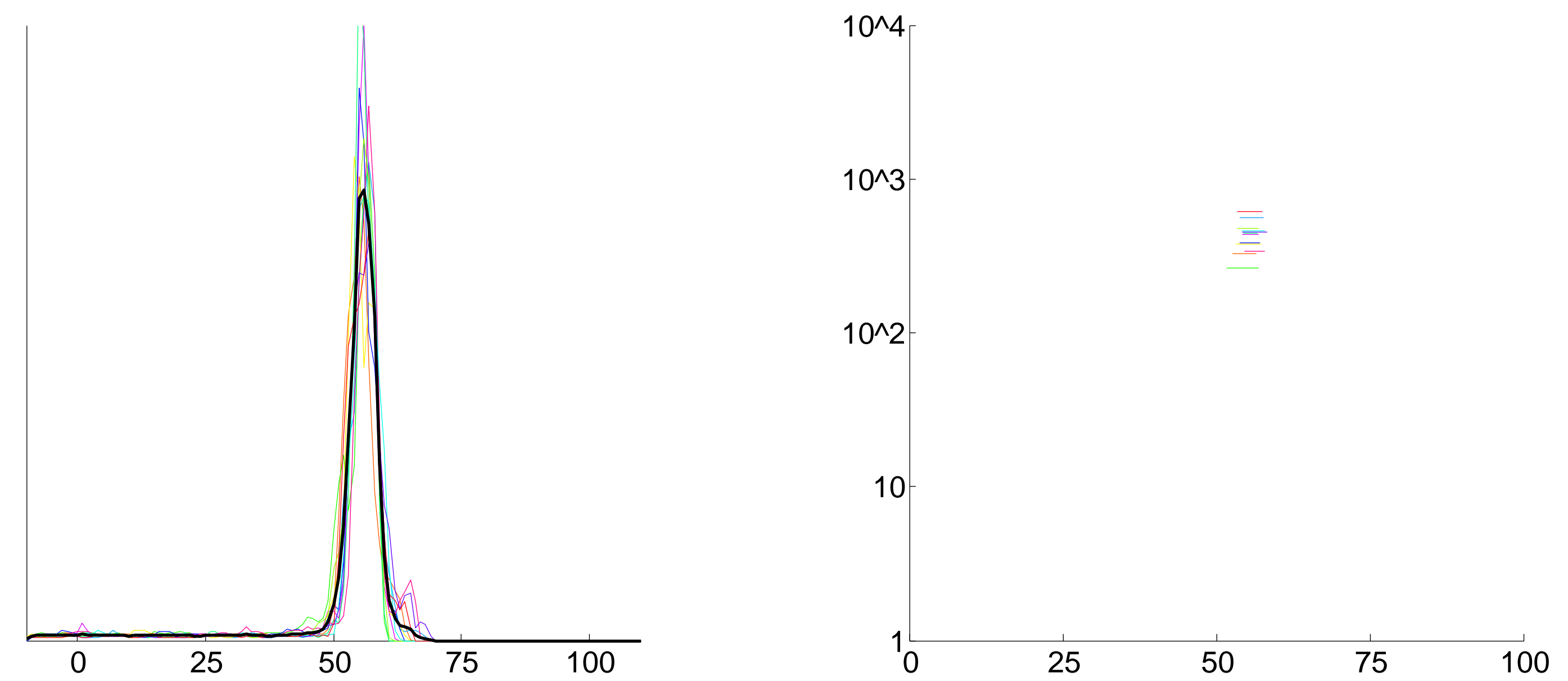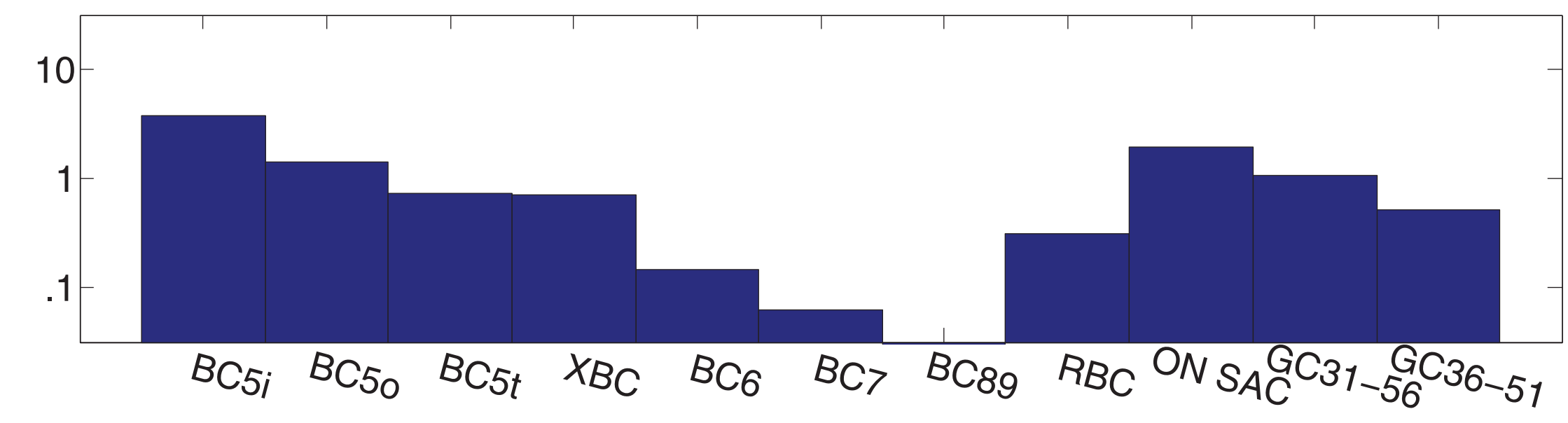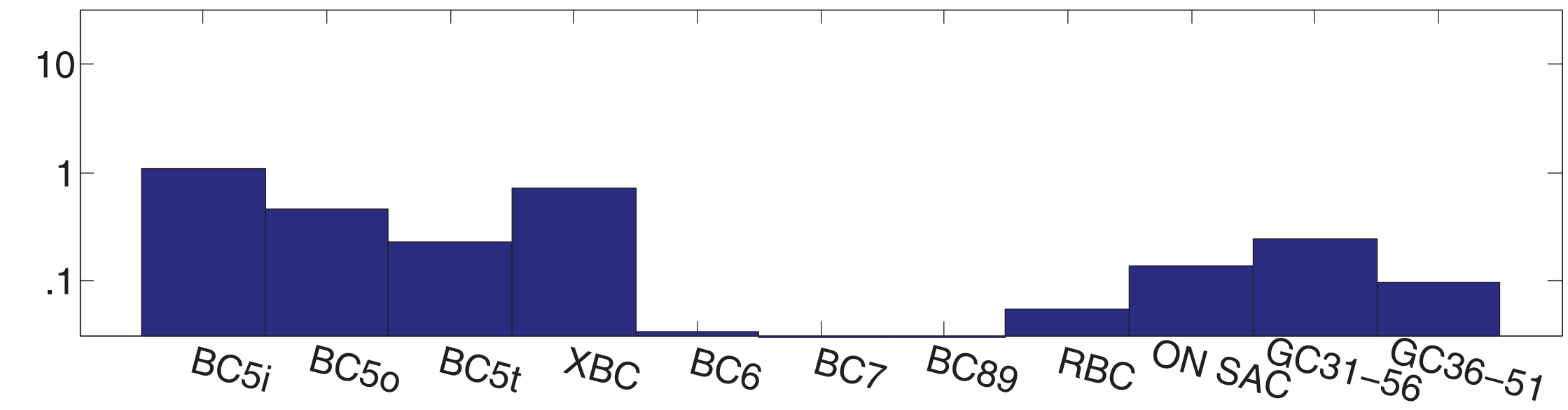

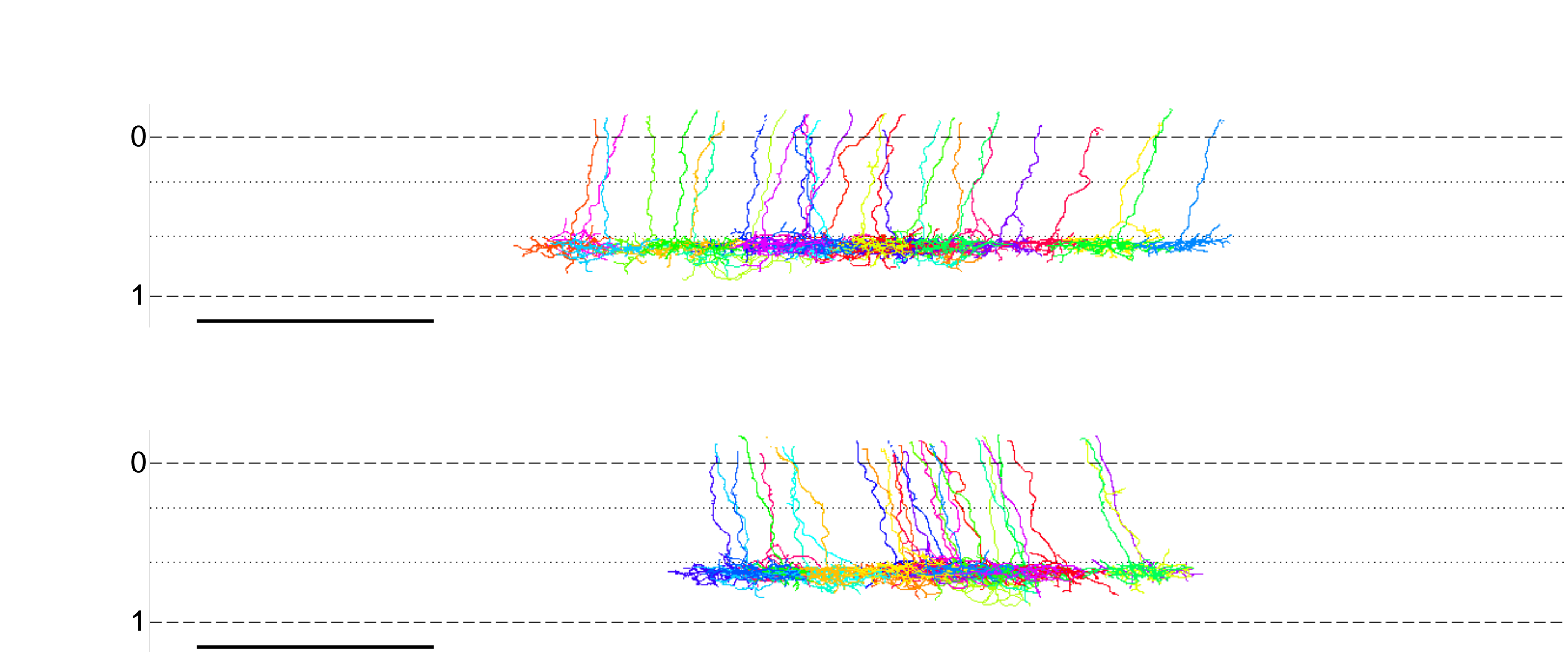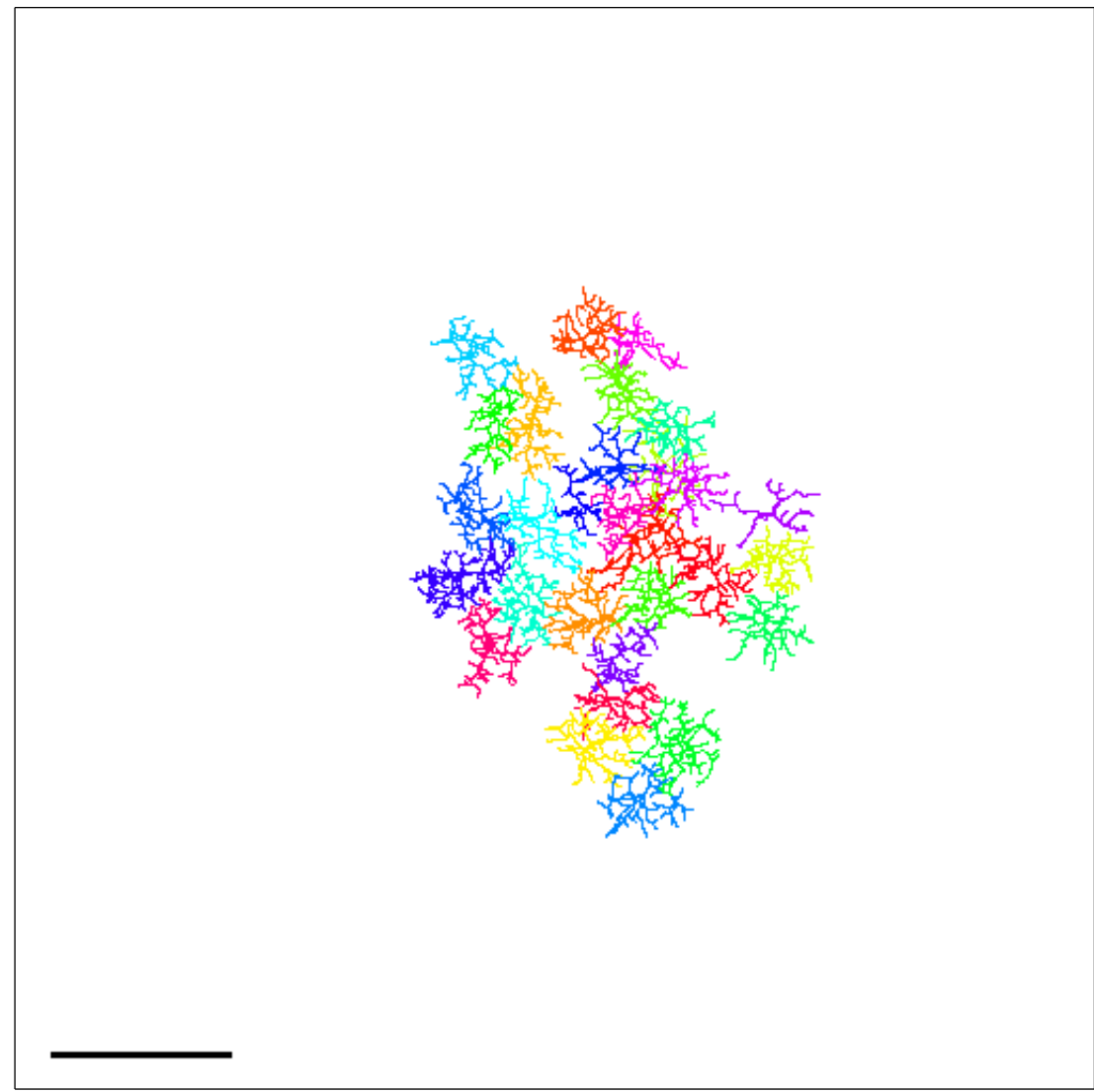

BC7

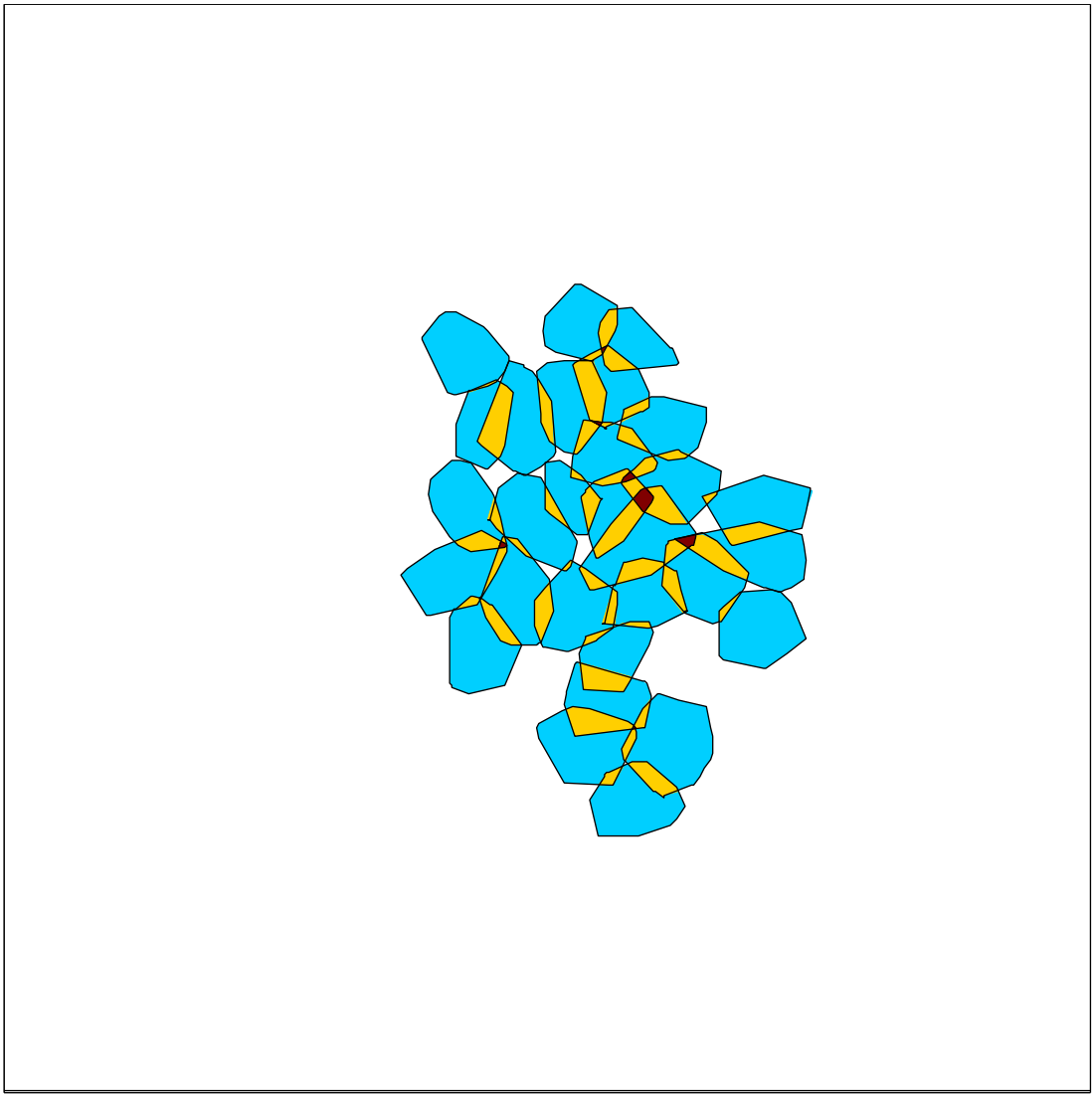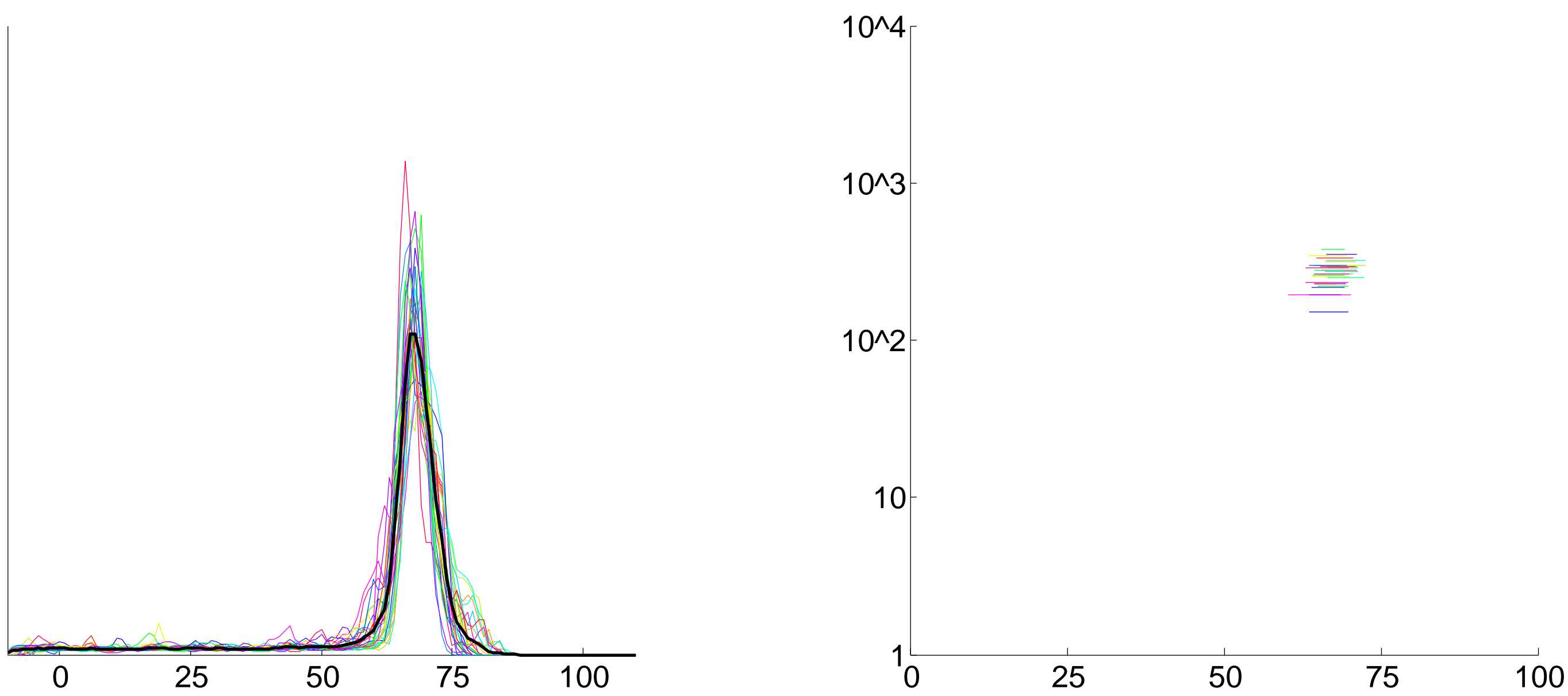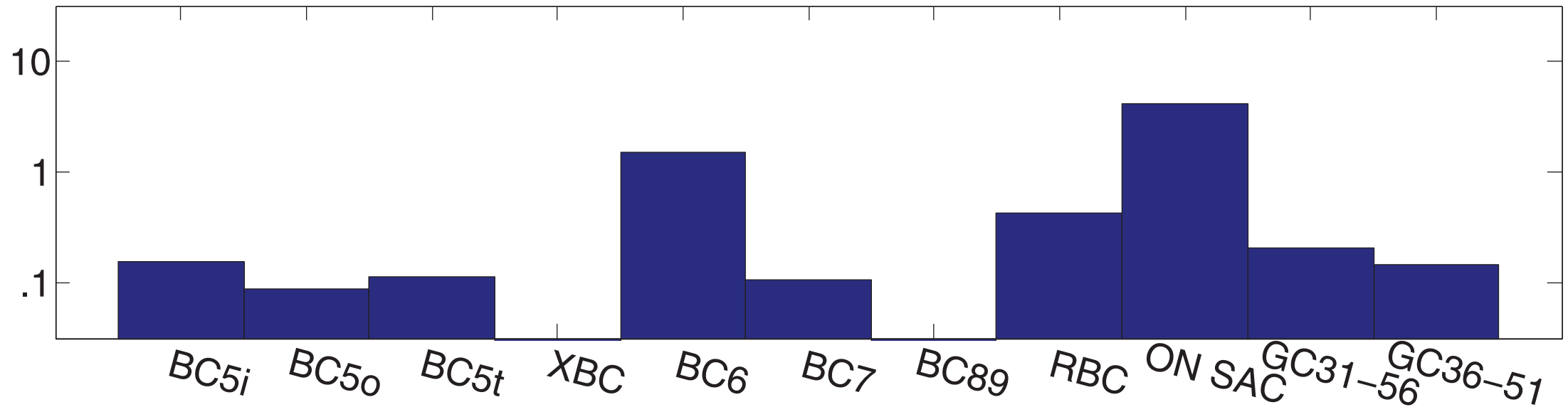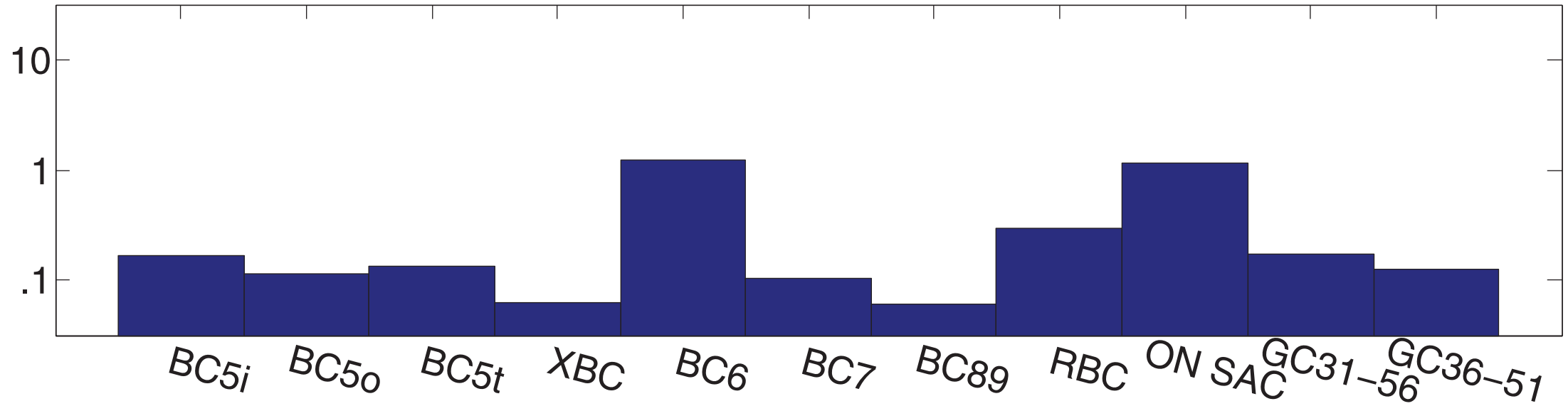

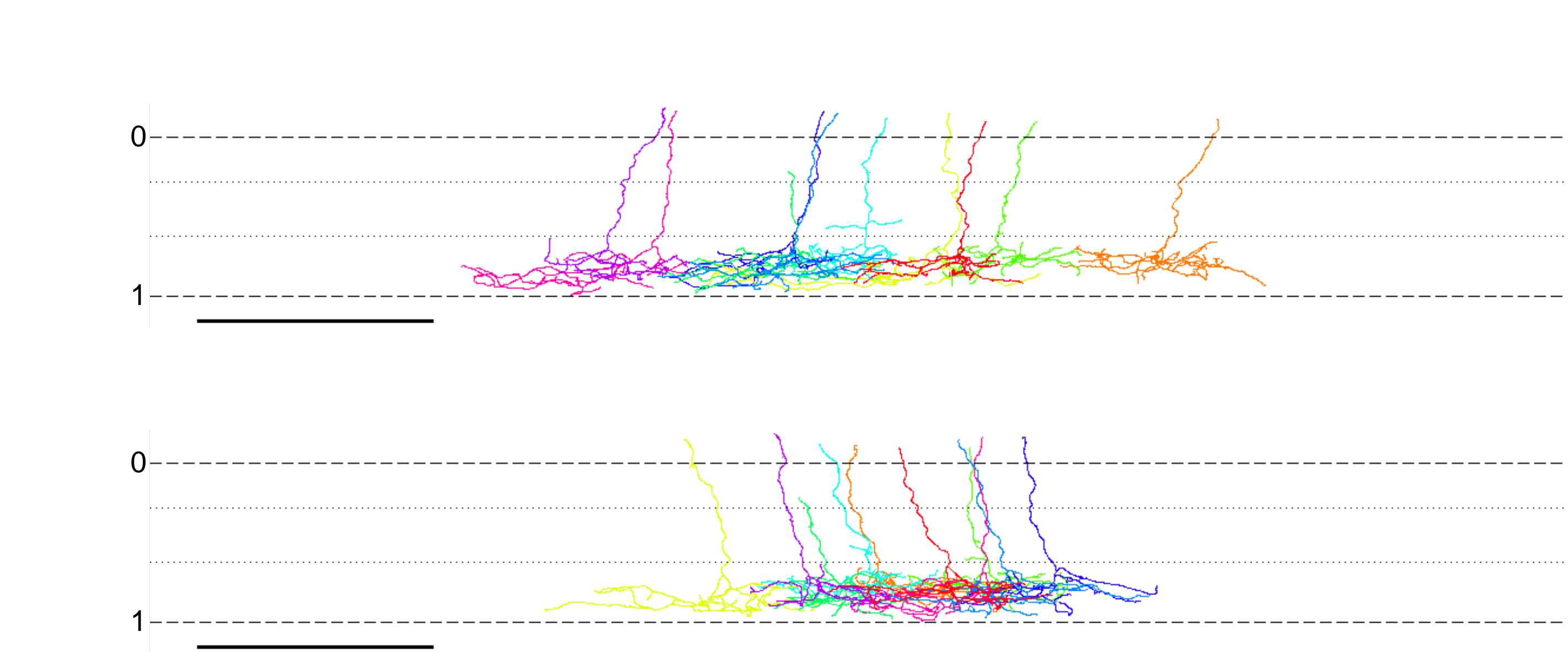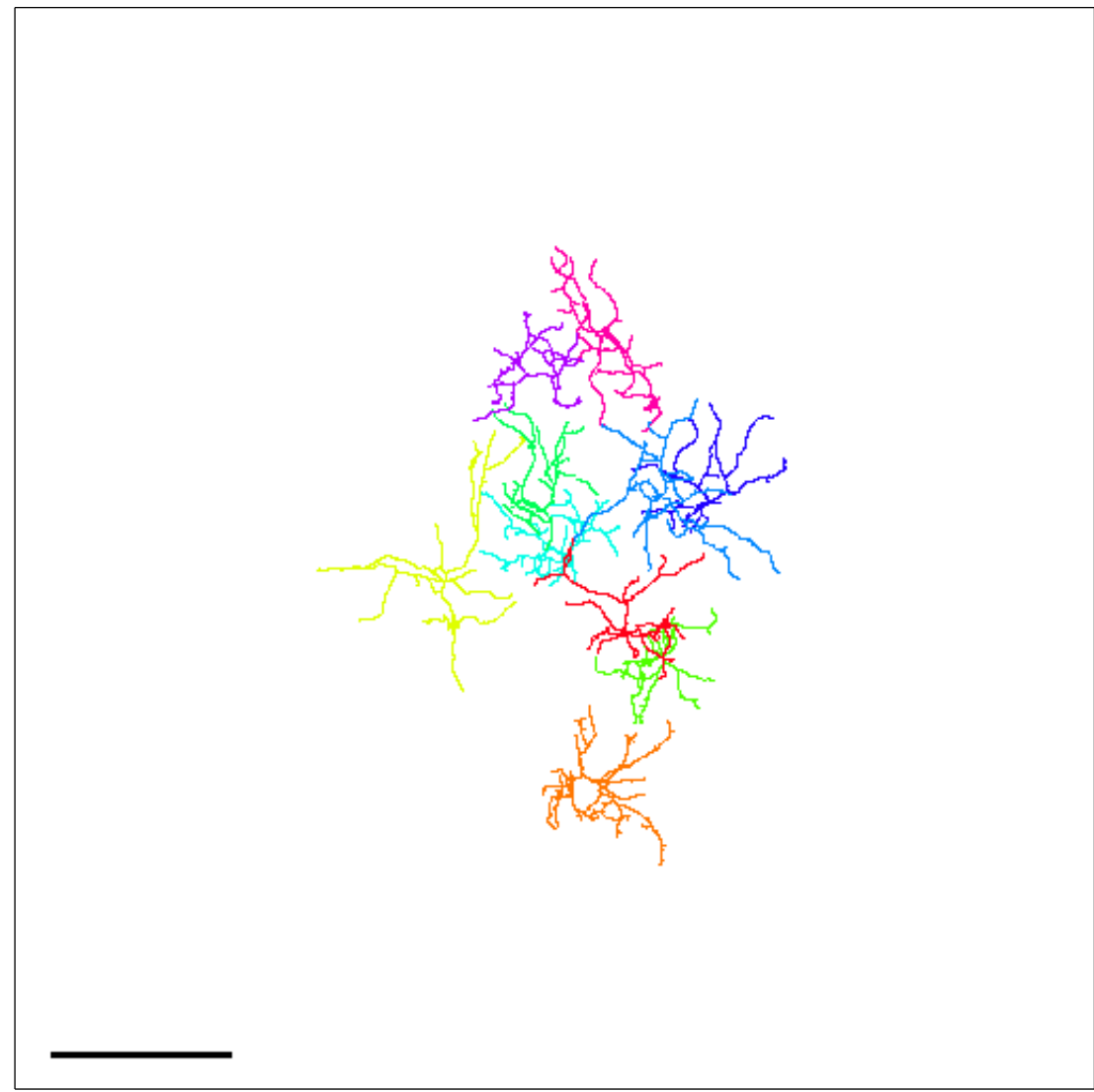

BC89

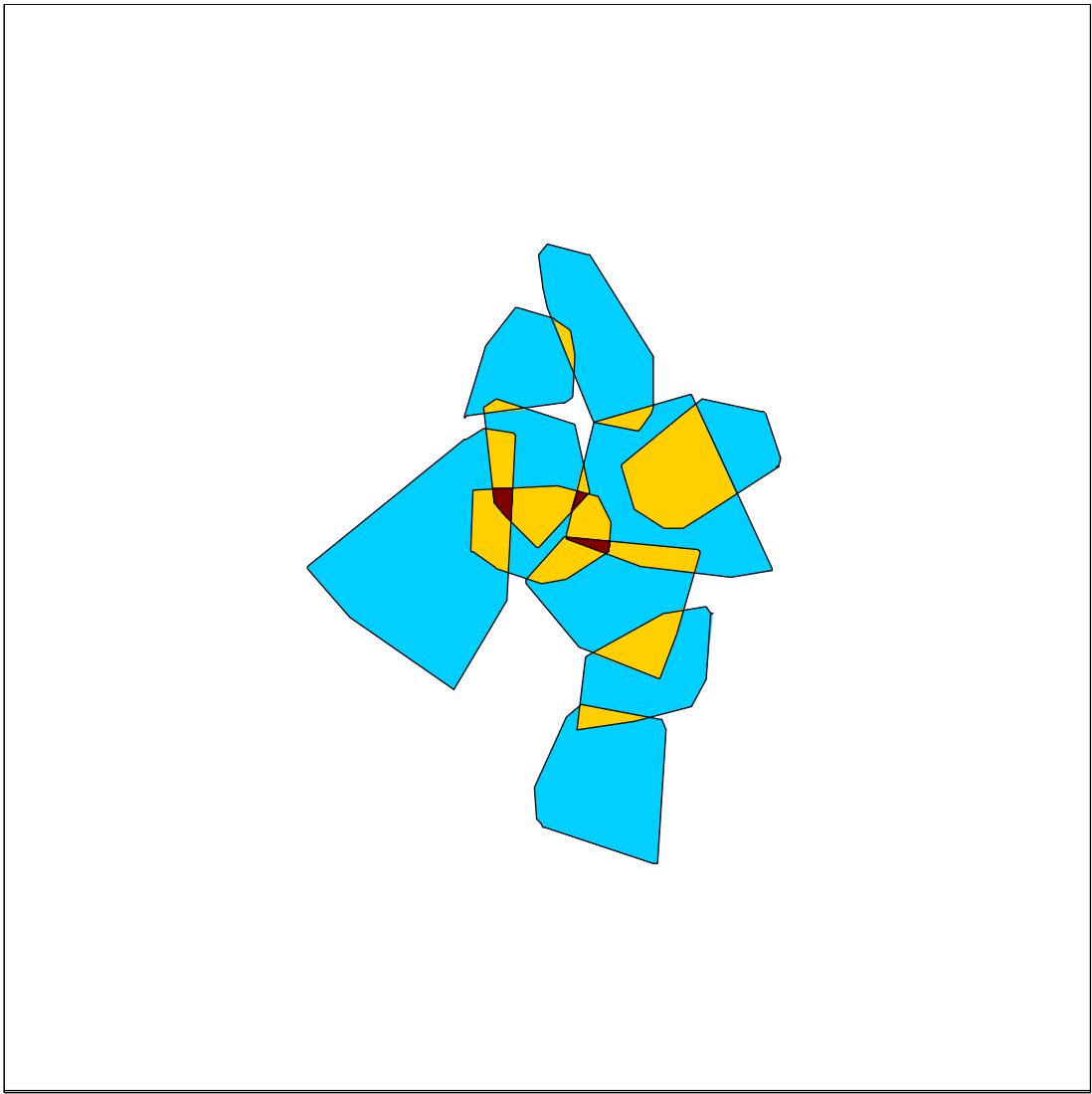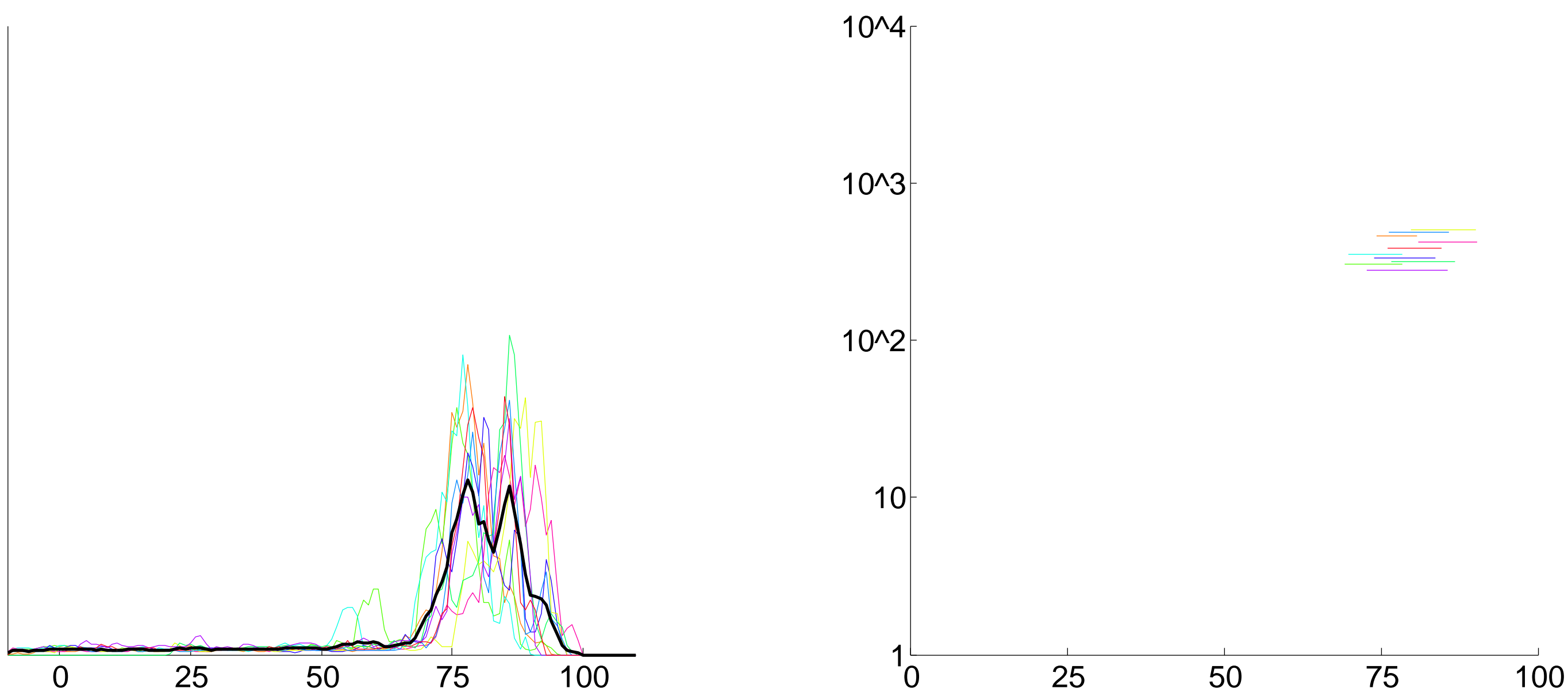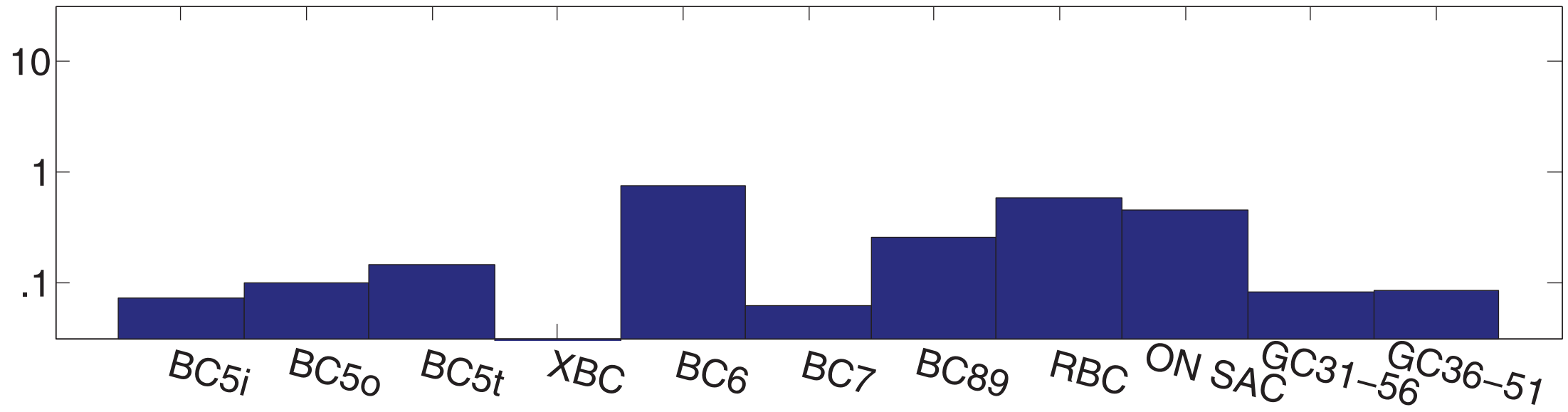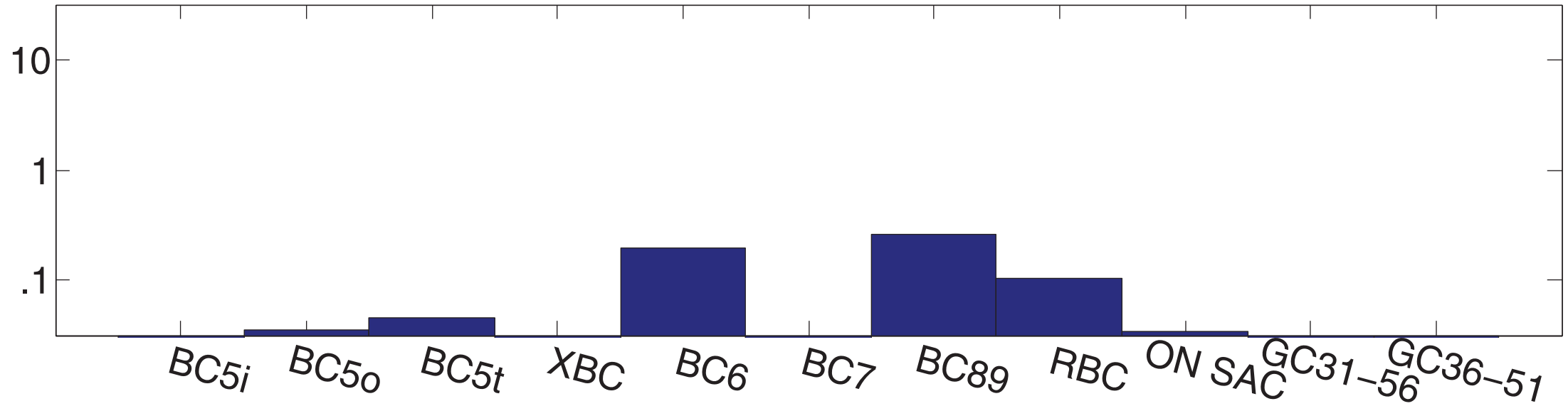

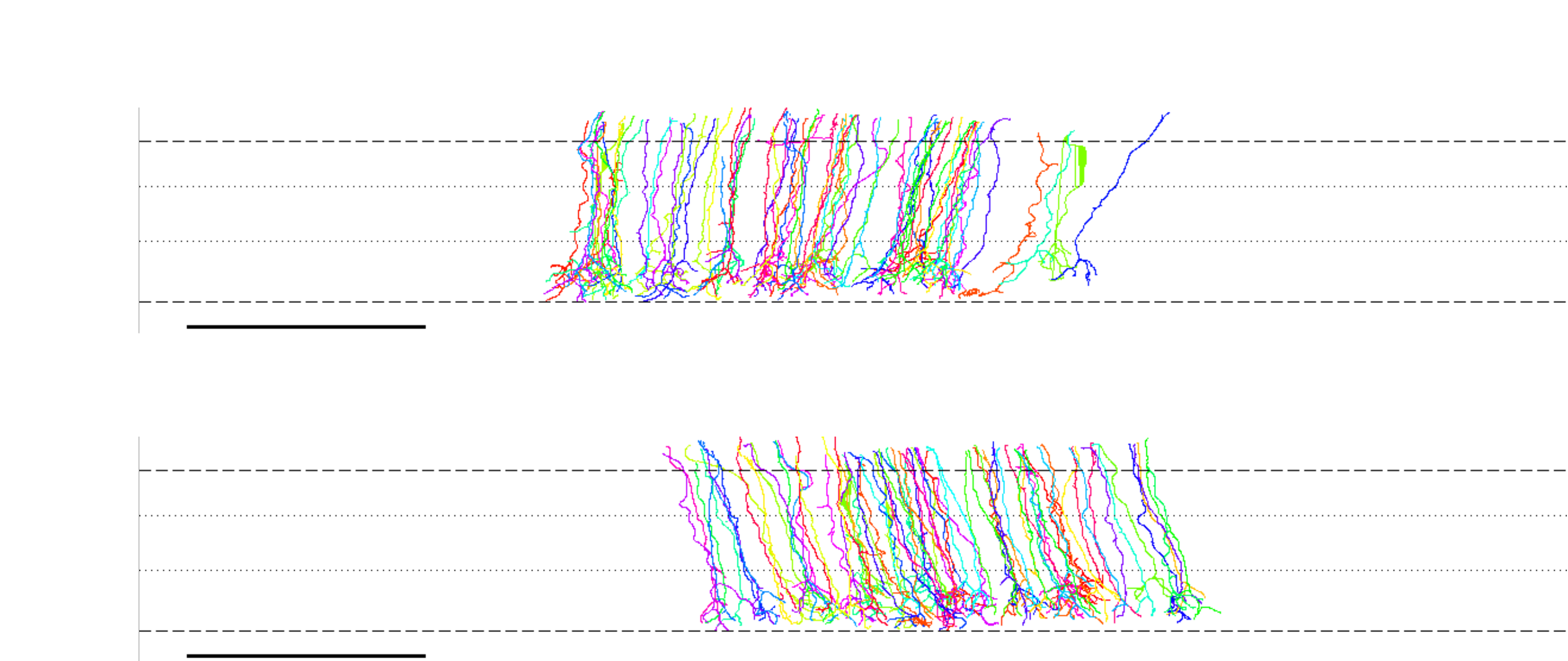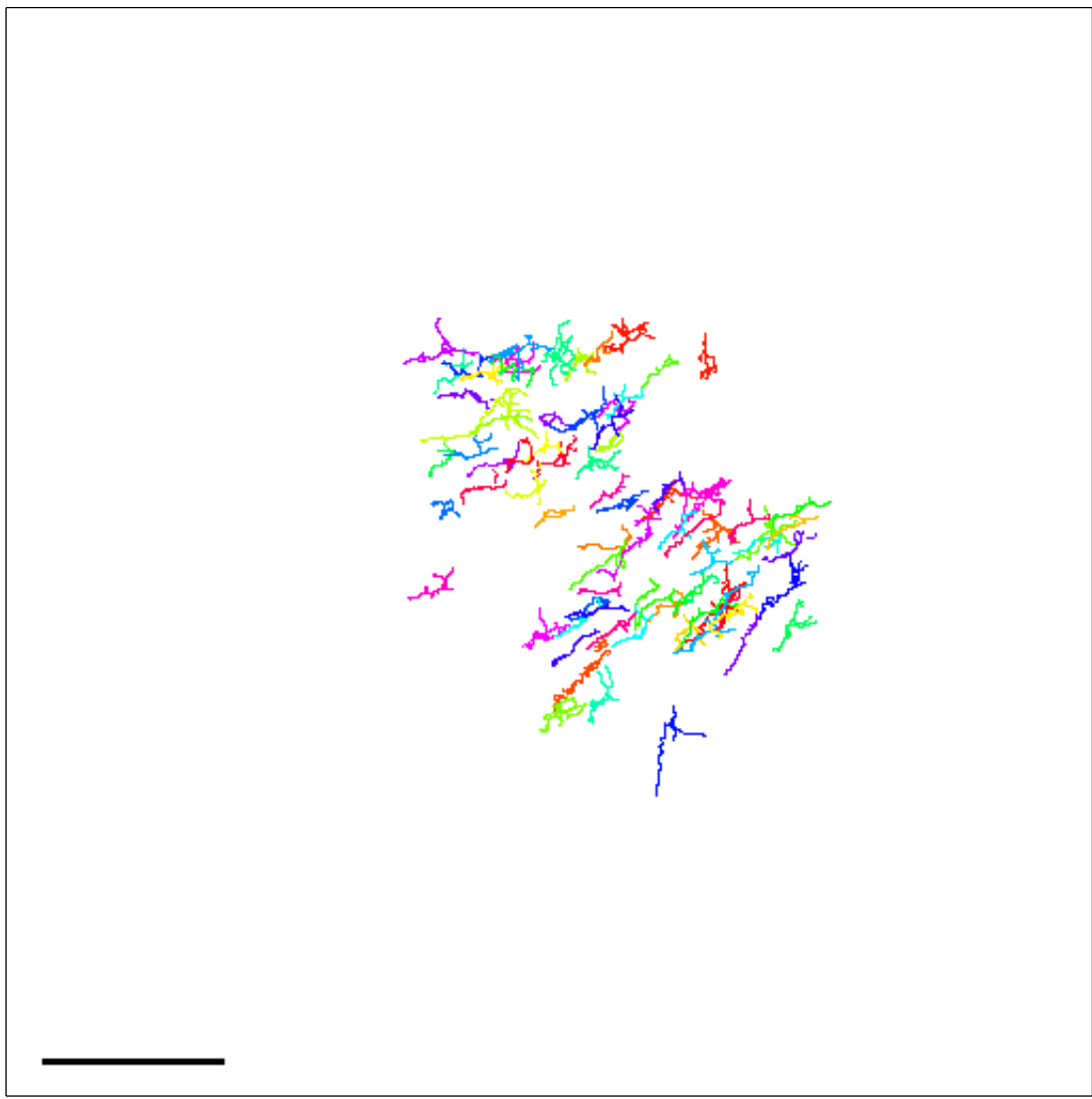

RBC

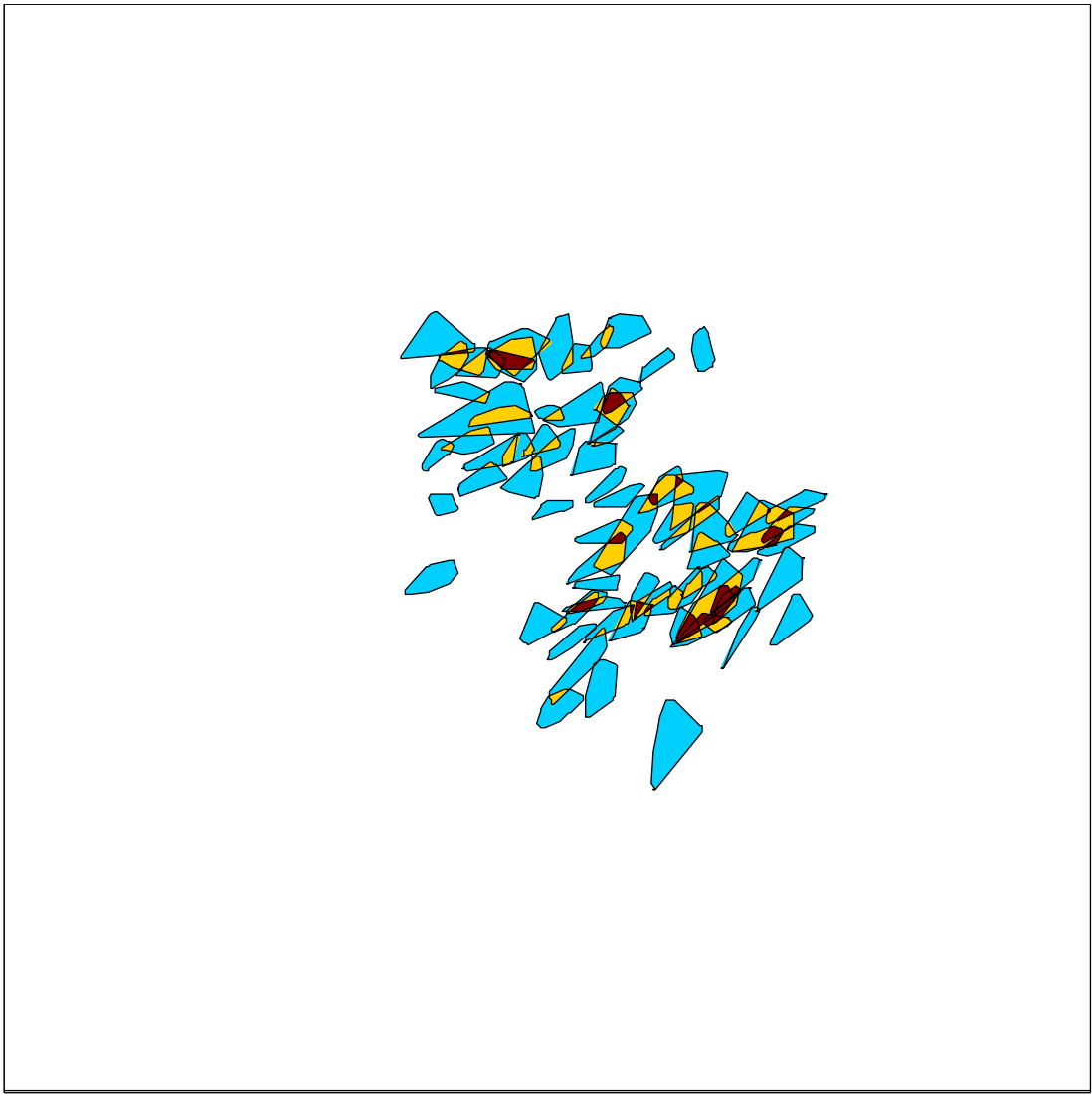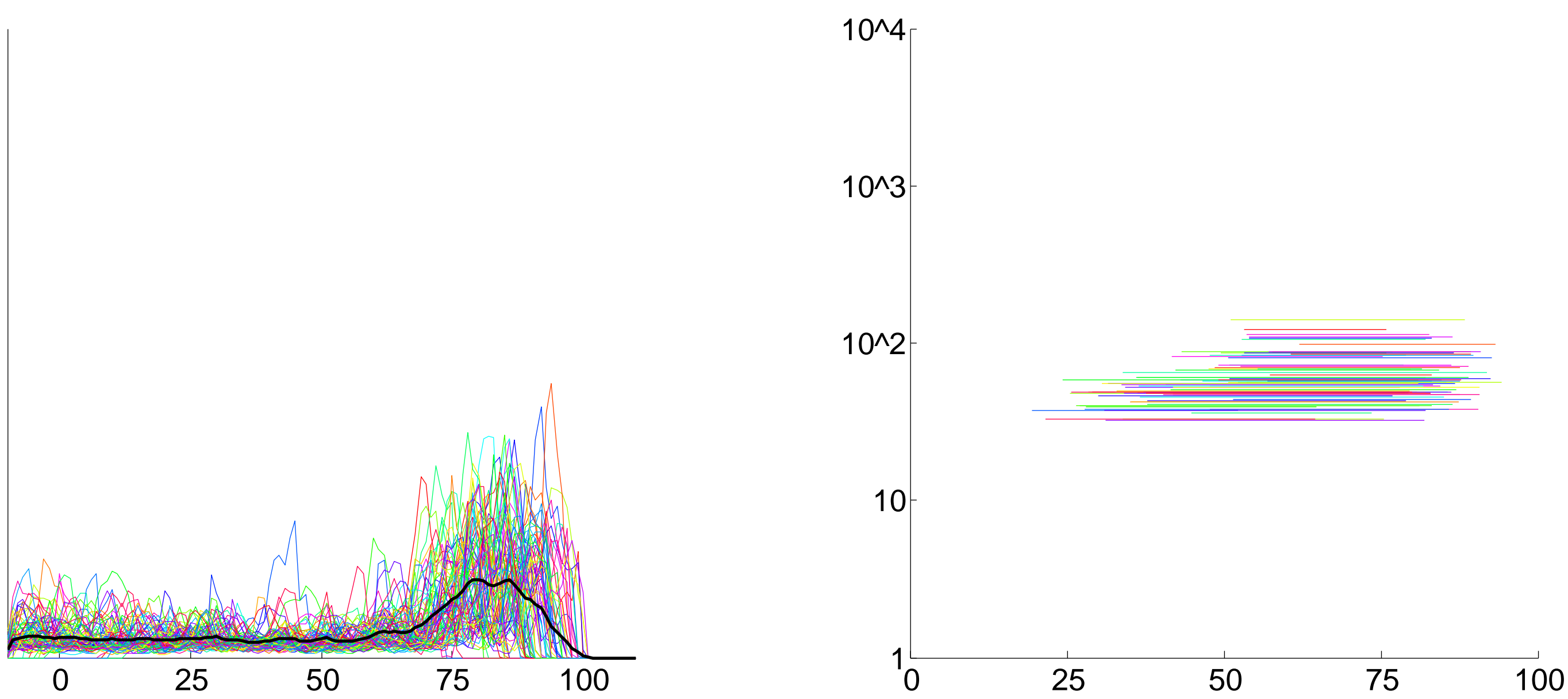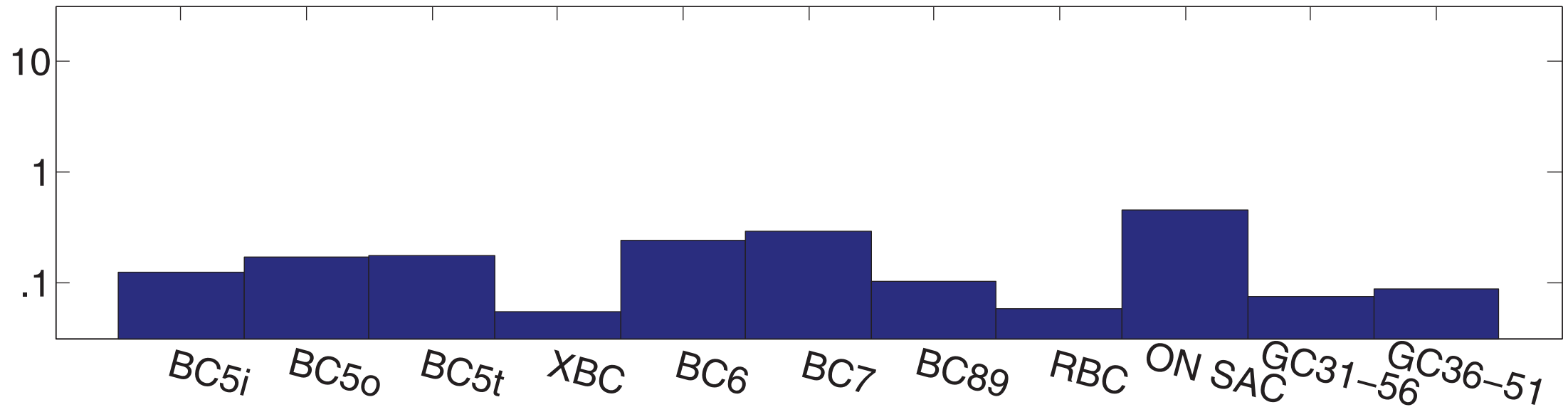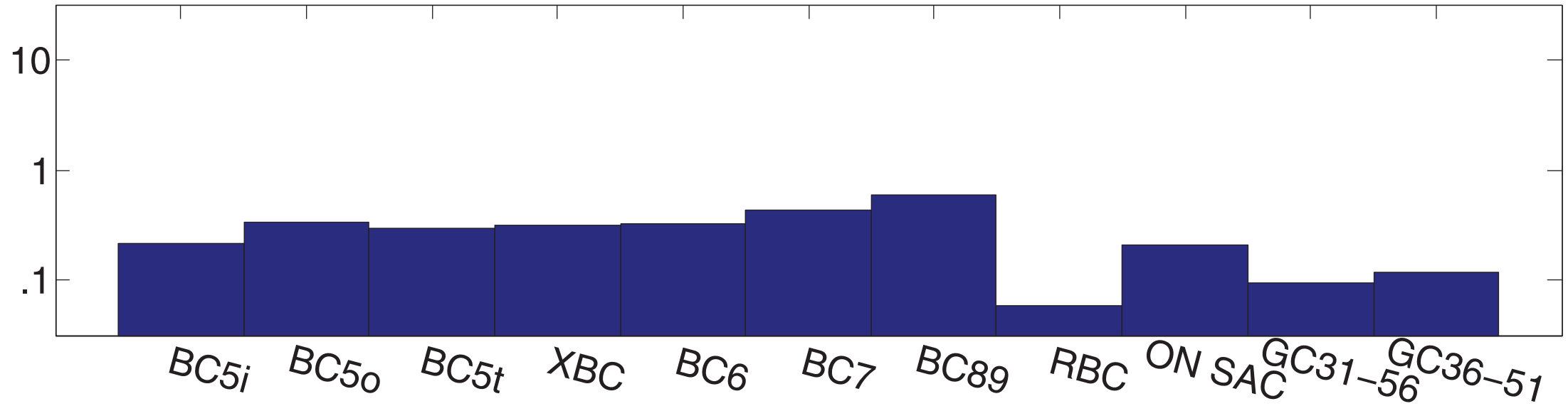

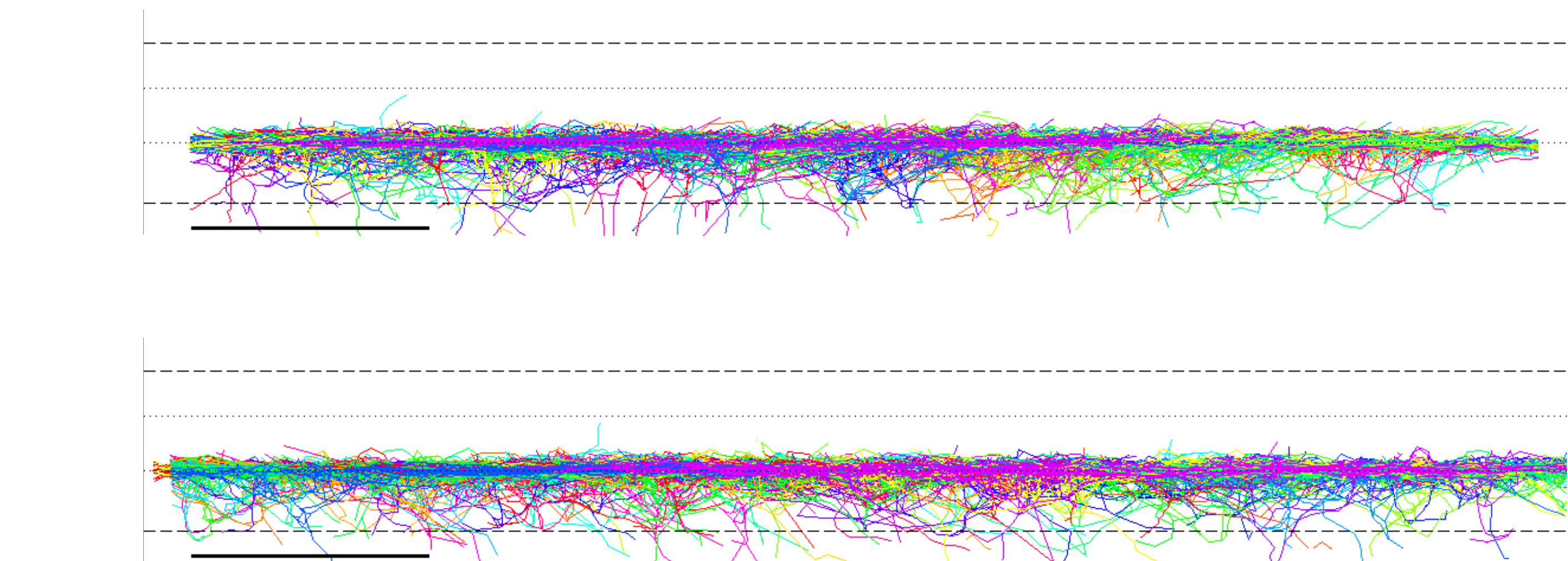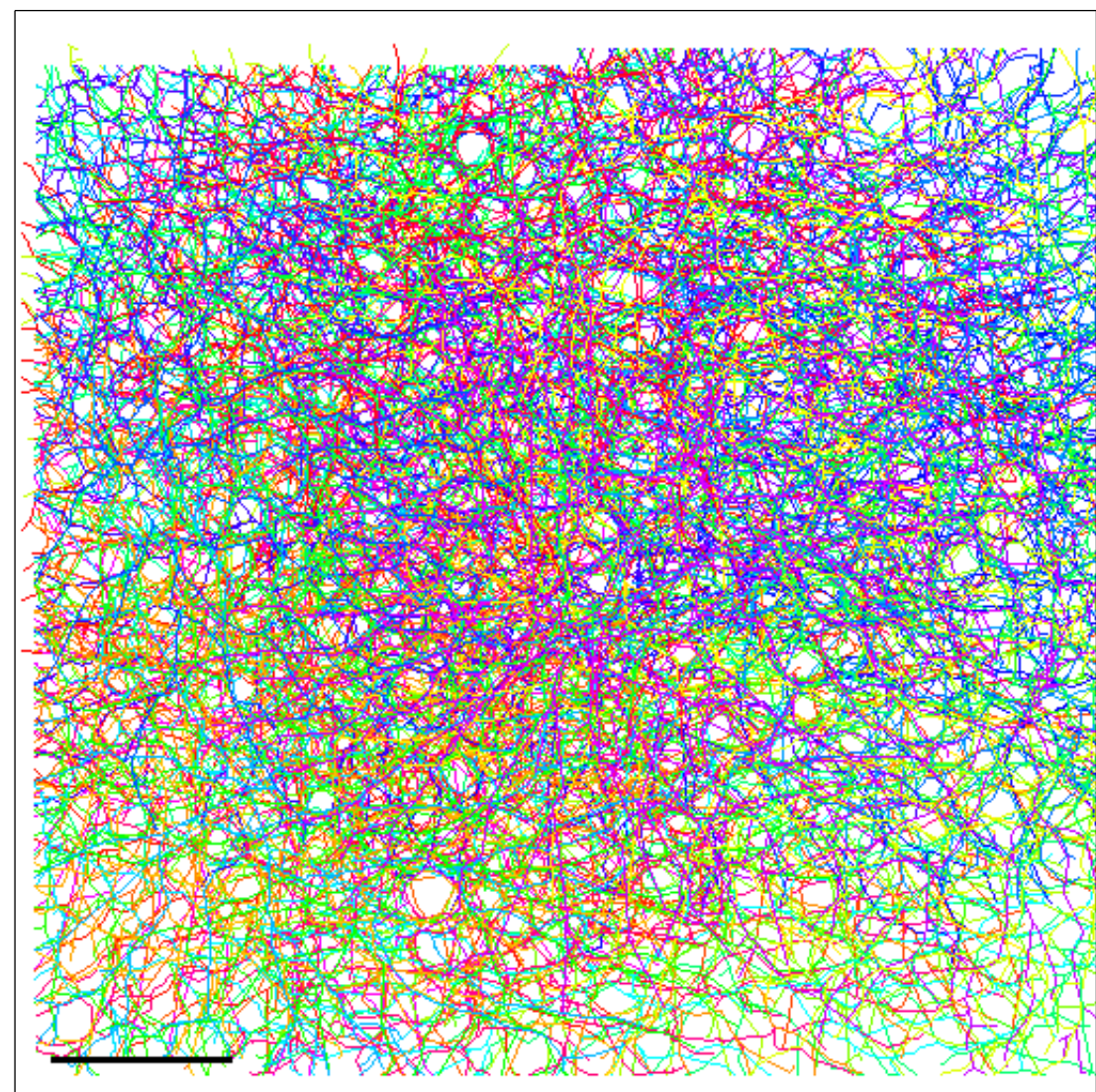

ON SAC

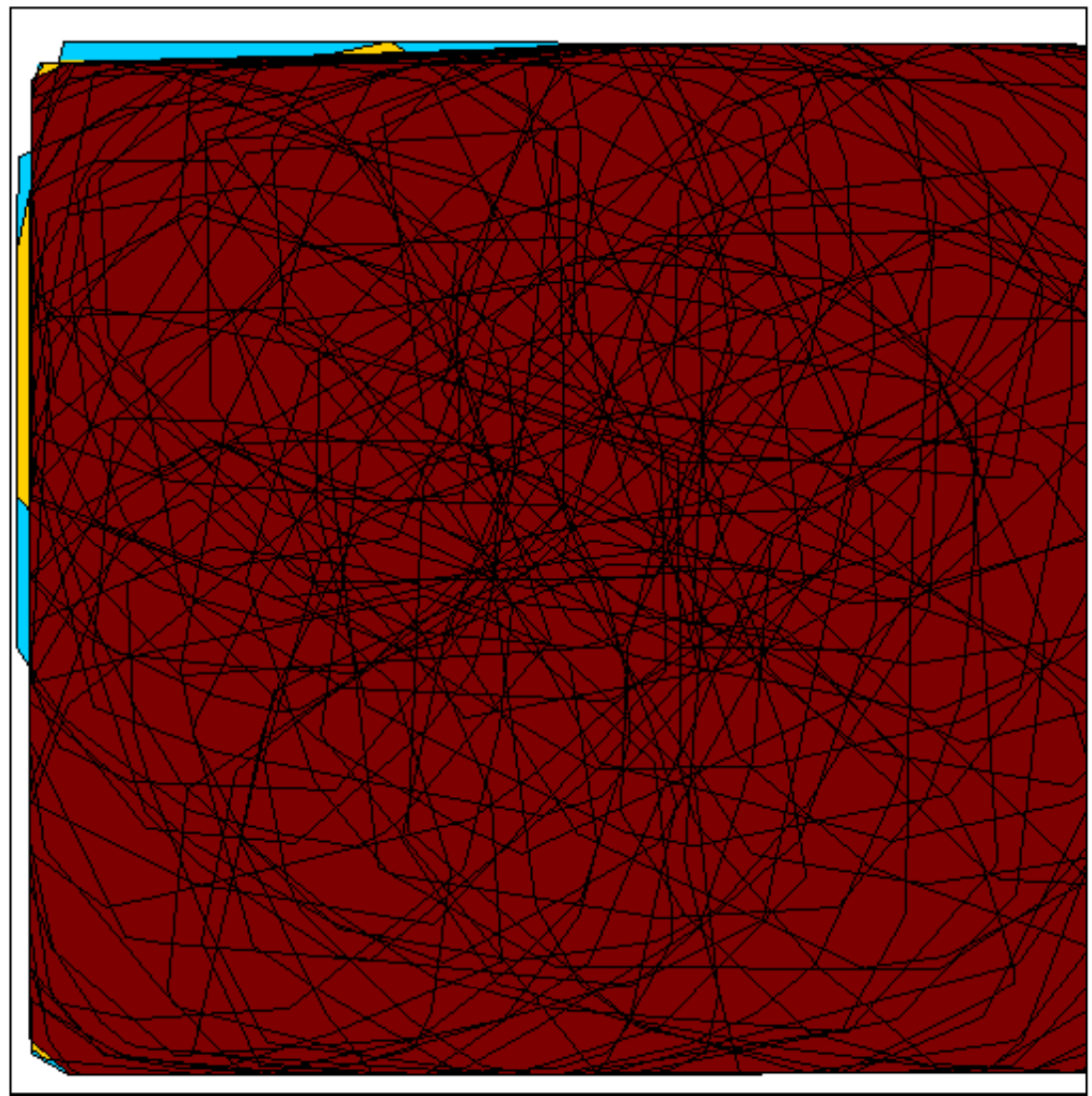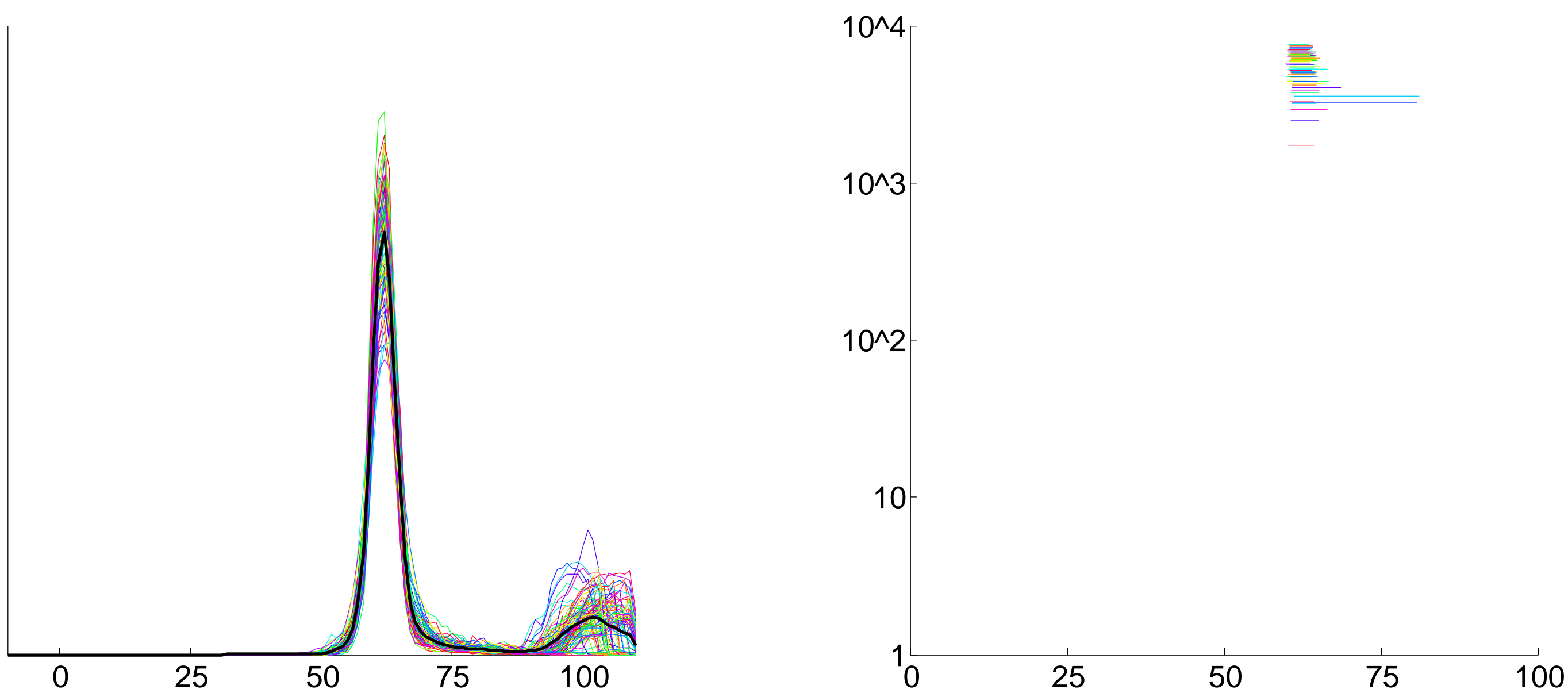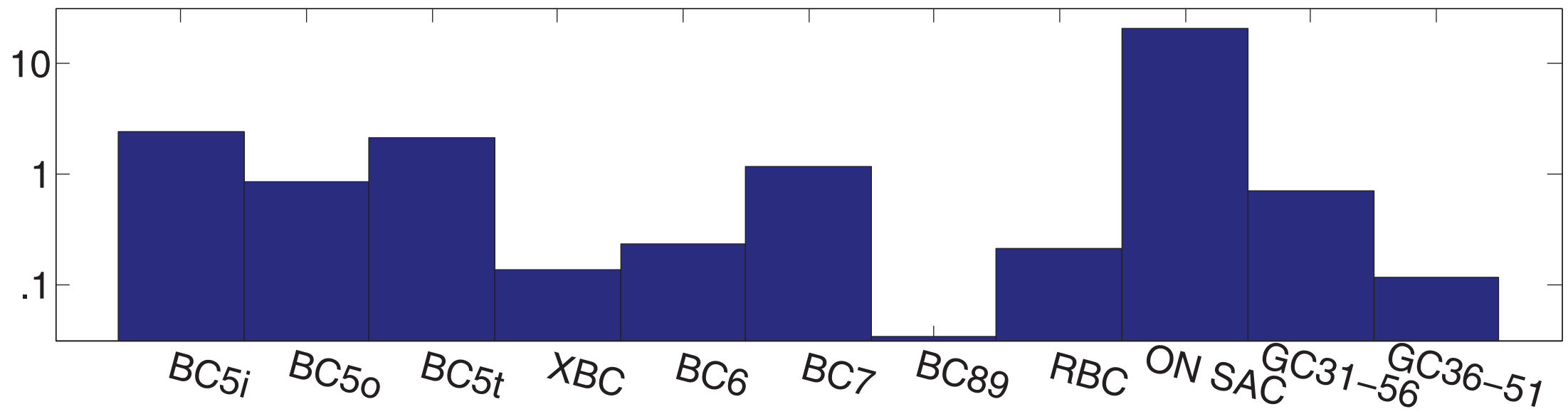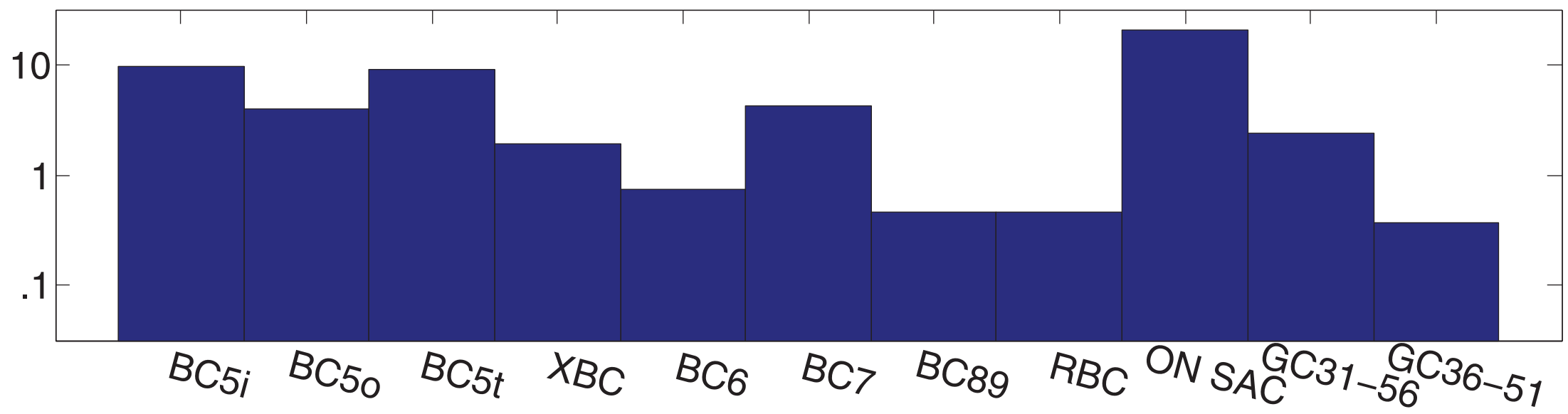

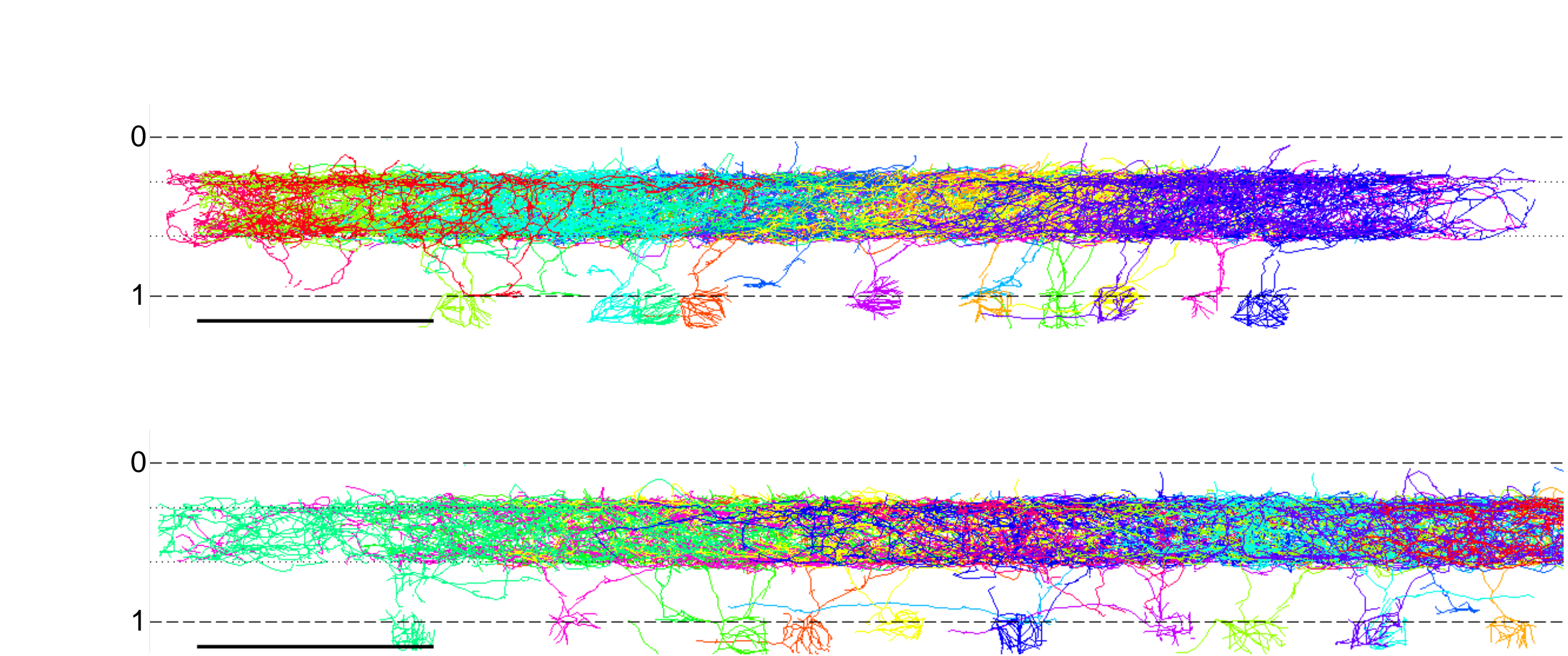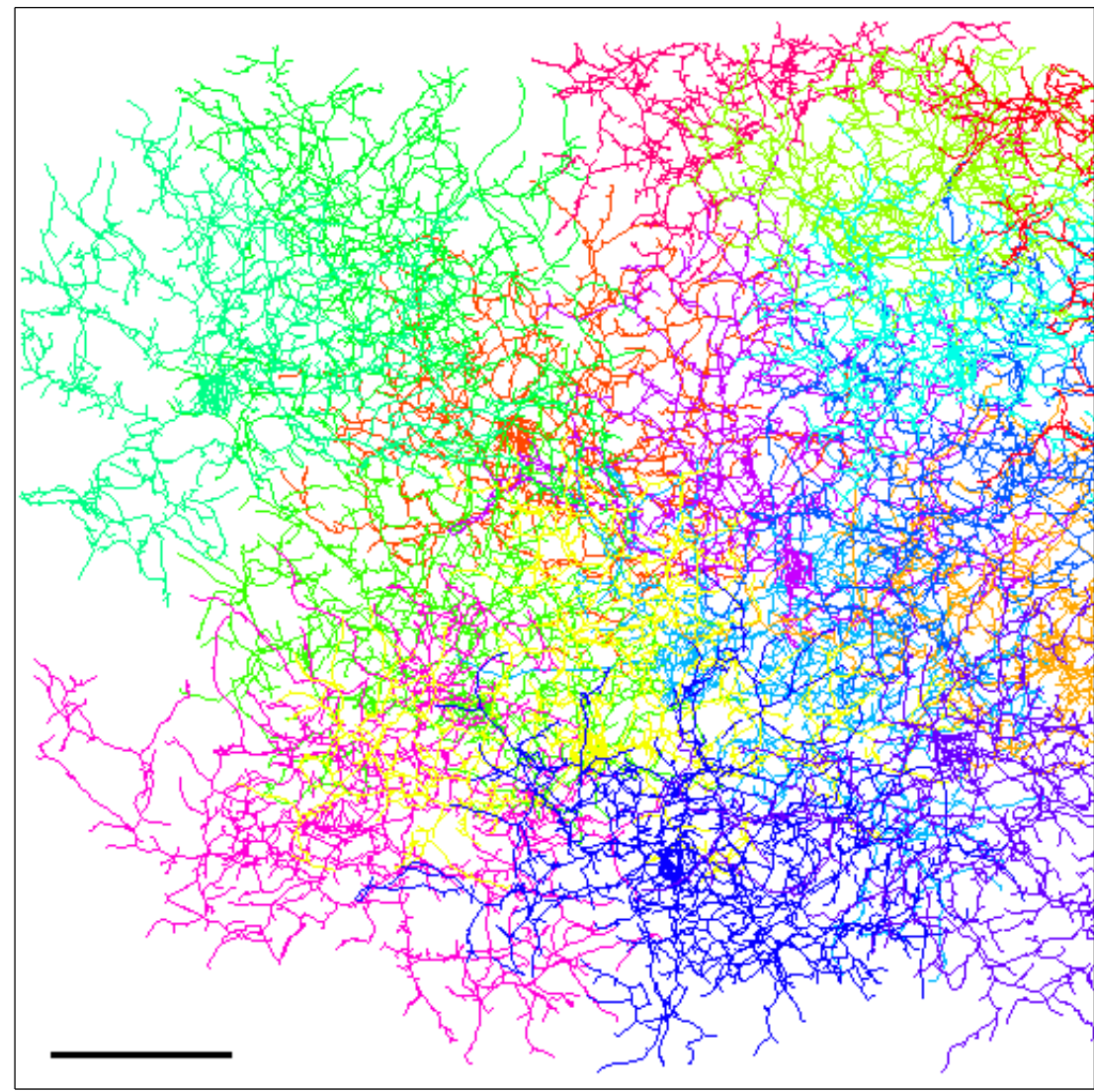

GC31-56

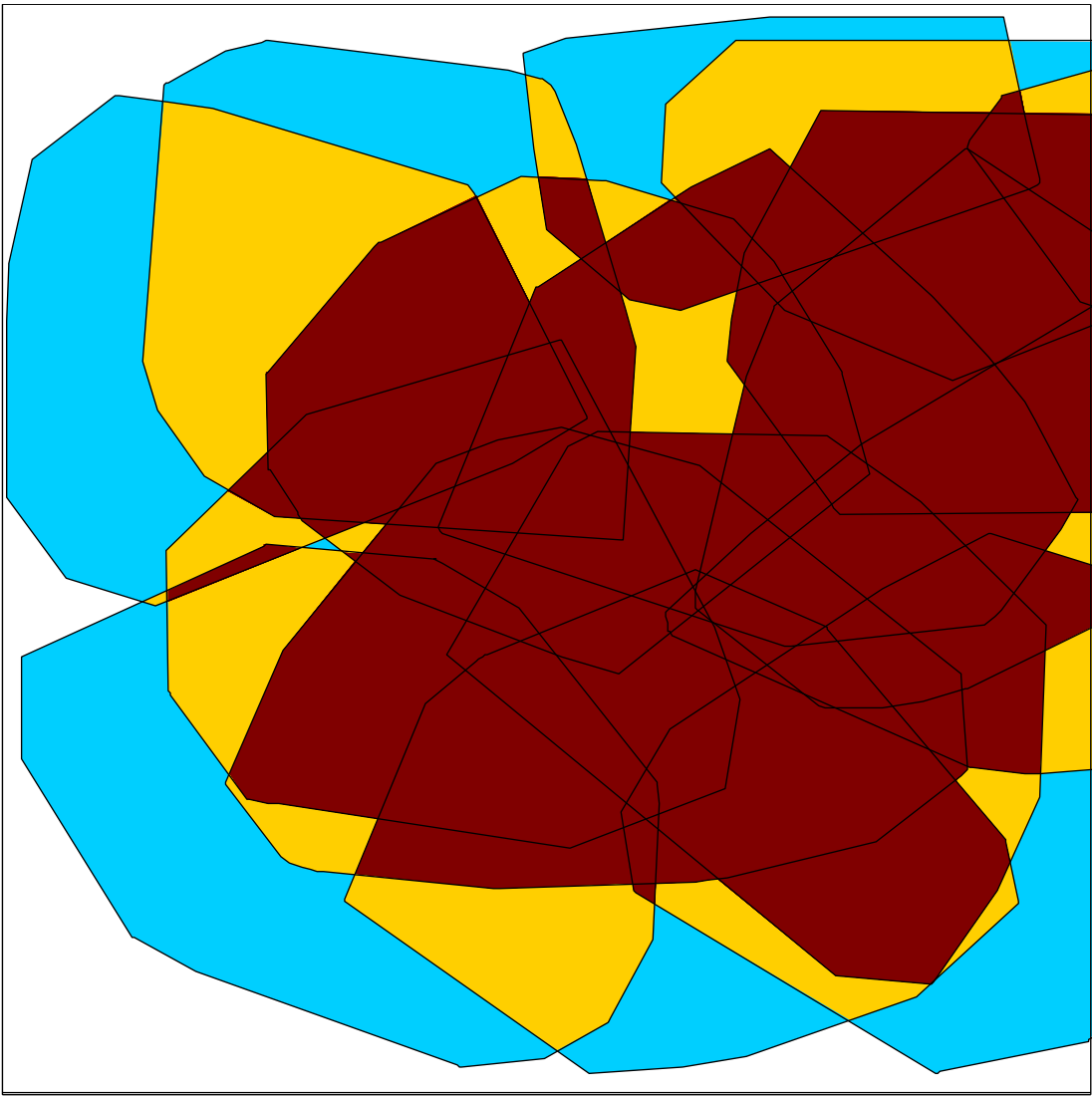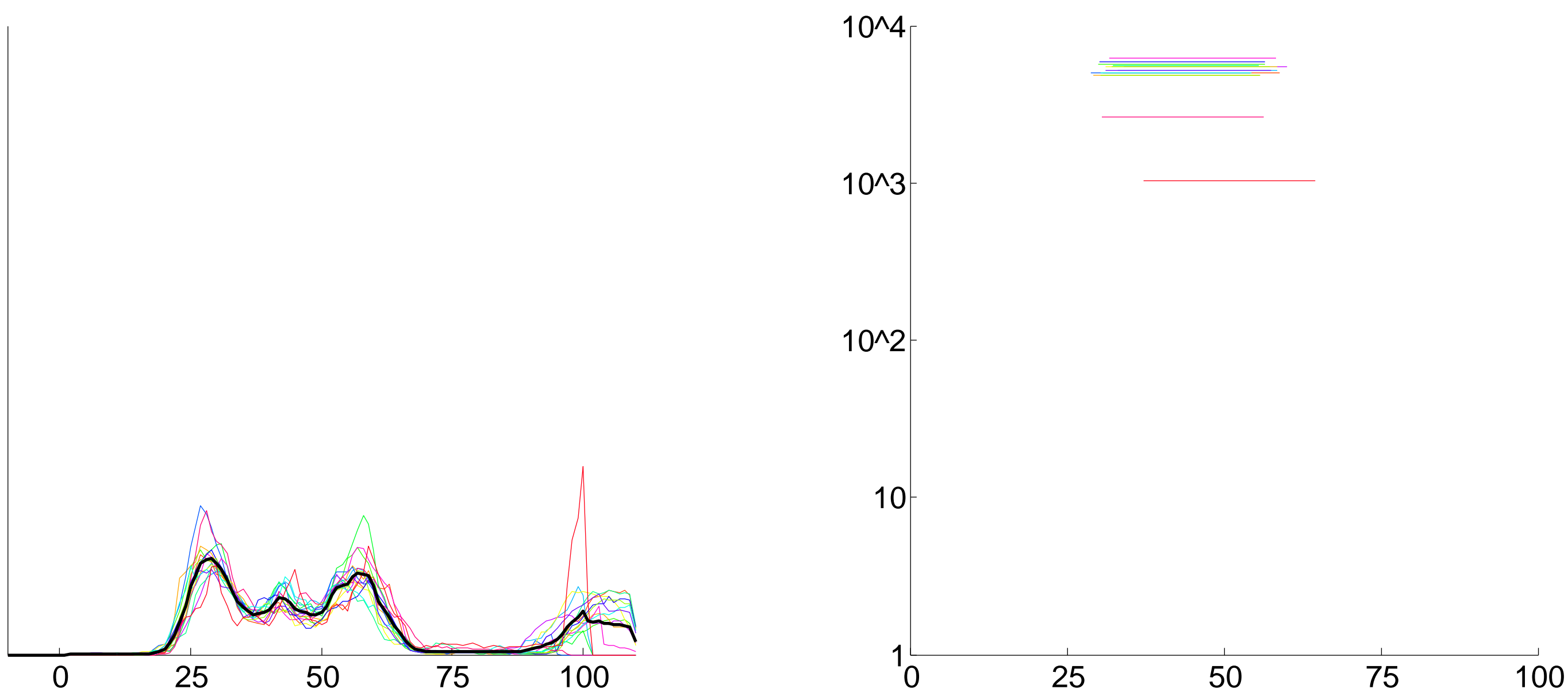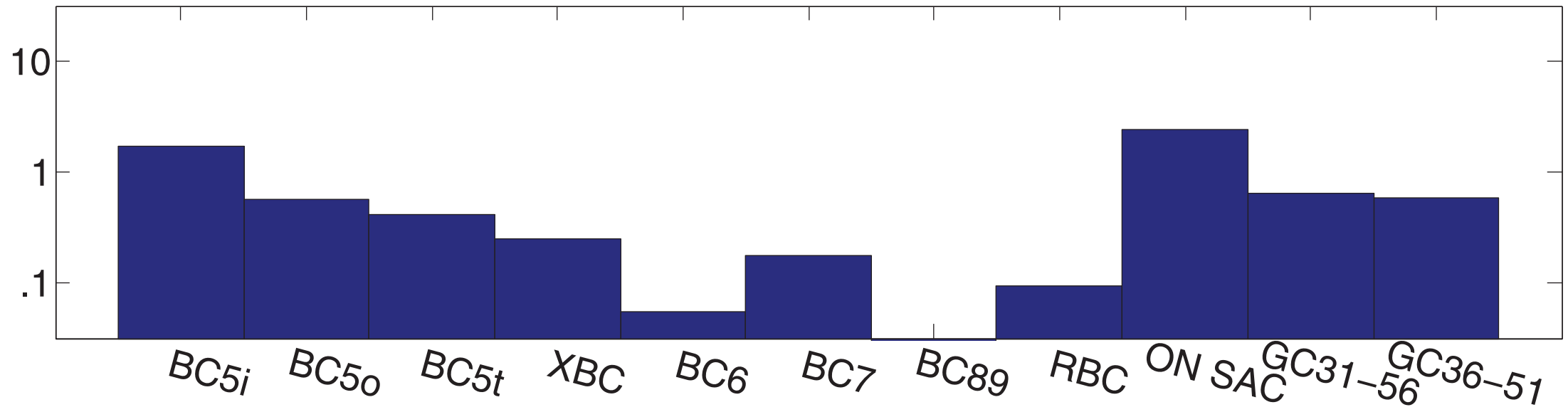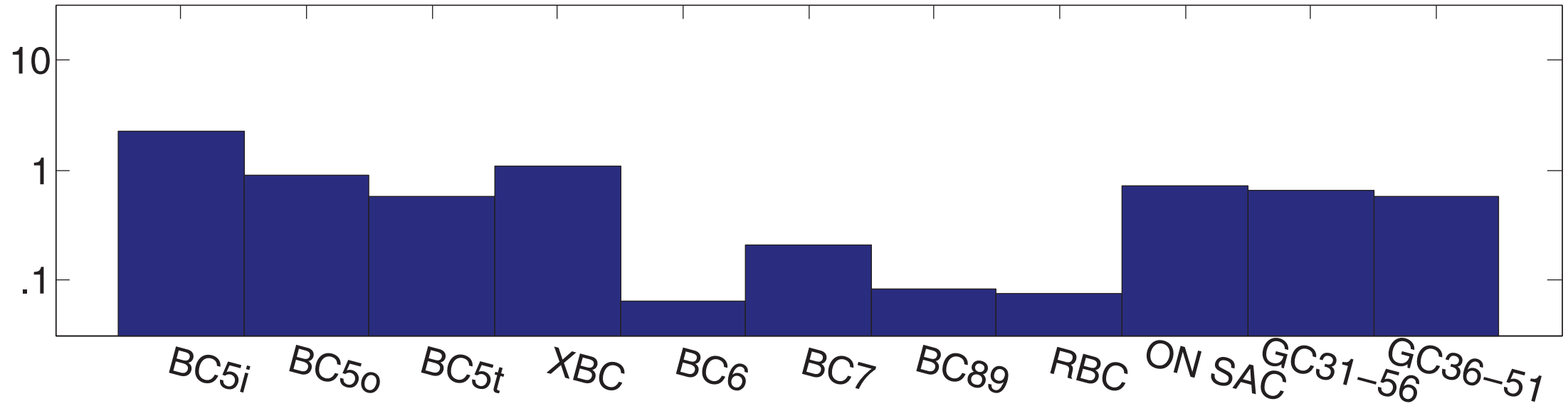

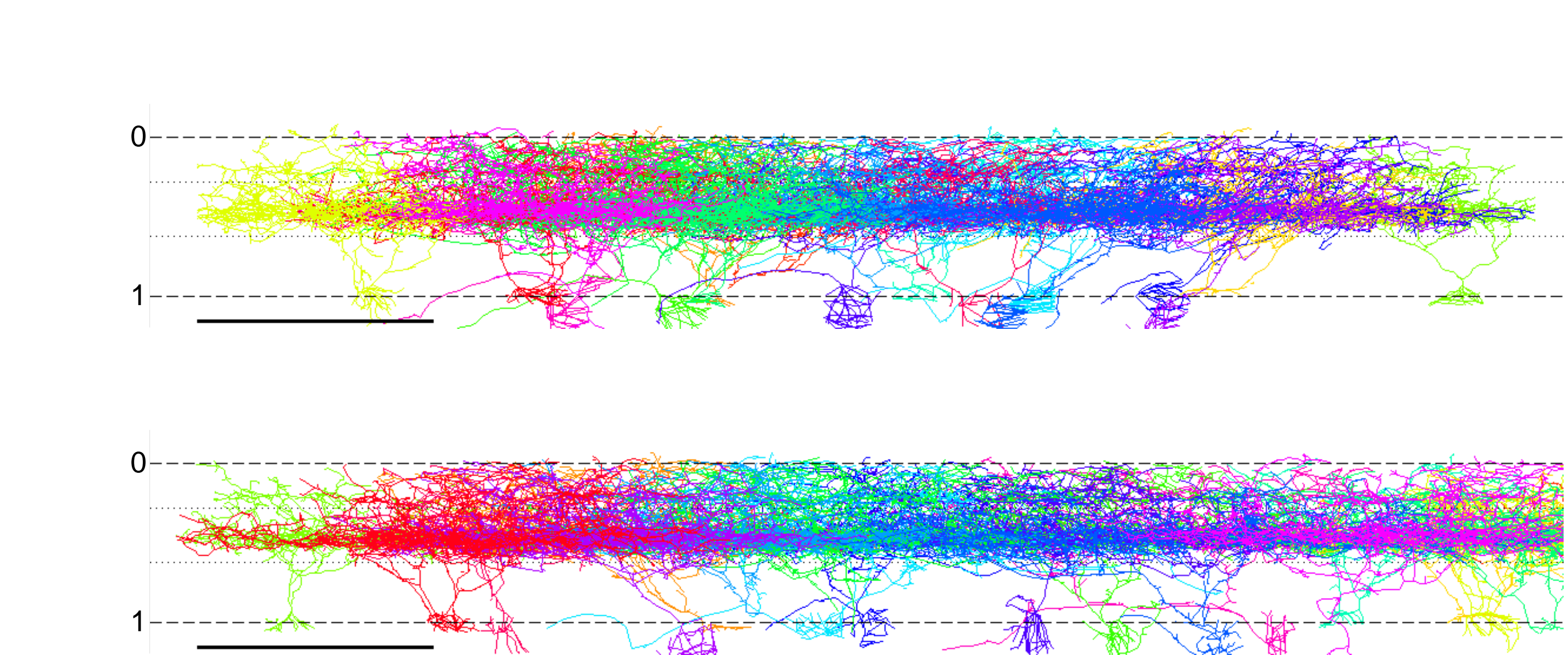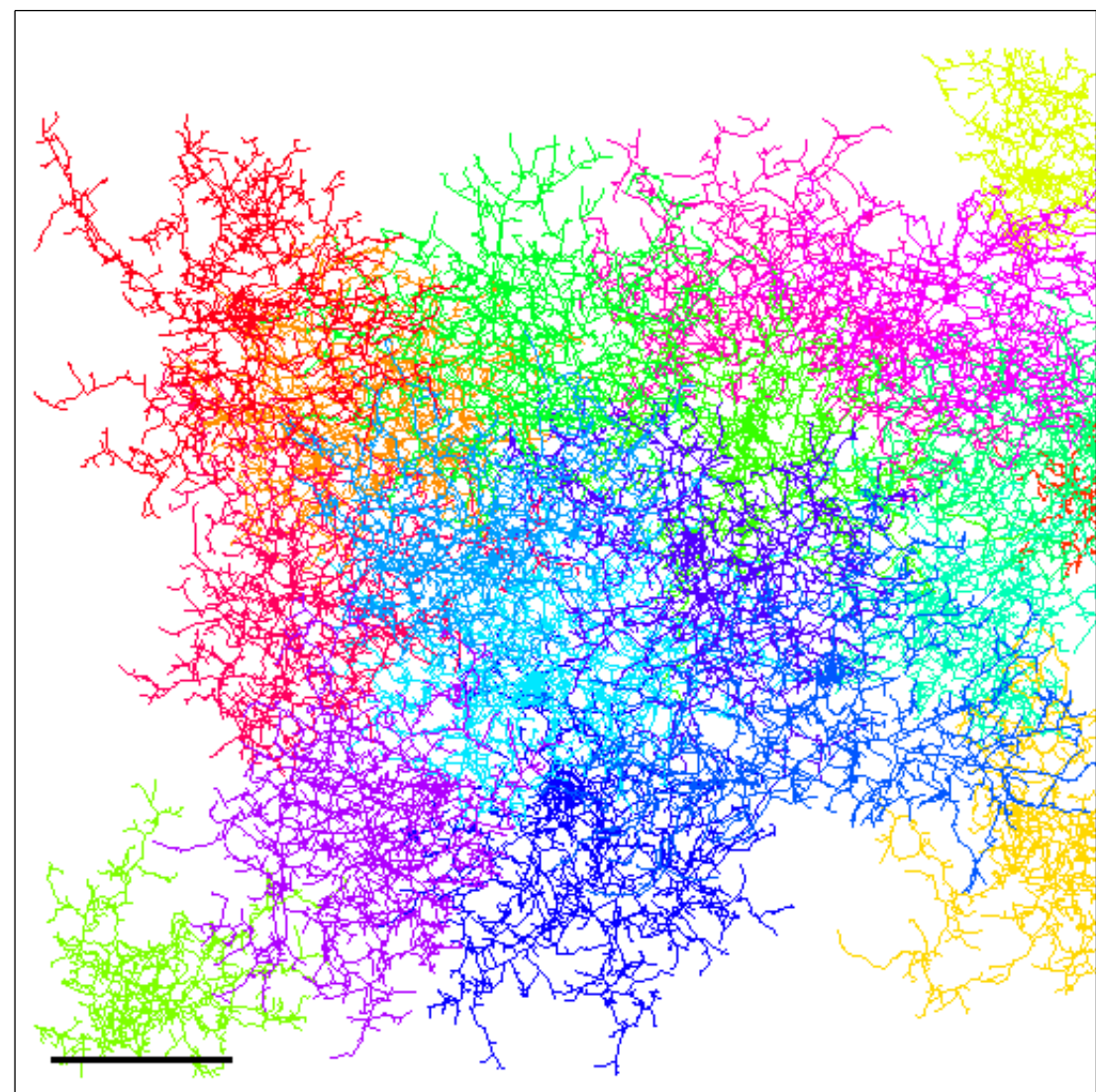

GC36-51

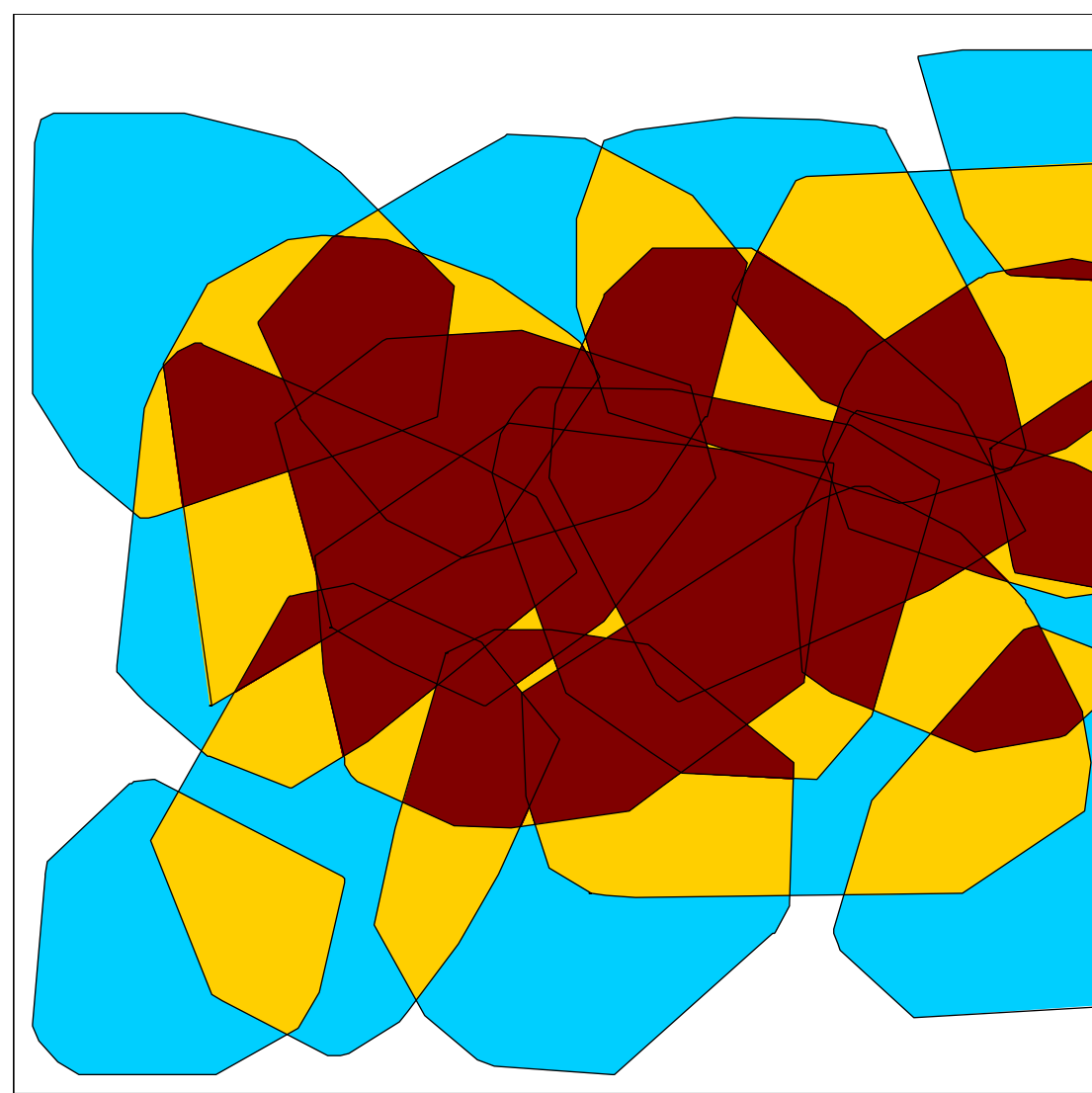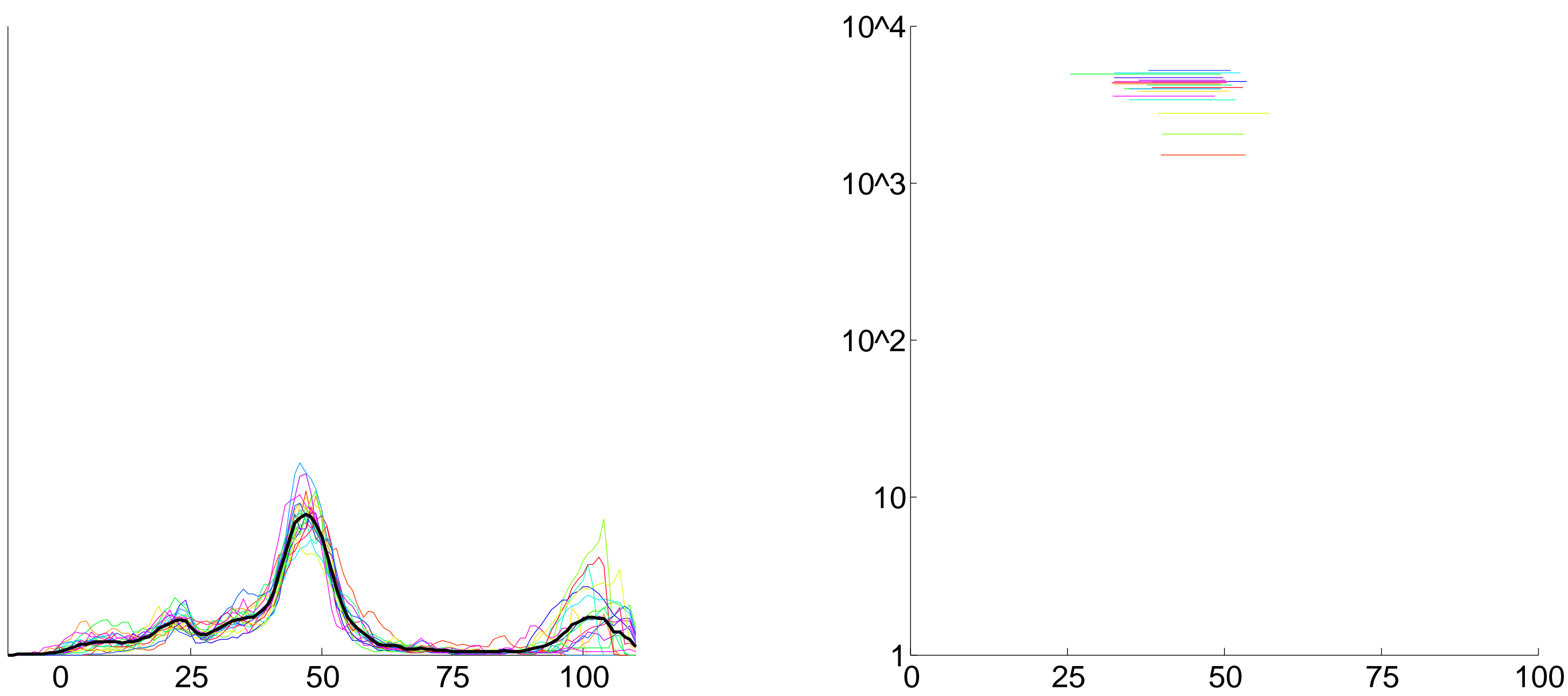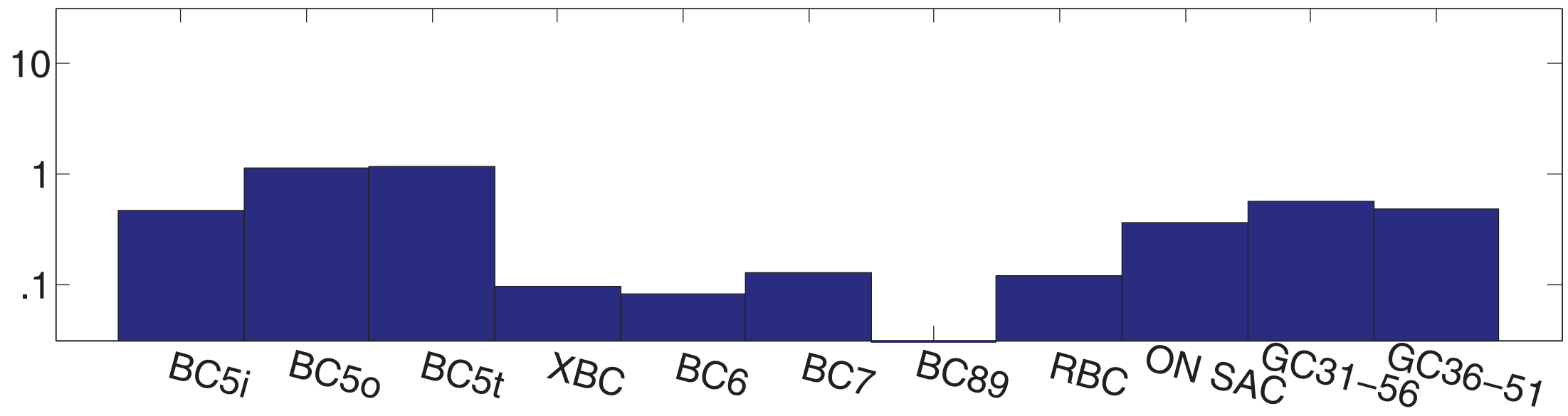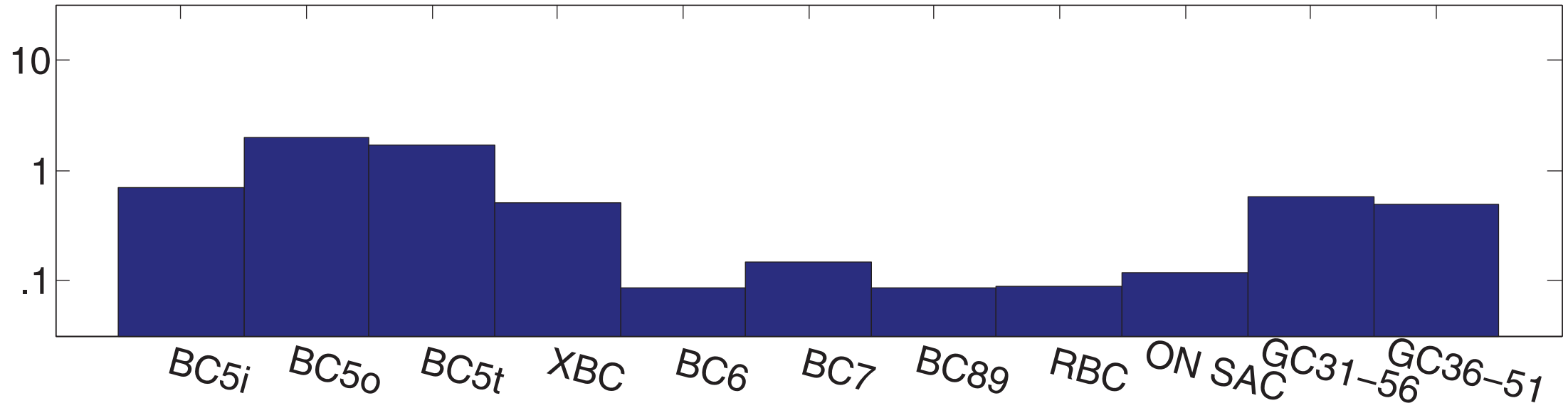

Supplement: 2 [file NIHMS757638-supplement-2.pdf]
